# Dual Co/Photoredox-Catalyzed Regio- and Stereoselective Allyl Homocoupling Providing Functionalized 1,5-Dienes

**DOI:** 10.1021/acs.orglett.6c02074

**Published:** 2026-06-22

**Authors:** Fengyun Gao, Giona Armellin, Stephanie G. E. Amos, Arjan W. Kleij

**Affiliations:** † 202569 Institute of Chemical Research of Catalonia (ICIQ-Cerca), Barcelona Institute of Science and Technology (BIST), Av. Països Catalans 16, 43007 Tarragona, Spain; ‡ 16777 Universitat Rovira i Virgili, C/Marcel·lí Domingo s/n, 43003 Tarragona, Spain; § Catalan Institute for Research and Advanced Studies (ICREA), Pg. Lluis Companys 23, 08010 Barcelona, Spain

## Abstract

Vinyl cyclic carbonates are productive allyl precursors in the generation of functionalized 1,5-dienes featuring a quaternary center using dual Co/organophotoredox catalysis. A bulky N^P^N ligand is key to the exclusive regio- and stereocontrol observed in this formal homocoupling process. Product diversifications highlight the utility of the different functional groups present in the products with respect to site-selective transformations.

## Graphical abstract

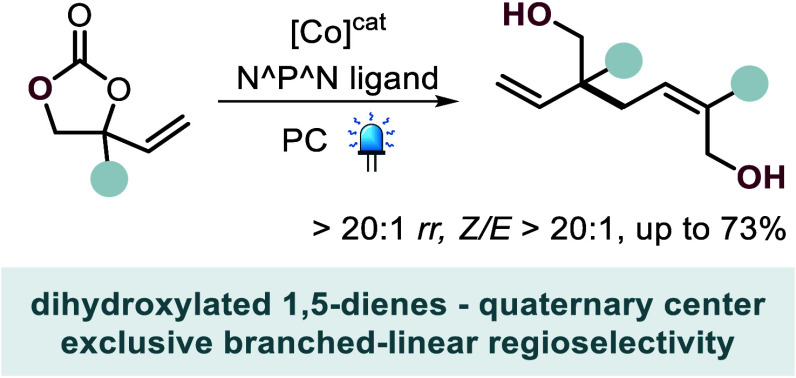

Complex diene scaffolds have long been targets for organic chemists.^1,2^ Their synthetic value lies in the easy and modular alteration of the alkene motifs providing a myriad of useful building blocks. Additionally, in medicinal chemistry^3,4^ and material science,^5,6^ alkene incorporation leads to greater structural rigidity and improved stability. From an organic chemist’s standpoint, dienes exhibit reactivity reminiscent of that of simple alkenes and are prone to, *inter alia*, metathesis, difunctionalization, allylic substitution and functionalization, and radical functionalization.^7–9^ However, their increased π-character also unlocks their potential with respect to pericyclic reactions. Specifically, 1,5-dienes can undergo Cope rearrangements, intramolecular Alder-ene reactions, and oxidative cyclizations.^10–14^ Therefore, accessing functionalized 1,5-diene synthons, currently a particular challenge, is of high and widespread interest. This is, for instance, the case for hydroxylated dienes that have promising applications in industrial settings as surfactants or monomers.^15,16^ In this regard, their most common preparative strategy involves allyl–allyl coupling.

Traditionally, allyl homocoupling reactions proceed through the combination of an electrophilic allyl precursor and a nucleophilic substrate.^17^ This strategy works well for the synthesis of 1,5-dienes but ultimately suffers from the need to synthesize two different activated allyl precursors. Homoallyl coupling is a streamlined alternative approach that builds on a single allyl precursor (Scheme 1a). However, it also represents a keen synthetic challenge as first, three regioisomers can be formed being branched–branched (*bb*), branched–linear (*bl*), and linear–linear (*ll*). Second, for each of these regioisomers, different alkene stereoisomers (*Z* vs *E*) can be formed, and third, both *R*- and *S*-configured stereogenic centers can be forged. Thus, a total of 10 different isomers are possible for a homoallylic coupling process based on a single allylic precursor.

**Scheme 1:**
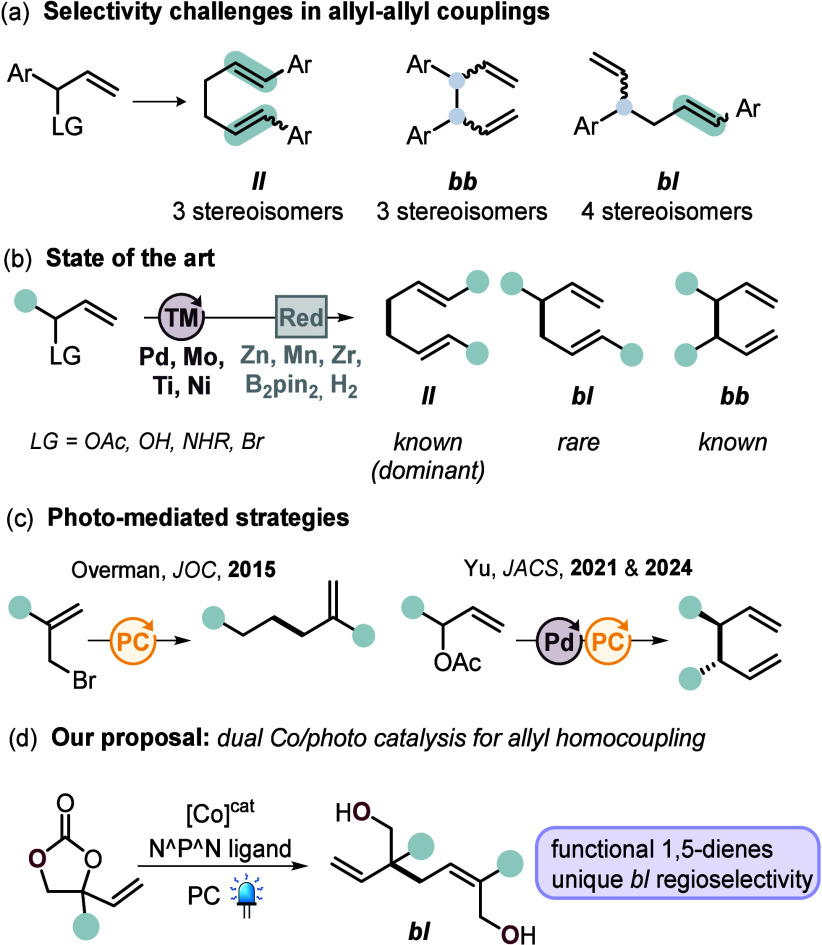
Challenges in Allylic Homocoupling, the State of the Art, and Our Proposal

When considering their synthetic utility, the *bb* and *ll* regioisomeric 1,5-diene products have two identical alkene fragments that may be difficult to selectively transform in postsynthetic utilizations. The third regioisomer (the *bl* product) seems to be the most synthetically valuable one as both alkenes have different electronic and steric environments, and the presence of both allylic and homoallylic alcohols should allow for site-selective modifications. However, the synthesis of *bl* type regioisomers remains rather limited (Scheme 1b). Known strategies for homoallylic coupling require a transition metal catalyst (Pd, Mo, Ti, or Ni) along with a stoichiometric reductant (Zn, Zr, Mn, B_2_pin_2_, or H_2_) and afford primarily the *ll* type regioisomer.^18–24^ To the best of our knowledge, only three examples of photomediated allyl–allyl coupling processes have been reported (Scheme 1c).^25–27^ In 2015, Overman and co-workers reported a Ru­(bpy)_3_^2+^-promoted and Hantzsch ester (HE)-mediated allyl homocoupling.^27^ In 2021 and 2024, Yu and co-workers used an iridium photocatalyst and palladium catalysis to generate *bb* type regioisomeric products with excellent enantiocontrol.^25,26^

In 2023, our group developed an enantioselective homocoupling of allyl-Boc derivatives under Ni catalysis using Zn as a stoichiometric reductant representing the first *bl* type homocoupling to afford compounds with a quaternary stereocenter.^18^ We also reported dual base metal- and photoredox-mediated transformations with vinyl cyclic carbonates (VCCs) (Scheme 1d) and analogues.^28–32^ Combining the aforementioned expertise, we herein describe a *bl* regio- and stereoselective allylic homocoupling of VCCs using dual cobalt/photocatalysis to afford functionalized 1,5-dienes featuring alcohol groups.

We started our studies using the phenyl-substituted VCC (**1a**) and rapidly identified cobalt­(II) acetate, 4CzIPN as the photocatalyst, DIPEA as the additive, **L1** as the ligand, and acetonitrile as the medium to ensure a clean reaction profile with minimal side products (Table 1, entry 1). However, under these conditions, the regioselectivity was poor, favoring *ll-* **2a** over *bl-* **2a** (1.3:1). At this stage, we could also detect the formation of diene **3** that we had previously observed,^30^ and **4**, a known allyl alcohol (see the Supporting Information).^33^ To attain greater regio- and chemoselectivity, we investigated the influence of the ligand scaffold. We found that terpyridines **L2**–**L4** afforded a cleaner reaction profile and higher product yields and hampered the formation of **3**, albeit the regioselectivity remained virtually unaffected (entries 2–4, 68–85% NMR yields, *bl*:*ll* up to 1:2, see the Supporting Information for additional screening data with **L4**). Following the reports by Li and co-workers,^34^ showing the great versatility of N^P^N tridentate ligands, we sought to synthesize and test this class of ligand (entries 5 and 6). The use of both **L5** (R^2^ = H) and **L6** (R^2^ = OMe) afforded desired product *bl*-**2a** in 62% and 54% yields, respectively, with exclusive regioselectivity (>20:1). The diastereoselectivity was determined to be >20:1, showing that only the *Z* internal alkene was present as determined by 2D NMR (see the Supporting Information). We continued our studies using **L6** (as it is less prone to oxidation than **L5**, see the Supporting Information for details). The yield of **2a** could be further increased by cooling the reactor to 10 °C (71%, entry 7). When increasing to a scope scale (0.4 mmol of **1a**), we were able to decrease the cobalt loading to 5 mol % (entry 8), maintaining a 71% yield and exclusive regioselectivity. A final solvent screening confirmed that MeCN was indeed the optimal choice (entries 9 and 10), though the use of DMA also proved to be productive (65% yield). To confirm that **L6** was indeed the optimized ligand, we ran a final test with freshly prepared **L5** under the optimized conditions (10 °C, MeCN), affording **2a** in a slightly lower yield (65%).

**Table 1:**
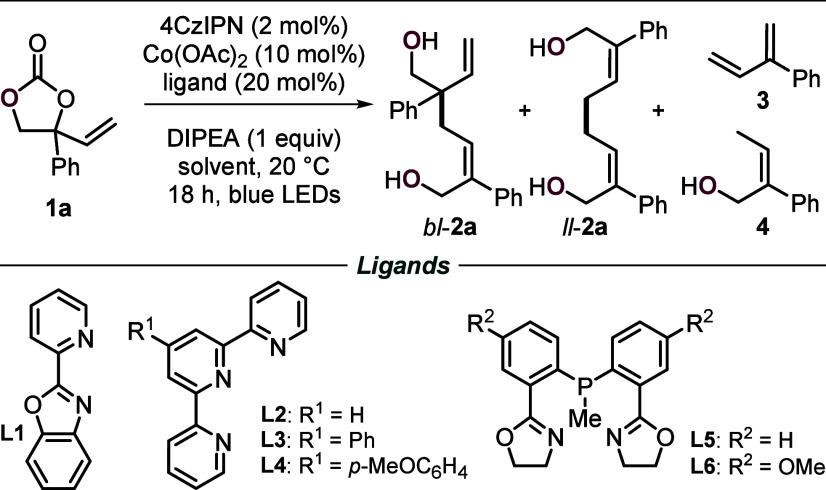
Optimization of the Homocoupling of **1a** ^a^

entry	ligand	solvent	yield (%)^b^	*bl*:*ll* ^b^
1	**L1**	MeCN	34	1:1.3
2	**L2**	MeCN	68	1:1.4
3	**L3**	MeCN	85	1:1.4
4	**L4**	MeCN	75	1:2
5	**L5**	MeCN	62	>20:1
6	**L6**	MeCN	54	>20:1
7	**L6**	MeCN	71	>20:1
8^c^ ^,^ ^d^	**L6**	MeCN	71	>20:1
9^c^ ^,^ ^d^	**L6**	DMA	65	>20:1
10^c^ ^,^ ^d^	**L6**	PhMe	nd^e^	>20:1
11^c^ ^,^ ^d^	**L5**	MeCN	65	>20:1

a Reaction conditions: **1a** (0.2 mmol), 4CzIPN (2 mol %), Co­(OAc)_2_ (10 mol %), ligand (20 mol %), and DIPEA (0.2 mmol) in a solvent (0.2 M). Irradiated for 18 h using a single high-power blue LED (451 nm, 1 W).

b Determined by ^1^H NMR (CDCl_3_) using mesitylene (14 μL, 0.10 mmol) as an internal standard.

c Reaction performed at 10 °C.

d Reaction on a scope scale: **1a** (0.4 mmol), 4CzIPN (2 mol %), Co­(OAc)_2_ (5 mol %), a ligand (10 mol %), and DIPEA (0.4 mmol) in a solvent (0.4 M). Irradiated at 10 °C for 18 h using a single high-power blue LED (451 nm, 1 W).

e N.d. stands for not detected, 25% conversion.

With the optimized conditions in hand, we investigated the scope of the transformation and were pleased to obtain all products as a single regioisomer and *Z* diastereomer (*rr* and *dr* values of >20:1, Scheme 2). The model substrate was converted into 1,5-diene product **2a** in 71% yield,^35^ whereas *p*-methyl and *p*-*t*Bu substitutions in the aryl group of the VCC led to the isolation of the corresponding 1,5-dienes in 70% and 76% yields, respectively. Halogen substitutions in the VCC substrate were tolerated, affording *p*-fluoro derivative **2d** in 73% yield and **2e** and **2f** in 59% and 50% yields, respectively. Substrates with strong electron-donating groups performed poorly, delivering a 22% yield of **2g** (*p*-OMe) and a 40% yield of the *p*-OBn derivative (**2h**). Product **2g** was obtained in 31% yield with **L5**. The presence of an electron-poor aryl group in the VCC (cf., *p*-CF3) afforded product **2i** in only 21% yield. Finally, we were able to obtain **2j**, with a *p*-Ph substitution, although its poor solubility led to an average 30% yield. Product **2j** was analyzed via X-ray diffraction, further confirming the proposed *Z* configuration of the trisubstituted alkene group. Substitution at the *meta* position was well-tolerated for moderately withdrawing or donating groups, and their conversion led to the products having a *m*-Me (**2k**) in 67% yield, a *m*-OMe (**2l**) in 73% yield, and a *m*-OBn (**2m**) in 51% yield. An *m*-CF_3_ substitution was less productive and gave the product in 26% yield.

**Scheme 2:**
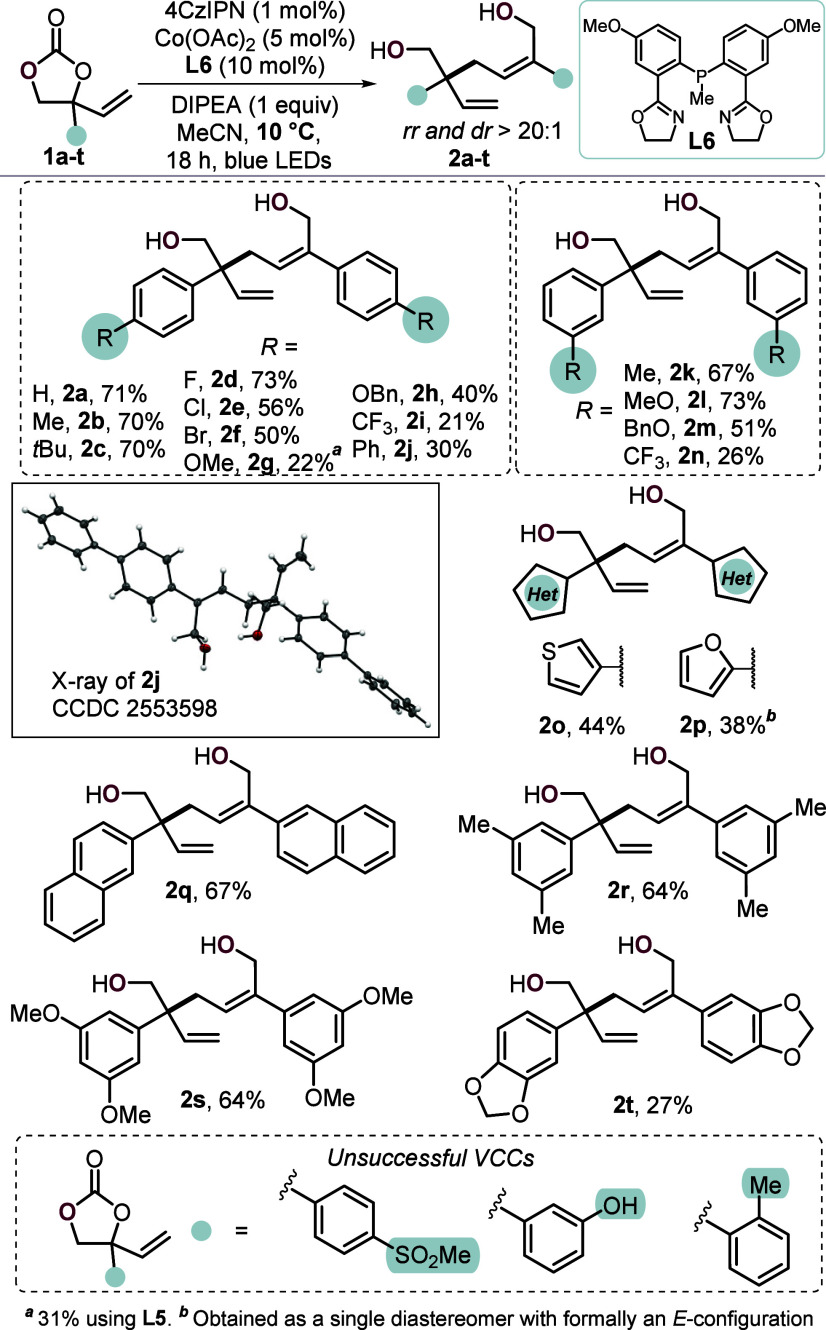
Scope of VCCs

The transformation of VCCs with heterocyclic substituents was also possible and furnished desired compound **2o** in 44% yield (70%, brsm) and **2p** in 38% yield (furan-2-yl, formally the *E* isomer, see the Supporting Information). Finally, we further varied the aromatic substituents of the VCC, thereby giving access to naphthyl derivative **2q** in 67% yield and products having a 3,5-disubstitution (**2r**, dimethyl; **2s**, dimethoxy), which were isolated in 69% and 64% yields, respectively. Lastly, the catechol derivative (**2t**) was obtained in 28% yield. Although the reaction presented some limitations regarding *ortho* substituents and free alcohols, overall, the process tolerates mildly polarizing and various substitution patterns in the VCC. We suspect that the lower yields observed for strongly polarized substrates result from decomposition (see the Supporting Information for further details).

Having explored the scope of the transformation, we turned toward the value of our functionalized 1,5-dienes (Scheme 3). First, our method could successfully be scaled up to 1.0 mmol to deliver 0.171 g of **2a** (58% isolated yield, 85% conversion; i.e., 68% brsm). The lower conversion is likely due to our experimental setup, which irradiates from below; hence, light penetration is less efficient. Then, we prepared diacetylated **5** (98% yield), and it could be selectively transformed into epoxy derivative **6** (37%) using *m*-CPBA. The lower yield is a result of the sensitivity of **6** to the purification process.

**Scheme 3:**
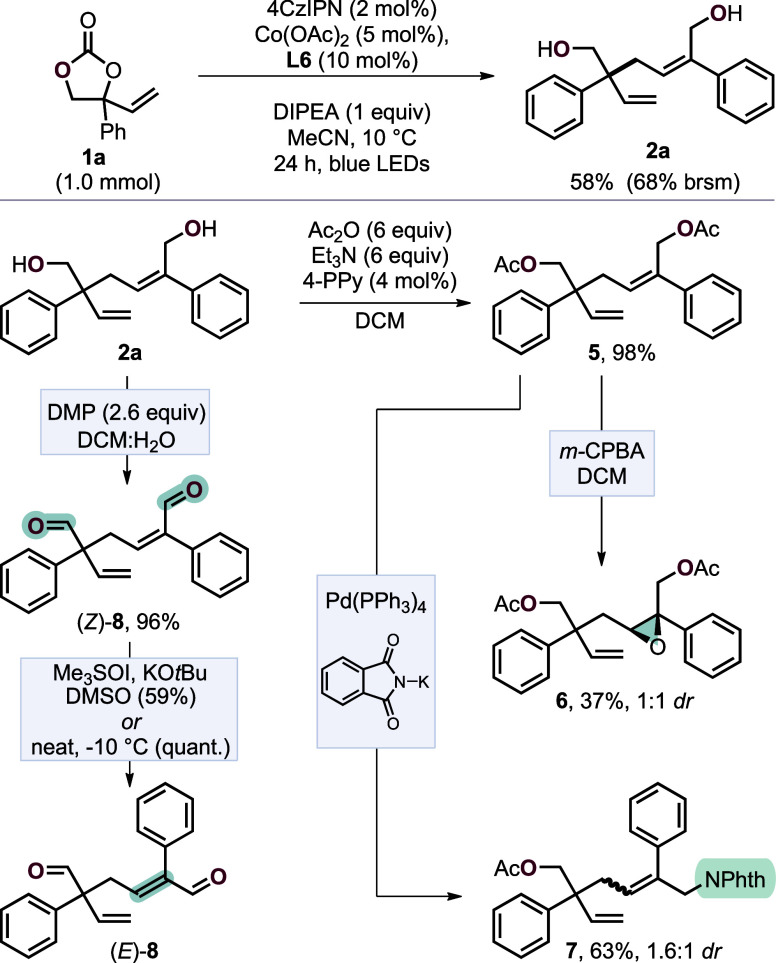
Scale-up and Product Modifications

Diacetylated compound **5** could also be used as an allylic precursor in a formal Pd-catalyzed allylic amination using potassium phthalimide.^36^ Desired aminated product **7** was obtained in 63% yield as a 1.6:1 mixture of *E* and *Z* diastereomers. Diol **2a** was also successfully converted into dialdehyde *Z*-**8** in 96% yield by utilizing DMP. Finally, we observed the rapid isomerization of *Z*- to *E*-**8** under Corey–Chaykovsky cyclopropanation conditions (59% yield), which could also occur by storing the neat initial product for 2 weeks in a refrigerator.

To interrogate the mechanism of the transformation, we performed standard control reactions (see Scheme 4a). In the absence of light or cobalt and a ligand, no conversion of the substrate was detected. In the absence of the photocatalyst, 15% conversion of **1a** was observed; however, neither **2a**, **3**, **4**, nor other products were detected. We noted, under the standard conditions in the absence of a ligand (Scheme 4b), 30% conversion of the VCC to allylic alcohol **4** (28% yield). This indicates that the presence of the ligand is the key to preventing parasitic consumption of the substrate. We then investigated the loading of DIPEA (Scheme 4c), finding that both sub- and superstoichiometric loadings led to a lower product yields and chemoselectivity. Without DIPEA, although 20% conversion was observed, **2a** was not detected, confirming that DIPEA is key for productive catalysis.

**Scheme 4:**
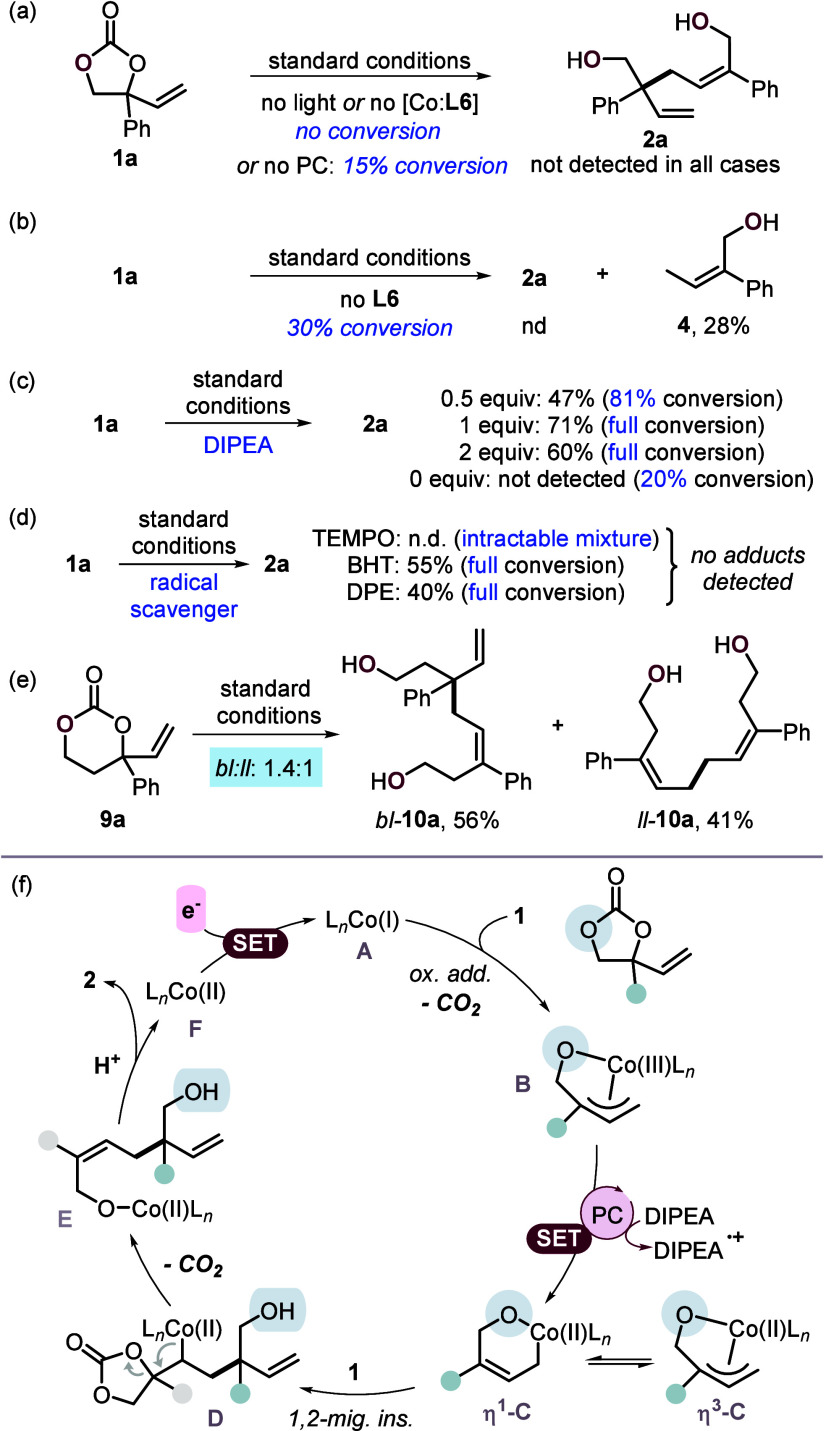
Control Reactions and Postulated Catalytic Cycle

Additionally, Et_3_N was incompatible with the optimized process, suggesting that the structure of DIPEA may play an important role in this mechanism, further illustrating the fine balance of the reductive conditions this transformation requires. Next, we speculated that the VCC could act as a source of the allyl radical under the dual catalytic conditions (Scheme 4d).^28^ The presence of TEMPO led to an intractable mixture containing residual VCC **1a**. BHT and DPE (as additives) did not impact substrate conversion. However, in these last two cases, we observed greater side product formation and lower yields of **2a**. In all cases, we were unable to detect allylated adducts and thus did not find evidence for the presence or formation of an allyl-based free radical species. Finally, when increasing the ring size of the VCC (**9a**, Scheme 4e) from five to six, we observed a significant decrease in the regiocontrol of the process (1.4:1 *bl*:*ll*, products **10**). This could be explained by a weaker preference for a single cobalt­(II) allyl species (Scheme 4f). Hence, the size of the cobaltacycle is likely key to controlling the regioselectivity.

Based on our experimental data and literature precedent,^29,32,37,38^ we propose the following catalytic pathway (Scheme 4f). Starting with **A**, an oxidative addition of VCC **1** generates Co­(III)-π-allyl intermediate **B**. We surmise that this intermediate is then reduced to a Co­(II) allyl being a η^3^-**C** or η^1^-**C** as previously established.^27,30^ Following the observed regioselectivity, we envision a 1,2-migratory insertion type process combining **C** with a second molecule of VCC allowing the formation of **D** that features a quaternary stereocenter. β-Oxy elimination then affords exclusively a *Z*-configured alkene followed by decarboxylation to give Co­(II) species **E**. Proto-demetalation finally provides desired product **2** and a Co­(II) intermediate. The latter is then reduced via an SET to regenerate the initial Co­(I) complex for further turnover (see the Supporting Information for full details and a complete mechanistic proposal).

In summary, we here present a dual Co/photoredox-catalyzed allyl–allyl homocoupling of VCCs. This stereoselective process proceeds with exclusive and unique *bl* regioselectivity achieved with N^P^N ligand **L6**. Our method affords synthetically useful and challenging 1,5-dienes, the first to deliver functionalized (dihydroxylated) dienes that comprise a quaternary stereocenter. A scope that includes both aryl- and heteroaryl-substituted VCCs was identified, and product diversification illustrates the capacity of these scaffolds to partake in site-selective transformations. A brief mechanistic study confirms the necessity and dual catalytic character of our transformation. The method presented herein presents a simple and user-friendly approach to access complex diene-diols.

## Supplementary Material



## Data Availability

The data underlying this study are available in the published article and its Supporting Information. Raw data for NMR and MS is available free of charge from https://dataverse.csuc.cat at https://doi.org/10.34810/DATA3261.

## Notes

Experimental procedures, full spectroscopic data for all new compounds, and copies of NMR and IR spectra (PDF)

## References

[1] Canfield A. M., Rodina D., Paradine S. M. (2024). Dienes as Versatile Substrates for
Transition Metal-Catalyzed Reactions. Angew.
Chem., Int. Ed..

[2] Chen J.-R., Hu X.-Q., Lu L.-Q., Xiao W.-J. (2015). Formal [4+1] Annulation
Reactions in the Synthesis of Carbocyclic and Heterocyclic Systems. Chem. Rev..

[3] Horsley J. R., Yu J., Moore K. E., Shapter J. G., Abell A. D. (2014). Unraveling the Interplay
of Backbone Rigidity and Electron Rich Side-Chains on Electron Transfer
in Peptides: The Realization of Tunable Molecular Wires. J. Am. Chem. Soc..

[4] Anthony N. G., Breen D., Clarke J., Donoghue G., Drummond A. J., Ellis E. M., Gemmell C. G., Helesbeux J.-J., Hunter I. S., Khalaf A. I., Mackay S. P., Parkinson J. A., Suckling C. J., Waigh R. D. (2007). Antimicrobial Lexitropsins Containing
Amide, Amidine, and Alkene Linking Groups. J.
Med. Chem..

[5] Wu P., Hu Q., Ogunfowora L. A., Li Z., Marquardt A. V., Savoie B. M., Dou L. (2025). Toward Sustainable
Polydienes. J. Am. Chem. Soc..

[6] Coates G. W., Hustad P. D., Reinartz S. (2002). Catalysts
for the Living Insertion
Polymerization of Alkenes: Access to New Polyolefin Architectures
Using Ziegler–Natta Chemistry. Angew.
Chem., Int. Ed..

[7] Takacs J., Jiang X. (2003). The Wacker Reaction
and Related Alkene Oxidation Reactions. Curr.
Org. Chem..

[8] Fürstner, A. , Gibson, S. E. , Eds. Alkene Metathesis in Organic Synthesis; Topics in Organometallic Chemistry; Springer: Berlin, 2001.

[9] Lan X.-W., Wang N.-X., Xing Y. (2017). Recent Advances in Radical Difunctionalization
of Simple Alkenes. Eur. J. Org. Chem..

[10] Müller T. J. J. (2025). Recent
Advances in Ene Reactions with Carbon Enophiles. Adv. Synth. Catal..

[11] Huang G., Dong Y. (2019). Application of Cope Rearrangement in Synthesis. Synth. Commun..

[12] Adrian J., Gross L. J., Stark C. B. W. (2016). The Direct Oxidative
Diene Cyclization
and Related Reactions in Natural Product Synthesis. Beilstein J. Org. Chem..

[13] Mikami K., Shimizu M. (1992). Asymmetric Ene Reactions in Organic Synthesis. Chem. Rev..

[14] Lutz R. P. (1984). Catalysis
of the Cope and Claisen Rearrangements. Chem.
Rev..

[15] Alany R. G., Rades T., Agatonovic-Kustrin S., Davies N. M., Tucker I. G. (2000). Effects
of Alcohols and Diols on the Phase Behaviour of Quaternary Systems. Int. J. Pharm..

[16] Minnich, K. E. Trans Olefinic Diols with Surfactant Properties. EP 0940169A1, 1999.

[17] Ravichandiran V., Jana A. (2022). Recent Development
of Allyl–Allyl Cross-Coupling and Its Application
in Natural Product Synthesis. Org. Chem. Front..

[18] Ghorai D., Garcia-Roca A., Tóth B. L., Benet-Buchholz J., Kleij A. W. (2023). Ni-Catalyzed Regio-
and Enantioselective Homoallylic
Coupling: Synthesis of Chiral Branched 1,5-Dienes Featuring a Quaternary
Stereogenic Center and Mechanistic Analysis. Angew. Chem., Int. Ed..

[19] Zhou X., Zhang G., Huang R., Huang H. (2021). Palladium-Catalyzed
Allyl–Allyl Reductive Coupling of Allylamines or Allylic Alcohols
with H2 as Sole Reductant. Org. Lett..

[20] Gan Y., Hu H., Liu Y. (2020). Nickel-Catalyzed
Homo- and Cross-Coupling of Allyl
Alcohols via Allyl Boronates. Org. Lett..

[21] Barrero A. F., Herrador M. M., Quílez
del Moral J. F., Arteaga P., Arteaga J. F., Diéguez H. R., Sánchez E. M. (2007). Mild TiIII-
and Mn/ZrIV-Catalytic Reductive Coupling of Allylic Halides: Efficient
Synthesis of Symmetric Terpenes. J. Org. Chem..

[22] Barrero A. F., Herrador M. M., Quílez
del Moral J. F., Arteaga P., Arteaga J. F., Piedra M., Sánchez E. M. (2005). Reductive
Coupling of Terpenic Allylic Halides Catalyzed by Cp_2_TiCl:
A Short and Efficient Asymmetric Synthesis of Onocerane Triterpenes. Org. Lett..

[23] Masuyama Y., Otake K., Kurusu Y. (1987). Hexacarbonylmolybdenum­(0)-Catalyzed
Reductive Coupling of Allylic Acetates. Bull.
Chem. Soc. Jpn..

[24] Sasaoka S., Yamamoto T., Kinoshita H., Inomata K., Kotake H. (1985). Palladium-Catalyzed
Coupling of Allylic Acetates with Zinc. Chem.
Lett..

[25] Li B., Zhang H.-H., Luo Y., Yu S., Goddard
III W. A., Dang Y. (2024). Interception of Transient Allyl Radicals
with Low-Valent Allylpalladium Chemistry: Tandem Pd­(0/II/I)–Pd­(0/II/I/II)
Cycles in Photoredox/Pd Dual-Catalytic Enantioselective C­(sp^3^)–C­(sp^3^) Homocoupling. J.
Am. Chem. Soc..

[26] Zhang H.-H., Tang M., Zhao J.-J., Song C., Yu S. (2021). Enantioselective
Reductive Homocoupling of Allylic Acetates Enabled by Dual Photoredox/Palladium
Catalysis: Access to C2-Symmetrical 1,5-Dienes. J. Am. Chem. Soc..

[27] Pratsch G., Overman L. E. (2015). Synthesis of 2,5-Diaryl-1,5-Dienes from Allylic Bromides
Using Visible-Light Photoredox Catalysis. J.
Org. Chem..

[28] Zeng Q., Gao F., Benet-Buchholz J., Kleij A. W. (2023). Stereoselective
Three-Component Allylic Alkylation Enabled by Dual Photoredox/Ni Catalysis. ACS Catal..

[29] Limburg B., Cristòfol À., Kleij A. W. (2022). Decoding Key Transient
Inter-Catalyst Interactions in a Reductive Metallaphotoredox-Catalyzed
Allylation Reaction. J. Am. Chem. Soc..

[30] Xue S., Cristòfol À., Limburg B., Zeng Q., Kleij A. W. (2022). Dual Cobalt/Organophotoredox
Catalysis for Diastereo-
and Regioselective 1,2-Difunctionalization of 1,3-Diene Surrogates
Creating Quaternary Carbon Centers. ACS Catal..

[31] Xue S., Limburg B., Ghorai D., Benet-Buchholz J., Kleij A. W. (2021). Asymmetric Synthesis of Homoallylic
Alcohols Featuring
Vicinal Tetrasubstituted Carbon Centers via Dual Pd/Photoredox Catalysis. Org. Lett..

[32] Cristòfol À., Limburg B., Kleij A. W. (2021). Expedient
Dual Co/Organophotoredox
Catalyzed Stereoselective Synthesis of All-Carbon Quaternary Centers. Angew. Chem., Int. Ed..

[33] Denmark S. E., Pan W. (2003). Intramolecular *Syn* and *Anti* Hydrosilylation
and Silicon-Assisted Cross-Coupling: Highly Regio- and Stereoselective
Synthesis of Trisubstituted Allylic Alcohols. Org. Lett..

[34] Ghorai S., Ur Rehman S., Xu W.-B., Huang W.-Y., Li C. (2020). Cobalt-Catalyzed
Regio- and Enantioselective Allylic Alkylation of Malononitriles. Org. Lett..

[35] Apart from main product **2a**, we also typically found 2-aryl-1,3-diene byproduct **3** (see ref 30) and formally decarboxylated allylic alcohol **4** (Table 1 and Scheme 4b) that affect the product yield. A crude ^1^H NMR spectrum has been provided with assignments in the Supporting Information.

[36] Komanduri V., Krische M. J. (2006). Enantioselective Reductive Coupling of 1,3-Enynes to
Heterocyclic Aromatic Aldehydes and Ketones via Rhodium-Catalyzed
Asymmetric Hydrogenation: Mechanistic Insight into the Role of Brønsted
Acid Additives. J. Am. Chem. Soc..

[37] Takizawa K., Sekino T., Sato S., Yoshino T., Kojima M., Matsunaga S. (2019). Cobalt-Catalyzed
Allylic Alkylation Enabled by Organophotoredox
Catalysis. Angew. Chem., Int. Ed..

[38] Wu X., Shi Y., Zhang C., Xu K., Xia T., Huang G., Qu J., Chen Y. (2026). Enantio- and
linear-selective allylic radical addition
to imines using reductive cobalt catalysis. Nat. Synth..

