# Supplementary material for: Dual Co/Photoredox-Catalyzed Regio- and Stereoselective Allyl Homocoupling Providing Functionalized 1,5-Dienes
Source: Org Lett. 2026 Jun 22;28(26):8382–7. doi: 10.1021/acs.orglett.6c02074 (PMC13339761; doi:10.1021/acs.orglett.6c02074)

Supporting Information for:

Dual Co/Photoredox Catalyzed Regio– and Stereoselective Allyl Homocoupling providing Functionalized 1,5-Dienes

Fengyun Gao[a,b‡] Giona Armellin[a,b‡] Stephanie G. E. Amos[a*] and Arjan W. Kleij[a,c*]

[a] Institute of Chemical Research of Catalonia (ICIQ-Cerca), Barcelona Institute of Science and Technology (BIST), Av. Països Catalans 16, 43007 Tarragona, Spain. * Email: samos@iciq.es, akleij@iciq.es

[b] Universitat Rovira i Virgili, C/Marcel·lí Domingo *s/n*, Tarragona, Spain

[c] Catalan Institute for Research and Advanced Studies (ICREA), Pg. Lluis Companys 23, 08010 Barcelona, Spain

‡ These authors contributed equally

Contents:

1. General information....................4

2. Photocatalytic set-up....................6

3. Experimental procedures....................7

General procedure 1 (**GP1**): Branched-linear homocoupling of VCCs....................7

Structural determination: NOESY of product **2a**....................8

Experimental procedure: Homocoupling of **1a** with **L4**....................9

Experimental procedure: Homocoupling of 6-VCC **9a** with **L4**....................10

General procedure (**GP2**) for ligand synthesis (**L5** and **L6**)....................11

Aryl bromide (SI-4) synthesis....................11

Lithiation-phosphorylation-methylation involving SI-4....................13

General procedure 2 (**GP3**): Vinyl cyclic carbonate synthesis....................15

Scale-up of product **2a**....................17

Product modifications....................18

Acylation....................18

Epoxidation....................19

Pd catalyzed amination....................19

Alcohol oxidation....................21

Alkene isomerization....................22

4. Optimization....................24

General procedure (**GP4**) for screening:....................24

Crude ^{1}H NMR (400 MHz, $CDCl_3$) of Table 1 entry 1 (using **L1**)....................25

Full ligand screening with product NMR yields (%)....................26

Screening with commercial ligand **L4**....................27

Unsuccessful substrates....................28

Optimization of **L6** synthesis....................29

5. Mechanistic discussion....................30

Rationale for lower yields....................30

Electronic effects of the ligand....................32

Control reactions 33

Homocoupling of a 6VCC using **L6** 34

Crossover experiment 34

Complete mechanistic proposal 35

6. Characterization data for all new compounds 37

7. X-ray molecular structure of **2j** (CCDC-2553598) 52

8. NMR spectra for all new compounds 53

1. General information

Reagents and Solvents

Commercially available reagents and solvents were purchased from Sigma-Aldrich, TCI, Fluorochem, Strem Chemicals, ABCR GmbH, Acros Organics, Alfa Aesar or BCD and were used without further purification unless mentioned. THF, Et_2O and CH_2Cl_2 were dried by passage over activated alumina under nitrogen atmosphere (H_2O content < 10 ppm, determined by Karl-Fischer titration) using an Innovative Technology PURE SOLV solvent purification system or MeCN, DMF, DMA, and other solvents were purchased anhydrous on molecular sieves from the suppliers. Deionized water was degassed by bubbling Argon through 10 mL for 30 min in an ultrasound bath and kept for a maximum of 30 days. No differences were seen using freeze-pump-thaw to degas the water. 4CzIPN (the organo-photocatalyst) was synthesized following a known procedure.[1]

Methods

When necessary and when stated in the reaction procedure, air- and water-sensitive reactions were carried out in oven-dried glassware under an argon (Ar) atmosphere using standard Schlenk techniques. Reactions were monitored by TLC and/or ^1H NMR spectroscopy. TLC was carried out on 0.25 mm Merck aluminum-backed sheets coated with 60 F_{254} silica gel and Al_2O_3 visualized with UV light (254 nm), permanganate stain, ceric ammonium chloride stain, and p-anisaldehyde stain ($EtOH:H_2SO_4:AcOH$:*p*-anisaldehyde 135:5:1.5:3.7 v:v:v:v). Flash chromatography was carried out with Seculpo silica gel 60 (70-230 mesh) or neutral Al_2O_3 using the indicated eluent system.

Analytical Techniques

^1H, ^{13}C, ^{19}F and ^{31}P NMR spectra were recorded at room temperature on a Bruker AV-300, AV-400, or AV-500 spectrometer. ^{13}C, ^{19}F and ^{31}P NMR were measured with ^1H-decoupling. All signals are reported in ppm and referenced to their internal solvent signals (^1H NMR $CDCl_3$: 7.26 ppm, DMSO-d_6: 2.50 ppm, CD_3CN: 1.94 ppm; ^{13}C NMR, $CDCl_3$: 77.0 ppm, DMSO-d_6: 39.5 ppm, CD_3CN: 1.32 ppm). Coupling constants (*J*) are reported in hertz with the following splitting abbreviations: s = singlet, d = doublet, t = triplet, q = quadruplet, quint = quintet, sextet = sext, heptet = hept, bs = broad signal and

(1) Engle, S. M.; Kirkner, T. R.; Kelly, C. B. *Org. Synth.* **2019**, *96*, 455-473. https://doi.org/10.15227/orgsyn.096.0455.

app = apparent. High-resolution mass spectrometric analyses were measured using a MicroTOF Focus, Bruker Daltonics with ESI or APCI ionization and performed by the High-Resolution Mass Spectrometry (HRMS) Unit of the ICIQ. X-ray analyses were performed by the Research Support Group at ICIQ using a Rigaku MicroMax-007HF Single Crystal X-ray Diffractometer equipped with a Pilatus 200K area detector, a Rigaku MicroMax- 007HF microfocus rotating anode with MoKα radiation, Confocal Max Flux optics and an Oxford Cryosystems low temperature device Cryostream 700 plus (T = -173 °C).

Abbreviations:

n.d.: Not detected
brsm: Based on remaining starting material

2. Photocatalytic set-up

The photocatalyzed reactions were performed in a parallel photoreactor with 8 wells for 10 mL dram vials (Figure S1 - left). The reactions were thermostated using a Heidolph Rotacool (ethylene glycol:water 50:50) which is circulated through the 8-well reaction block. Each reaction is irradiated by a single LED through a plate of plexiglass (3 mm). The LEDs used were OSRAM Oslon SSL 80 royal-blue LED mounted on a star PCB (I_F = 350 mA; T_J = 25 °C: λ_{peak} = typ. 451 nm and λ_{dom} = 439 - 457 nm, current range: I = 100 – 1000 mA, current used: 700 mA, Radiant flux: 1032 mW). The LEDs are mounted in parallel on a metallic circular heatsink and powered by a single current limited power supply (RS Pro RS-3005D, Figure S1 - right). The whole reactor is placed on a stirring plate. For a visual, see below.

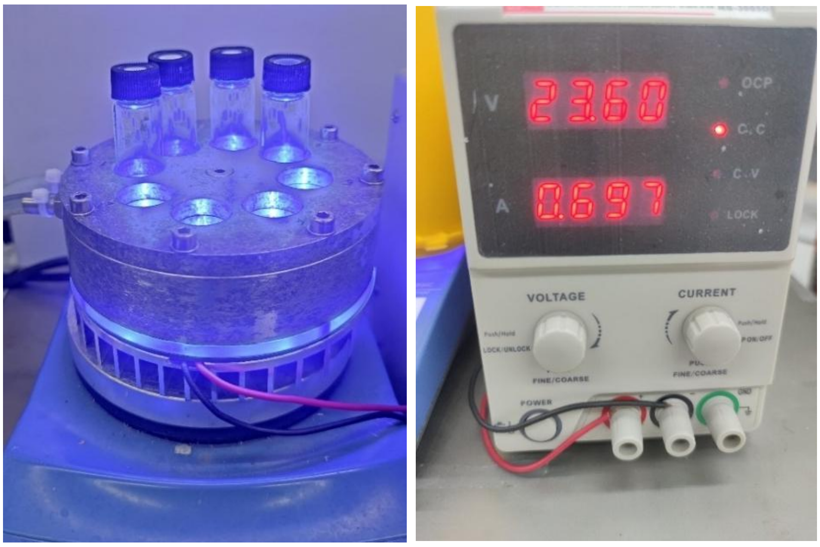

Figure S1. 8-well parallel photoreactor set-up. Left: photograph of the stirring plate and block, right: photograph of the power supply.

Table S1. Radiant flux values dependent on intensity.

Source	I (mA)	Radiant flux (mW)
Ref 2	100	150
Ref 2	350	500
Ref 2	1000	1480
Calculation	***700***	***1032***

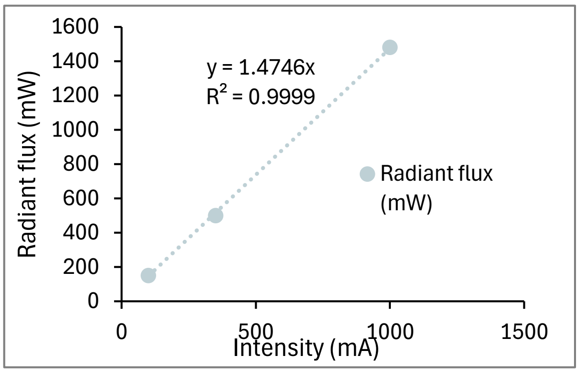

Figure S2. Linear regression radiant flux in function of intensity

3. Experimental procedures

General procedure 1 (**GP1**): Branched-linear homocoupling of VCCs

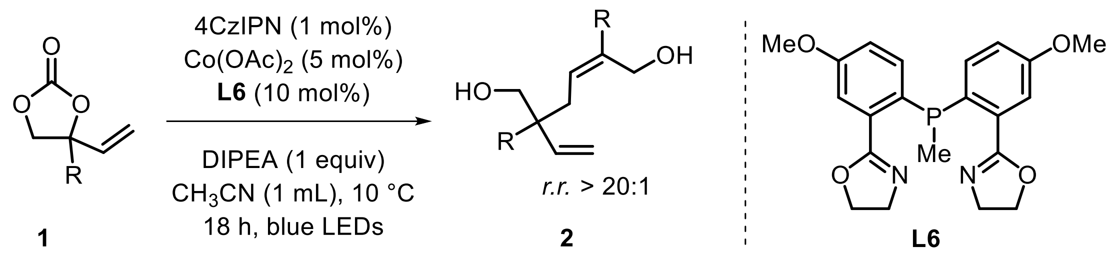

A 10 mL dram vial, stored under air, equipped with a magnetic stir bar was charged with 4CzIPN (1.6 mg, 2.0 µmol, 1.0 mol%), $Co(OAc)_2$ (1.8 mg, 10 µmol, 5 mol%) and **L6** (8 mg, 20 µmol, 10 mol%) and closed with a screw cap containing a silicone septum. After 3 vacuum/Ar cycles, MeCN (1 mL, 0.4 M based on VCC), VCC (**1**, 0.40 mmol, 2.0 equiv) and DIPEA (34.8 µL, 0.200 mmol, 1.0 equiv) were added in this order. The top of the reaction vial wrapped with Parafilm® and irradiated for 18 h at 10 °C[2] using a single high-power blue LED (λ_{em} = 439-457 nm, current: I = 700 mA) from the bottom. At this time, the reaction mixture was removed from the photoreactor and filtered through silica with EtOAc (15 mL). The filtrate was concentrated in vacuo. The crude was purified by column chromatography (SiO_2, eluent hexane:EtOAc, 7:3 isocratic) unless specified otherwise affording the product **2** (all compounds were purified using these conditions except **2j**).

(2) Note that the reaction mixture itself is warmer than 10 °C upon exiting the photoreactor.

Structural determination: NOESY of product ***2a***

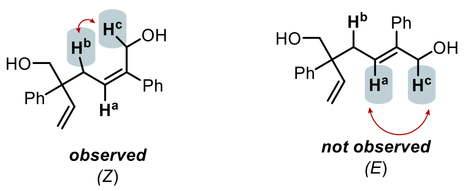

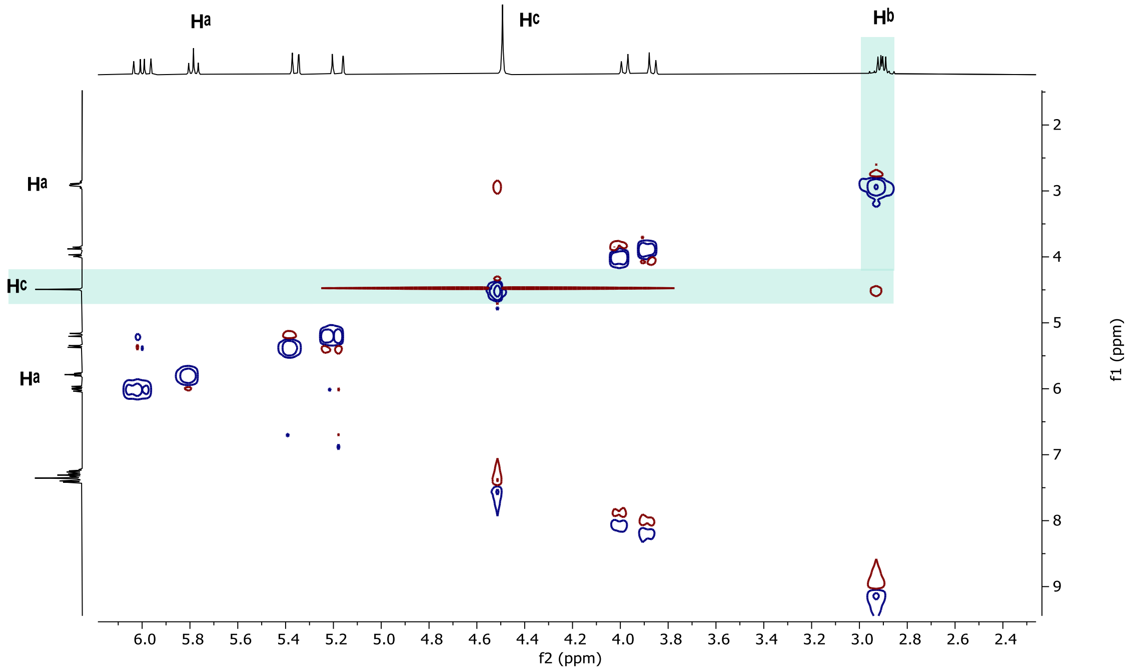

Experimental procedure: Homocoupling of **1a** with **L4**

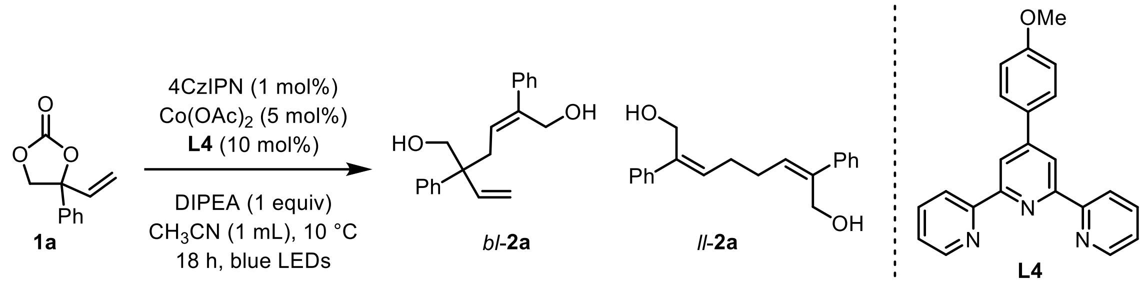

A 10 mL dram vial, stored under air, equipped with a magnetic stir bar was charged with 4CzIPN (1.6 mg, 2.0 µmol, 1.0 mol%), $Co(OAc)_2$ (1.8 mg, 10 µmol, 10 mol%) and **L4** (6.8 mg, 20 µmol, 20 mol%) and closed with a screw cap containing a silicone septum. After 3 vacuum/Ar cycles, MeCN (1 mL, 0.2 M based on VCC), VCC (**1a**, 38 mg, 0.20 mmol, 2.0 equiv) and DIPEA (17.5 µL, 0.100 mmol, 1.0 equiv) were added in this order. The top of the reaction vial wrapped with Parafilm® and irradiated for 18 h at 20 °C using a single high-power blue LED (λ_{em} = 439-457 nm, current: I = 700 mA) from the bottom. At this time, the reaction mixture was removed from the photoreactor and filtered through silica with EtOAc (15 mL). The filtrate was concentrated in vacuo. ^{1}H NMR of the crude with mesitylene (13.9 µL, 0.100 mmol, 1.0 equiv) as an internal standard showed the formation of **2a** with a *bl*:*ll* ratio of 1:2. The crude was then purified by column chromatography (SiO_2, hexane:EtOAc, 7:3 isocratic) affording *ll*-**2a** as a yellowish oil (15 mg, 0.050 mmol, 50%).

Experimental procedure: Homocoupling of 6-VCC **9a** with **L4**

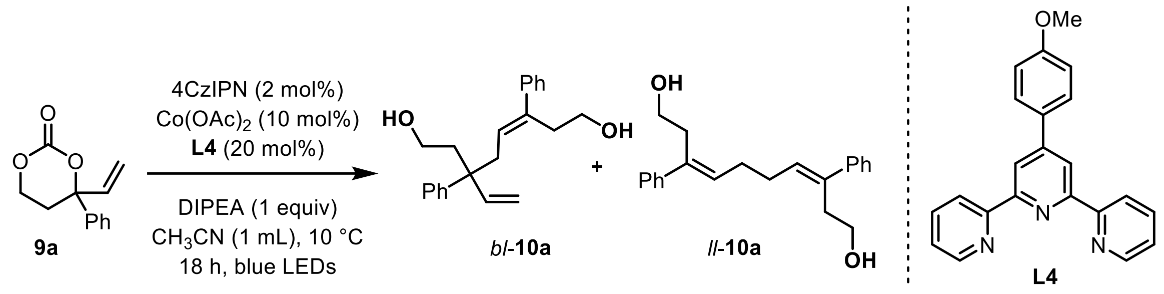

A 10 mL dram vial, stored under air, equipped with a magnetic stir bar was charged with 4CzIPN (1.6 mg, 2.0 μmol, 2.0 mol%), $Co(OAc)_2$ (1.8 mg, 10 μmol, 10 mol%) and **L4** (6.8 mg, 20 μmol, 20 mol%) and closed with a screw cap containing a silicone septum. After 3 vacuum/Ar cycles, CH_3CN (1 mL, 0.2 M based on VCC), 6-VCC (**9a**, 40.8 mg, 0.20 mmol, 2.0 equiv) and DIPEA (17.4 μL, 0.100 mmol, 1.0 equiv) were added in this order. The top of the reaction vial wrapped with Parafilm® and irradiated for 18 h at 20 °C using a single high-power blue LED (λ_{em} = 439-457 nm, current: I = 700 mA) from the bottom. At this time, the reaction mixture was removed from the photoreactor and filtered through silica with EtOAc (15 mL). The filtrate was concentrated in vacuo. ^{1}H NMR of the crude with 1 equiv of mesitylene (13.9 μL, 0.100 mmol, 1.0 equiv) as an internal standard, showed the formation of **10a** with a *bl*:*ll* ratio of 1:2.5. The crude was then purified by column chromatography (SiO_2, hexane:EtOAc, 7:3 isocratic) affording *bl*-**10a** as a yellowish oil (9 mg, 0.03 mmol, 30%) and *ll*-**10a** (21 mg, 0.071 mmol, 71%).

General procedure (**GP2**) for ligand synthesis (**L5** and **L6**)

Aryl bromide (SI-4) synthesis

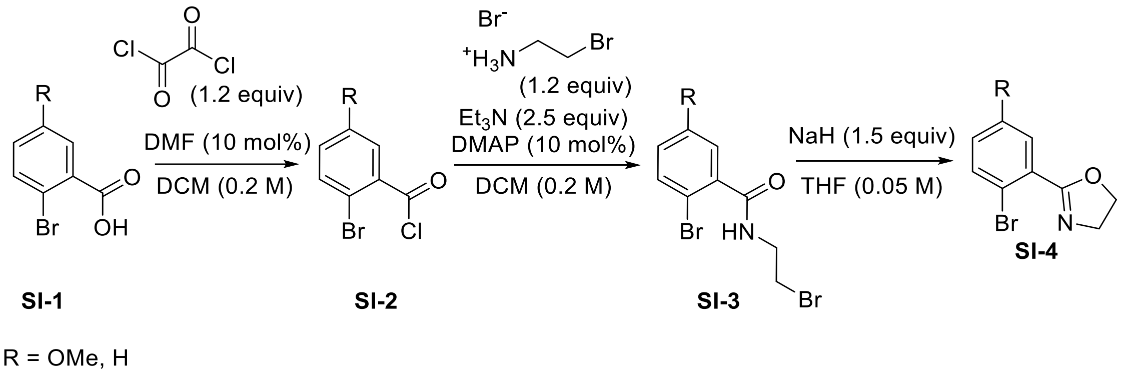

Step 1. Following a modified reported procedure,[3] an oven-dried round bottomed flask equipped with a magnetic stir bar was charged with 2-bromo-5-methoxybenzoic acid (**SI-1**, 7.00 g, 30.3 mmol). Under inert atmosphere, CH_2Cl_2 (anhydrous, 150 mL, 0.2 M) was added, and the solution was cooled to 0 °C with an ice bath. Oxalyl chloride (3.45 mL, 39.4 mmol, 1.3 equiv) was added, followed by DMF (235 µL, 3.03 mmol, 10 mol%). The reaction was stirred for 10 min at 0°C then r.t. for 5 h. The reaction mixture was concentrated in vacuo delivering the corresponding benzoyl chloride (**SI-2**) as a light-yellowish liquid. The crude product was directly used in the next step.

Step 2. Following an adapted reported procedure,[4] an oven-dried flask equipped with a magnetic stir bar was charged with 2-bromoethanamine hydrobromide (7.45 g, 36.4 mmol, 1.2 equiv) and DMAP (0.370 g, 72.7 mmol, 0.10 equiv). After 3 vacuum/Ar refilling cycles, CH_2Cl_2 (anhydrous, 95 mL) was added resulting in a colorless suspension. Triethylamine (10 mL, 73 mmol, 2.4 equiv) was then added and the suspension was cooled to -10 °C (salt/ice bath) while stirring. Then, a solution of the crude benzoyl chloride (**SI-2**) from step 1 was dissolved in CH_2Cl_2 (30 mL, total reaction molarity: 0.2 M based on the benzoyl chloride) and added dropwise (caution: on this scale the reaction temperature can rise causing an overpressure due to CH_2Cl_2 vapors). The reaction

(3) Ghorai, S.; Ur Rehman, S.; Xu, W.-B.; Huang, W.-Y.; Li, C. *Org. Lett.* **2020**, *22*, 3519–3523. https://doi.org/10.1021/acs.orglett.0c00962

(4) Rousseaux, S.; Gorelsky, S. I.; Chung, B. K. W.; Fagnou, K. Investigation of the Mechanism of C(Sp3)−H Bond Cleavage in Pd(0)-Catalyzed Intramolecular Alkane Arylation Adjacent to Amides and Sulfonamides. *J. Am. Chem. Soc.* **2010**, *132*, 10692–10705. https://doi.org/10.1021/ja103081n

mixture was left to warm slowly to room temperature and left for 18 h. The product was then washed with saturated aqueous $NaHCO_3$ (3 × 40 mL), aqueous HCl (1 M, 3 × 40 mL), and finally brine (40 mL). Separation of the organic layer and concentration in vacuo afforded a crude light-yellow solid product identified as 2-bromoethylamide (**SI-3**). The crude was used directly in the next step.

Step 3. Inspired from previous experience in *N*-alkylation procedures, an oven-dried flask equipped with a magnetic stir bar was charged with the crude 2-bromoethylamide (**SI-3**) and THF (600 mL, 0.05 M). Under argon, the solution was cooled to 0 °C and NaH (60% mineral oil dispersion, 1.94 g, 1.6 equiv) was added portion-wise (ca. 0.2 g at a time). The reaction mixture was stirred at r.t. for 3 h. Then it was quenched with water, extracted with EtOAc, and concentrated in vacuo before purification:

2-(2-bromo-5-methoxyphenyl)-4,5-dihydrooxazole was recrystallized from hexane:EtOAc (5:1, ca. 25 ml/g) afforded (**SI-4**, 4.33 g, 16.9 mmol, 56% yield over three steps) as a white crystalline solid.

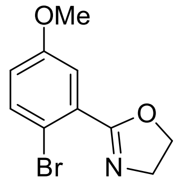

SI-4a
2.04 g
(50% over 3 steps)

^{1}H NMR (500 MHz, $CDCl_3$) δ 7.51 (d, *J* = 8.9 Hz, 1H), 7.25 (d, *J* = 3.1 Hz, 1H), 6.84 (dd, *J* = 8.8, 3.1 Hz, 1H), 4.45 (t, *J* = 9.6 Hz, 1H), 4.11 (t, *J* = 9.8 Hz, 2H), 3.80 (s, 3H). The compound was used with no further analysis.

2-(2-bromophenyl)-4,5-dihydrooxazole was purified via column chromatography (SiO2, hexane:EtOAc:Toluene 6:2:2) (**SI-4b**, 0.875 g, 3.87 mmol, 39% yield over three steps) as yellowish oil.

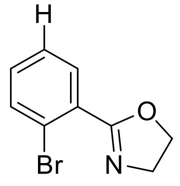

SI-4b
0.875 g
(39% over 3 steps)

^{1}H NMR (500 MHz, $CDCl_3$) δ 7.71 (dd, *J* = 7.7, 1.8 Hz, 1H), 7.64 (dd, *J* = 7.9, 1.3 Hz, 1H), 7.34 (td, *J* = 7.6, 1.3 Hz, 1H), 7.30 – 7.26 (m, 1H), 4.45 (t, *J* = 9.6 Hz, 2H), 4.11 (t, *J* = 9.6 Hz, 2H). The compound was used with no further analysis.

Lithiation-phosphorylation-methylation involving SI-4

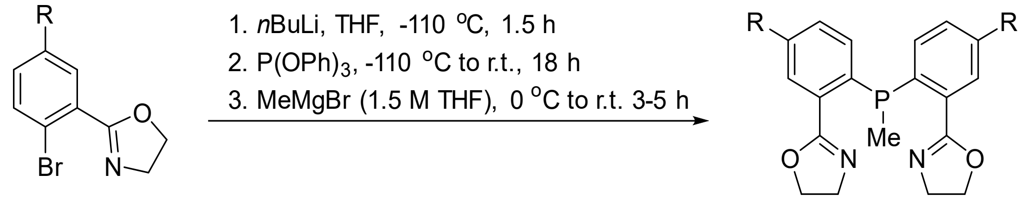

SI-4
R = OMe, H

L5, R = H
L6, R = OMe

Following a modified reported procedure, in an oven-dried Schlenk tube (50 mL, ext. Ø: 3 cm) equipped with a large stir bar (oval in shape, length 2 cm), the aryl bromide (**SI-4**, 1.08 g, 4.20 mmol, 2.1 equiv) was dissolved in THF (anhydrous, 15 mL) under an argon atmosphere and cooled down to –110 °C (Et_2O, N_2*(liq)*, bath temperature maintained between –100 and –110 °C and controlled with a thermometer).[6] Then, *n*-BuLi (1.6 M in hexane, 2.8 mL, 4.5 mmol, 2.25 equiv) was added dropwise and the mixture was stirred for 1.5 h at this temperature. Then, a solution of triphenylphosphite (524 µL, 2.00 mmol, 2.0 equiv) in THF (anhydrous, 2.0 mL, 1.0 M) was added in one portion under stirring. The reaction mixture stirred for 17 h with the cooling bath left to thaw and warm to r.t. (this results in evaporation of the Et_2O). At this time, the reaction is cooled to 0 °C (ice bath) and methyl magnesium bromide (3 M in THF, 1.0 mL, 3.0 mmol, 1.5 equiv) was added dropwise and stirred for 5 min at this temperature. The ice-bath was subsequently removed, and the reaction mixture was stirred for an additional 4 h at r.t. The mixture was quenched with 2 mL of degassed water[7] and filtered to remove the solids. The filtrate was dried over Na_2SO_4, filtered and concentrated in vacuo under a nitrogen atmosphere (purge prior to use and refilling with N_2).[8]

---

(5) Ghorai, S.; Ur Rehman, S.; Xu, W.-B.; Huang, W.-Y.; Li, C. *Org. Lett.* **2020**, *22*, 3519–3523. https://doi.org/10.1021/acs.orglett.0c00962

(6) We are fully aware that the THF freezes at -108 °C. Additionally, the Et_2O/N_2 bath is tricky, it seems to become increasingly difficult to control the temperature as time goes on, we speculate this could be due to condensation of water. We recommend consistent stirring which is subject to the equipment. In our case: with a well-centered on the stirring plate Schlenk tube with 400-500 rpm was optimal for this scale, glassware, stir bar and dewar size. Occasionally, freezing was still observed: the freezing occurs early in the lithiation step (first 30 min) and lasts more than 5 min (removing the flask and promoting agitation at r.t.): this has a negative effect on final yield (often only protodemetalation is observed). the freezing is observed after the first 30 min and last maximum 15 min: no observable effect on yield was observed.

(7) Deionized water was degassed by bubbling Argon though 10 mL for 30 min in an ultrasound bath, see *General Information* for further details.

(8) NB, oxidation was observed when the crude product and the product were left as a thin film open to the atmosphere.

Optimized purification: The crude product was purified by flash column chromatography (SiO_2, pure CH_2Cl_2 to CH_2Cl_2:MeOH 98:2 v/v to CH_2Cl_2:MeOH 97:3 v/v) and compressed nitrogen was used to limit the introduction of oxygen into the system. The pure fractions were concentrated in vacuo *under a nitrogen atmosphere* delivering a thin film that would then crystallize with sonication and if necessary, addition of a small quantity of Et_2O (less than 1 mL).

L6 (R = OMe) was afforded as an off-white amorphous solid (266 mg, 0.667 mmol, 33% yield).

L5 (R = H) was synthesized using the procedure above on 1.00 mmol scale obtained as a yellowish oil (160 mg, 0.473 mmol, 47% yield). We suspected that the oil nature of **L5** would make it highly prone to oxidation. We therefore set up the reactions (cf. 5. Mechanistic discussion - Electronic effects of the ligand) immediately after isolation where the phosphine oxide was not present (^{1}H and ^{31}P NMR). Our ^{13}C NMR analysis was submitted 72 hours after isolation and contains 10% phosphine oxide, confirmed by ^{31}P NMR.

Alternative purification of **L6**: Purification of the crude was carried out with neutral Al_2O_3 hexane:EtOAc (8:2 v/v), and after concentration to a thin film ether was added for crystallization. **L6** was obtained as an off white solid (234 mg, 0.587 mmol, 29% yield). This purification was implemented for the optimization studies and part of the scope of the transformation, however, often lead to lower yields due to the poor solubility of the ligand in hexane:EtOAc, with hexane not being exchangeable for pentane.

General procedure 2 (**GP3**): Vinyl cyclic carbonate synthesis

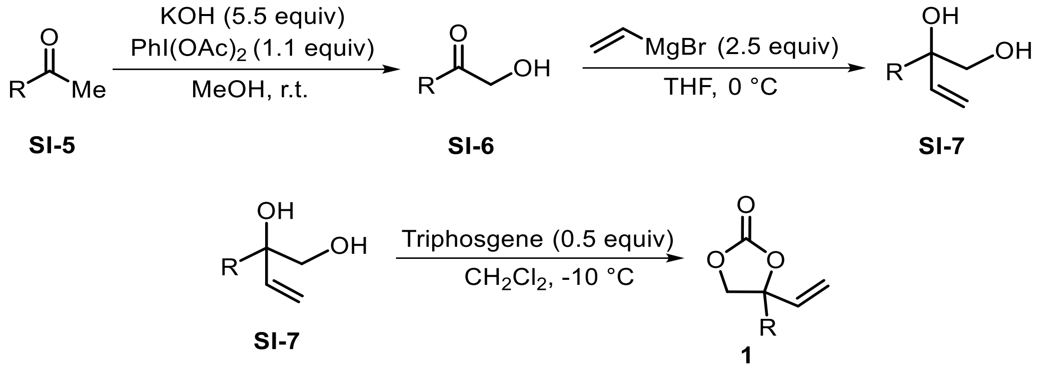

Substituted vinyl cyclic carbonates **1** were prepared according to a previously reported procedure.[9] All VCCs, except **1m** and **1s**, were previously characterized in literature.

Step 1: To a cold solution of the ketone **SI-5** (15 mmol, 1 equiv) in MeOH was added KOH (4.63 g, 82.5 mmol, 5.5 equiv) in portions, and then $PhI(OAc)_2$ (diacetoxyiodobenzene, 5.31 g, 16.5 mmol, 1.1 equiv) slowly. After being stirred at 0 °C–r.t. for 2-3 h, the reaction mixture was concentrated under reduced pressure, and the residue was washed with H_2O (30 mL) and extracted with Et_2O (30 mL). The organic phase was separated, dried over Na_2SO_4, and filtered. After concentration in vacuo, the obtained residue was dissolved in MeOH (30 ml) and treated with 2 N HCl (30 mL), after which the mixture was stirred for 18 h at r.t. Next, a saturated aqueous solution of $NaHCO_3$ was added to the reaction mixture until pH 7 and it was further diluted by CH_2Cl_2 (30 mL). The organic phase was separated and dried over anhydrous Na_2SO_4, filtered and concentrated under reduced pressure. The crude product was purified by silica gel chromatography (gradient: hexanes/EtOAc = 30:1 to 20:1 v/v) to yield **SI-6** typically in 60-70% yield.

(9) (a) Batt, F.; Fache, F. *Eur. J. Org. Chem.* **2011**, 6039–6055; (b) Hu, H.; Xu, W.-H.; Kang, W.-X.; Sun, W.; Sun, R.; Wei, X.-H.; Sun, M. *Org. Chem. Front.* **2021**, *8*, 4459–4465; (c) Roblin, A.; Casaretto, N.; Archambeau, A. *Org. Lett.* **2023**, *25*, 6453−6458; (d) Shi, L.; He, Y.; Chang, Y.; Zheng, N.; Yang, Z.; Gong, J. *Org. Lett.* **2019**, *21*, 3077−3080; (e) Guo, K.; Zeng, Q.; Villar-Yanez, A.; Bo, C.; Kleij, A. W. *Org. Lett.* **2022**, *24*, 637−641; (f) Dai, L.; Liu, W.; Zhou, Y. Q.; Zeng, Z.; Hu, X. Y.; Cao, W. D.; Feng, X. M. *Angew. Chem. Int. Ed.* **2021**, *60*, 26599−26603.

Step 2: An oven-dried Schlenk tube containing a magnetic stirring bar charged with **SI-6** (10 mmol, 1.0 equiv) was evacuated and back-filled with N_2, and then THF (20 mL) was added followed by a Grignard reagent (1.0 M in THF, 25 mL, 2.5 equiv) through a syringe at 0 ºC. After being stirred at room temperature for 4-5 h, the reaction mixture was quenched with a saturated aqueous $NaHCO_3$ and extracted with ethyl acetate (3 × 15 mL). The combined organic phases were dried over Na_2SO_4, concentrated under reduced pressure, and then the obtained residue was purified by column chromatography on silica gel (gradient: hexanes/EtOAc = 10:1 to 5:1 v/v) to afford the pure product (**SI-7**) typically in 50-70% yield.

Step 3: An oven-dried Schlenk tube containing a magnetic stirring bar charged with the diol intermediate **SI-7** (7 mmol, 1.0 equiv) was evacuated and back-filled with N_2. Then, pyridine (2.22 g, 28 mmol, 4 equiv) and a solution of triphosgene (1.04 g, 3.5 mmol, 0.5 equiv) in 10 mL CH_2Cl_2 were sequentially added by a syringe at –10 ºC. After being stirred at -10 ºC-to-r.t for 1-2 h, the reaction mixture was quenched with a saturated aqueous $NaHCO_3$ solution and diluted with CH_2Cl_2 (30 mL). The organic phase was washed with 1 N HCl (3 × 20 mL), $NaHCO_3$ (3 × 20 mL) and brine (3 × 20 mL). The combined organic phases were dried over Na_2SO_4, concentrated under reduced pressure, and then the obtained residue was purified by column chromatography on silica gel (gradient: hexanes/EtOAc = 15:1 to 10:1 v/v) to afford the pure product **1** as a light-yellow oil typically in 60-70% yield.

Scale-up of product **2a**

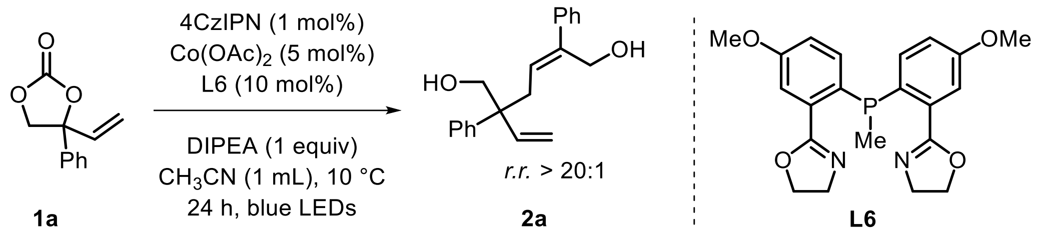

A 10 mL dram vial, stored under air, equipped with a magnetic stir bar was charged with 4CzIPN (7.9 mg, 10.0 μmol, 1.0 mol%), $Co(OAc)_2$ (8.9 mg, 50 μmol, 5.0 mol%) and **L6** (39.8 mg, 100 μmol, 10 mol%) and closed with a screw cap containing a silicone septum. After 3 vacuum/Ar cycles, MeCN (5.0 mL, 0.4 M based on VCC), VCC (**1a**, 380 mg, 2.00 mmol, 2.0 equiv) and DIPEA (174 μL, 1.00 mmol, 1.0 equiv) were added in this order. The top of the reaction vial was wrapped with Parafilm® and irradiated for 24 h at 10 °C using a single high-power blue LED (λ_{em} = 439-457 nm, current: *I* = 700 mA) from the bottom. At this time the reaction was removed from the photoreactor and filtered through silica with EtOAc (15 mL). The filtrate was concentrated in vacuo, conversion was determined by ^{1}H NMR using mesitylene (69.5 μL, 0.500 mmol, 0.50 equiv) as an internal standard. The crude mixture was then purified by column chromatography (SiO_2, hexane:EtOAc. isocratic 7:3 v/v), affording (*Z*)-2,5-diphenyl-5-vinylhex-2-ene-1,6-diol as a yellowish oil (**2a**, 171 mg, 581 μmol, 58%, 85% conv. 68% yield brsm).

Product modifications

Acylation

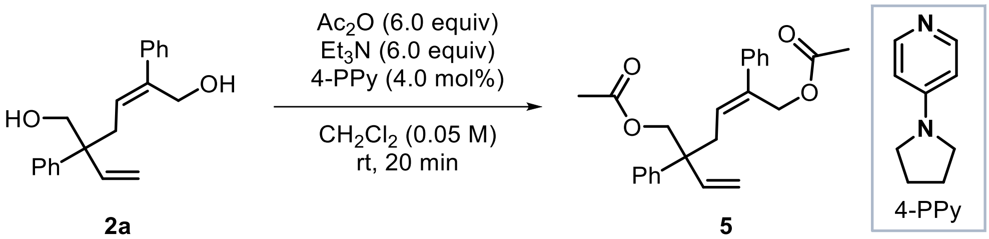

Following a reported procedure,[10] in a 50 mL round bottom flask equipped with a magnetic stir bar were added acetic anhydride (0.28 mL, 3.0 mmol, 6.0 equiv), triethylamine (0.42 mL, 3.0 mmol, 6.0 equiv) and 4-(pyrrolidin-1-yl)pyridine (3.0 mg, 20 µmol, 4.0 mol%). To this mixture was added a solution of diol **2a** (147 mg, 1.0 equiv, 0.50 mmol) in CH_2Cl_2 (10 mL, 0.05 M). The reaction mixture was stirred at r.t. upon reaction completion (checked via TLC, ca. 20 minutes) and the solution was concentrated in vacuo. The crude was purified by column chromatography (SiO_2, hexane:EtOAc, 9:1 v/v) affording the desired product as a yellowish oil (**5**, 370 mg, 490 µmol, 98% yield).

(10) Di Giacomo, B.; Coletta, D.; Natalini, B.; Ni, M.-H.; Pellicciari, R. *Il Farm.* **1999**, *54*, 600–610. https://doi.org/10.1016/S0014-827X(99)00070-1.

Epoxidation

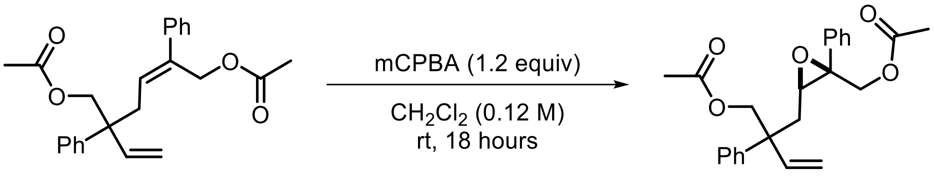

5 **6**

A 10 mL dram vial equipped with a magnetic stir bar was charged with diacetylated **5** (46.7 mg, 123 µmol, 1.0 equiv) and dry CH_2Cl_2 (1 mL, 0.12 M). *m*-CPBA (36.5 mg, 70 % wt., 0.15 mmol, 1.2 equiv) was added and the reaction was stirred at r.t. for 18 h. The solution was concentrated in vacuo, and the crude was purified by column chromatography (SiO_2, hexane:EtOAc, 85:15 v/v) obtaining the desired product as a yellowish oil (**6**, 20 mg, 90 wt% purity, 46 µmol, 37% yield).

Pd catalyzed amination

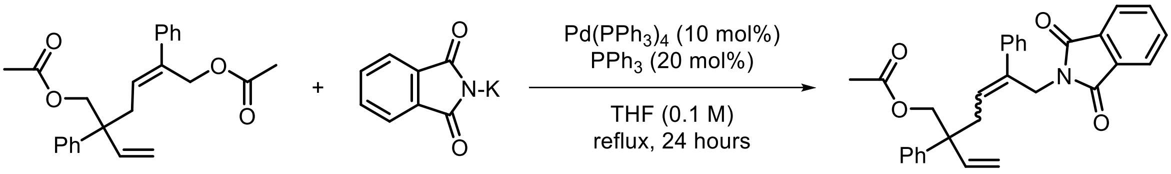

5 **SI-8** **7**

Following a slightly modified reported procedure,[11] to a 25 mL round bottom flask equipped with a magnetic stir bar and a reflux condenser were added diacetylated **5** (130 mg, 343 µmol, 1.00 equiv), potassium phthalimide (**SI-8**, 127 mg, 687 µmol, 2.00 equiv) and triphenylphosphine (18.0 mg, 68.7 µmol, 0.20 equiv). Following 3 vacuum/argon cycles, dry THF (3.5 mL, 0.10 M) was added. The suspension was degassed for 10 minutes by sparging argon in it and then $Pd(PPh_3)_4$ (39.7 mg, 34.3 µmol, 0.10 equiv) was added. The reaction vessel was sparged with argon gas, and the reaction mixture was allowed to stir at reflux (70 °C, oil bath) under an argon atmosphere for 24 h. The solution was concentrated in vacuo, and the crude was purified by column chromatography (SiO_2,

(11) Komanduri, V.; Krische, M. J. *J. Am. Chem. Soc.* **2006**, *128*, 16448–16449. https://doi.org/10.1021/ja0673027.

hexane:EtOAc, 9:1 v/v) obtaining the desired product as a colorless, amorphous solid (**7**, 101 mg, 217 μmol, 63% yield).

Structural determination

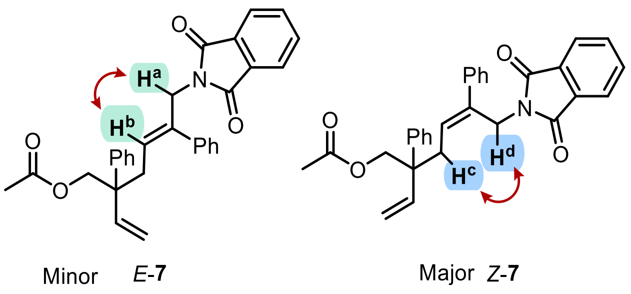

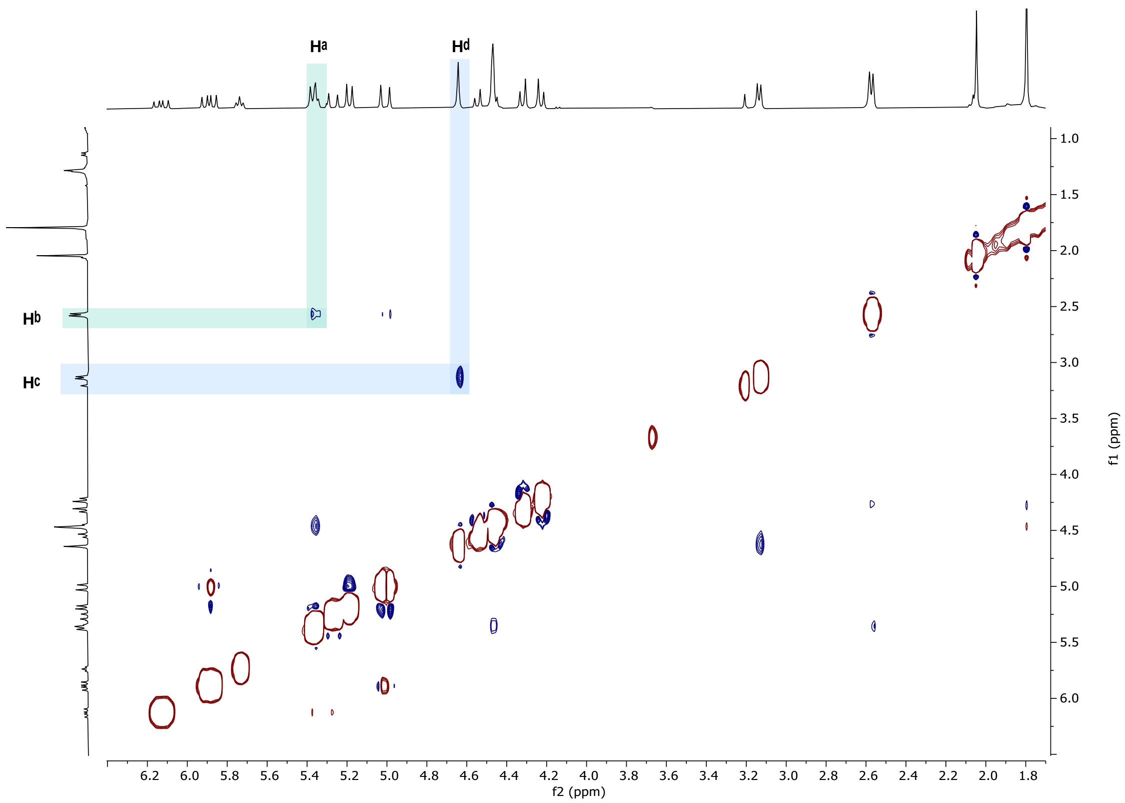

Alcohol oxidation

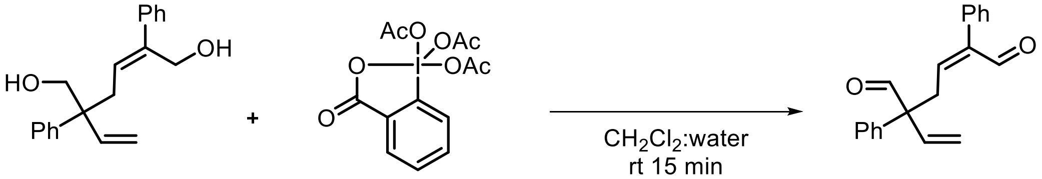

1a **DMP** *Z*-**8**

Following a slightly modified reported procedure,[12] in a 10 mL vial, "wet" CH_2Cl_2 was prepared by solvating H_2O (2 µL, deionized) in CH_2Cl_2 (2 mL, anhydrous) by drawing and expelling the biphasic mixture by means of a disposable glass Pasteur pipette (ca. 10 times). An oven dried round bottomed flask equipped with a magnetic stir bar and an isocratic dropping funnel was charged with diol **1a** (29.4 mg, 0.100 mmol, 1.0 equiv) and DMP (110 mg, 0.260 mmol, 2.6 equiv), followed by the addition of CH_2Cl_2 (0.6 mL, anhydrous). The "wet" CH_2Cl_2 solution was added dropwise via the dropping funnel over ca. 5 min. The solution was stirred for an additional 15 min then diluted with Et_2O (15 mL) resulting in a cloudy suspension. The latter was then concentrated in vacuo until 0.5 mL was left. The latter was further diluted with 30 mL of Et_2O then filtered through a celite plug. The filtrate was concentrated in vacuo to afford the crude dialdehyde (*Z*)-**8** (d.r. = 13:7, 28 mg, 0.96 mmol, 96% yield) as a yellowish oil with no need for further purification.

(12) Meyer, S. D.; Schreiber, S. L. *J. Org. Chem.* **1994**, *59*, 7549–7552. https://doi.org/10.1021/jo00103a067.

Alkene isomerization

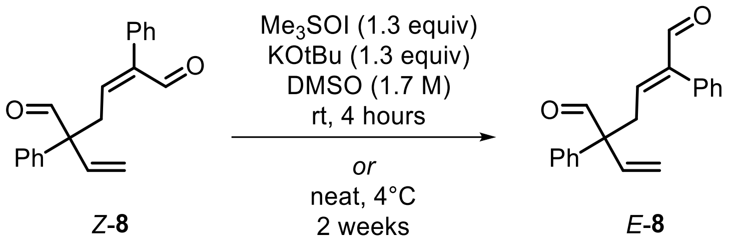

Z-**8** *E*-**8**

Following a modified procedure,[13] an oven-dried 25 mL flask equipped with a magnetic stir bar was charged with trimethylsulfoxonium iodide (28.6 mg, 130 µmol, 1.3 equiv) and potassium *tert*-butoxide (14.6 mg, 130 µmol, 1.3 equiv). Under an Ar atmosphere, DMSO (0.40 mL) was then added dropwise to the flask, the reaction mixture was stirred for 15 min, during which the solution became clear. (*Z*)-2,5-diphenyl-5-vinylhex-2-enedial (29.0 mg, 0.10 mmol, 1.0 equiv) in DMSO (0.20 mL) was added in one portion via syringe. The reaction mixture was allowed to stir for 4 h at room temperature, then quenched by addition of water (10 mL) and the reaction was freeze-dried (in-house set up using liquid N_2 and a Schlenk line with a gas trap protecting the Schlenk line). The resulting crude was solubilized in Et_2O, washed with brine dried over Na_2SO_4, and the volatiles were removed under reduced pressure to yield crude (*E*)-2,5-diphenyl-5-vinylhex-2-enedial, *E*-**8**, as a yellowish oil (17 mg, 59 µmol, 59 %). The compound was characterized as such with no further purification. We also observed the formation of the *E*-**8** after 2 weeks in the fridge, decomposition was observed after a prolonged period in chloroform.

(13) Hao, W.; Harenberg, J. H.; Wu, X.; MacMillan, S. N.; Lin, S. *J. Am. Chem. Soc.* **2018**, *140*, 3514–3517. https://doi.org/10.1021/jacs.7b13710.

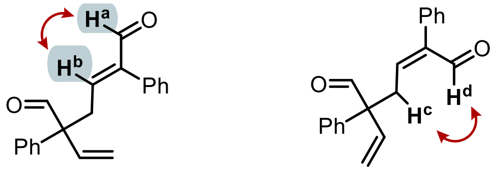

product of isomerisation

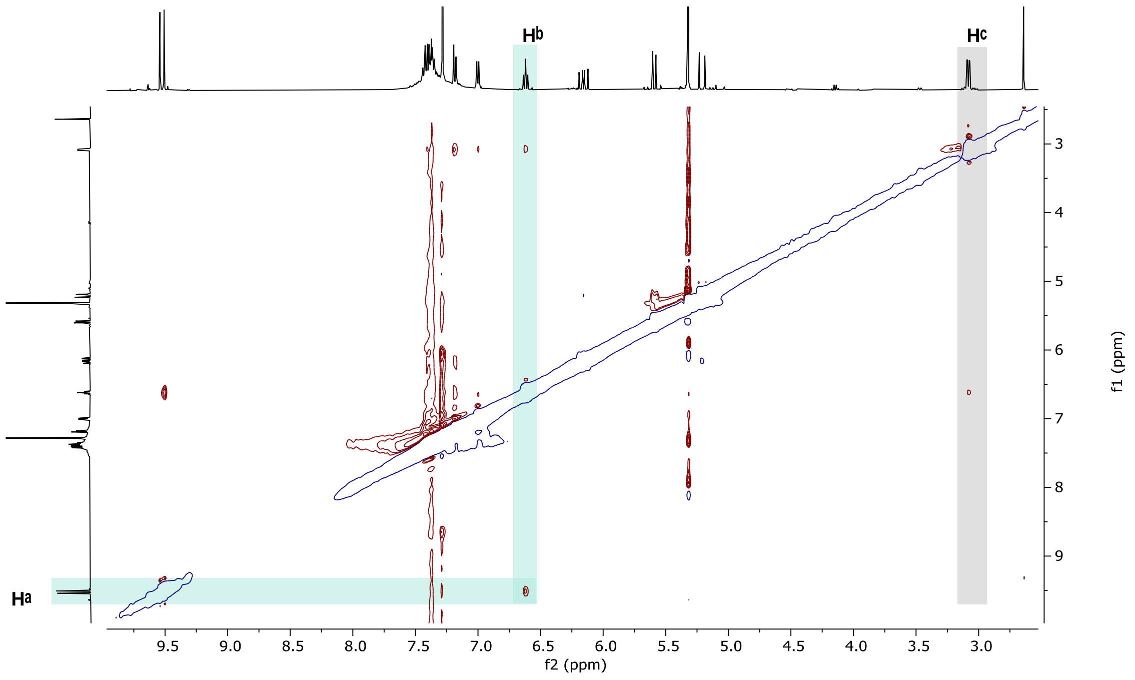

4. Optimization

General procedure (**GP4**) for screening:

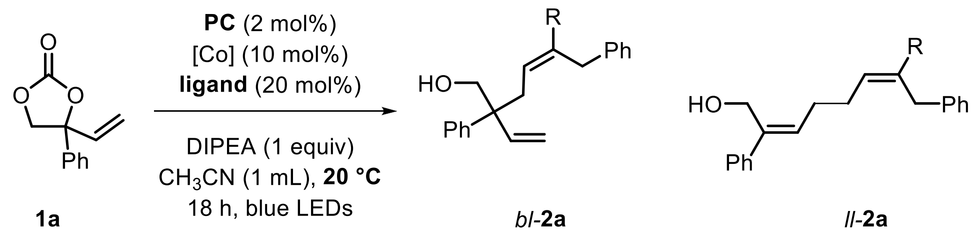

1a *bl*-**2a** *ll*-**2a**

A 10 mL dram vial, stored under air, equipped with magnetic stir bar was charged with **PC** (2 mol%), [Co] (10 mol% or otherwise specified) and ligand (20 mol% or otherwise specified). The reaction vial was sealed with a cap with a silicone septum. After 3 vacuum/Ar cycles, MeCN (1 mL, 0.2 M, anhydrous AcroSeal on 4 A MS - *not degassed*), VCC **1a** (38 mg, 0.20 mmol, 2.0 equiv) and DIPEA (17.4 µL, 0.100 mmol, 1.0 equiv) were added in this order under Ar. The reaction was further sealed with Parafilm® and irradiated for 18 h at 20 °C (unless elsewhere specified) using a single high-power blue LED (Royal blue, $\lambda_{em,max}$ = 445 nm, 700 mA) from the bottom. After completion, the mixture was directly filtered through silica eluting with EtOAc (15 mL). The filtrate was concentrated in vacuo, and the residue was dissolved in $CDCl_3$ and analyzed by ^1H NMR using mesitylene (13.9 µL, 0.100 mmol, 1.0 or 0.5 equiv) as an internal standard.

Crude ^{1}H NMR (400 MHz, $CDCl_3$) of Table 1 entry 1 (using **L1**)[14]

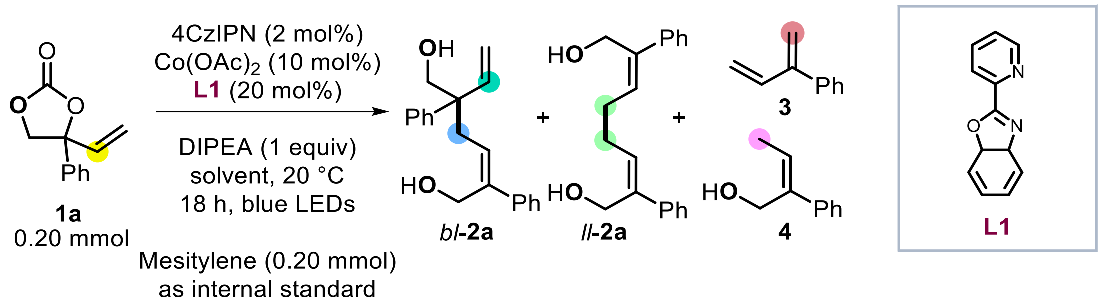

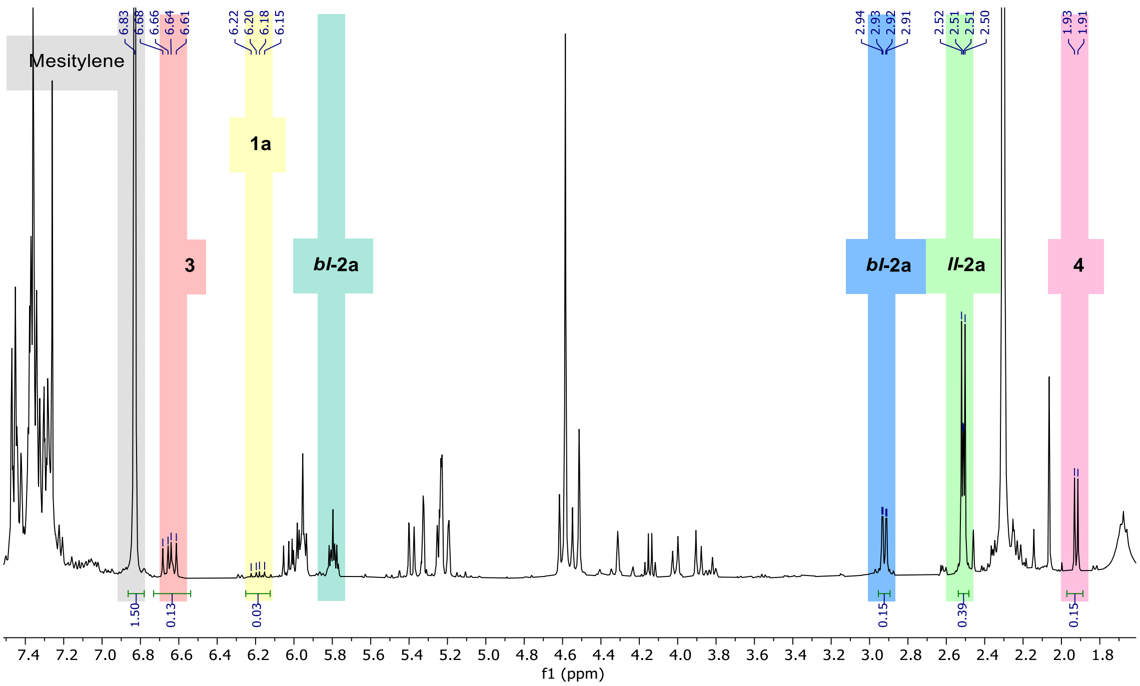

Figure S3. ^{1}H NMR analysis of the crude reaction mixtures accessing **2a**

(14) Compounds **3** and **4** are known, their characterization data can be found here: Xue, S.; Cristòfol, À.; Limburg, B.; Zeng, Q.; Kleij, A. W. *ACS Catal.* **2022**, *12*, 3651–3659. https://doi.org/10.1021/acscatal.2c00660. and Denmark, S. E.; Pan, W. *Org. Lett.* **2003**, *5*, 1119–1122. https://doi.org/10.1021/ol0342002.

Full ligand screening with product NMR yields (%)

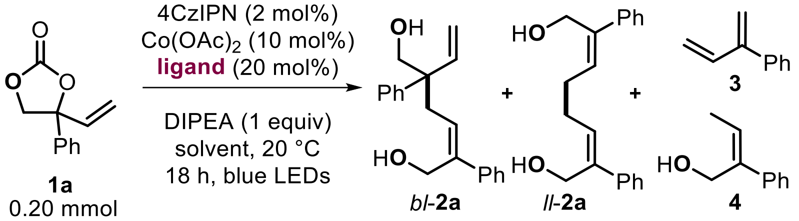

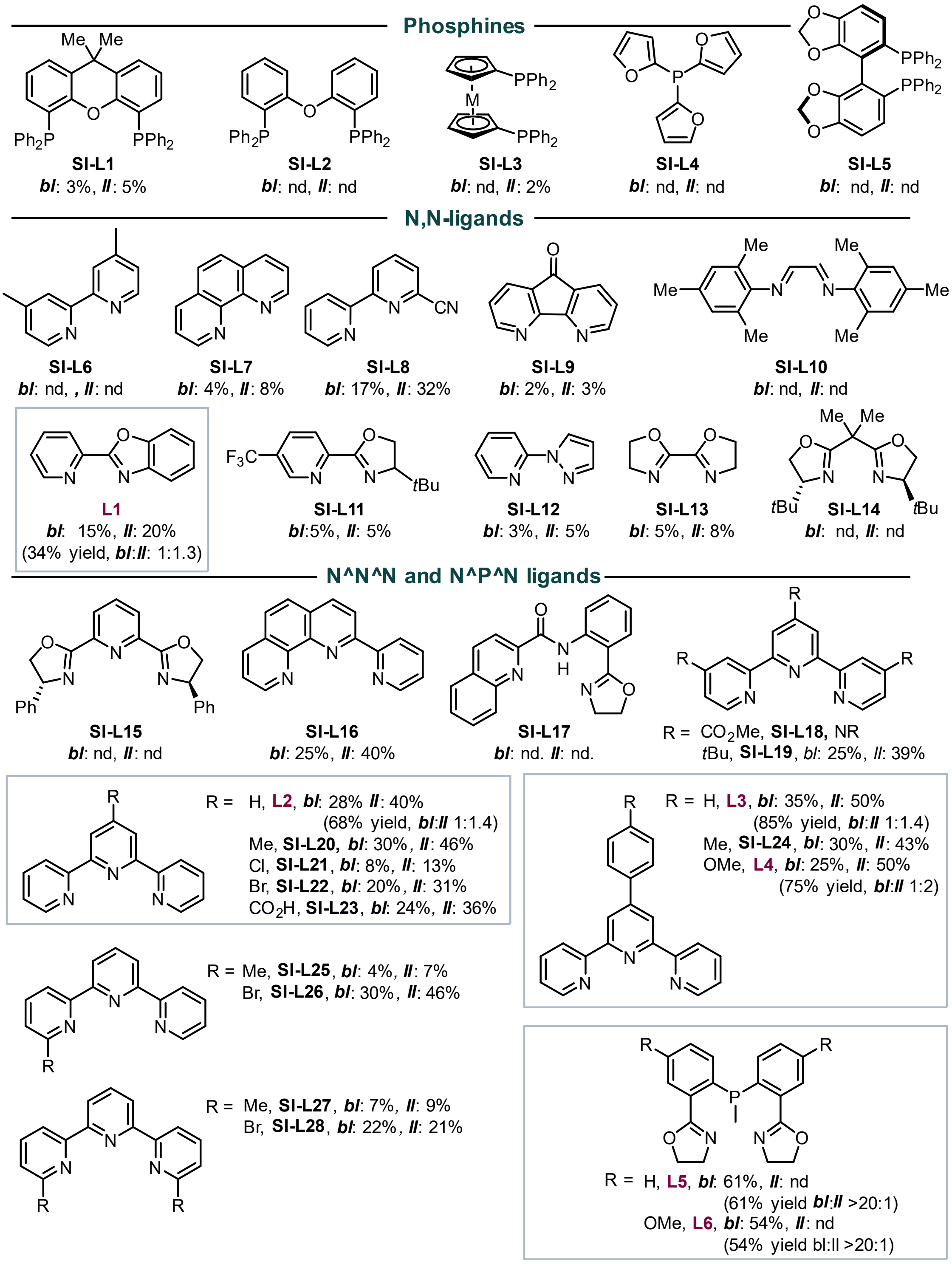

Screening with commercial ligand **L4**

Table S2. Additional optimization studies using **L4**[a]

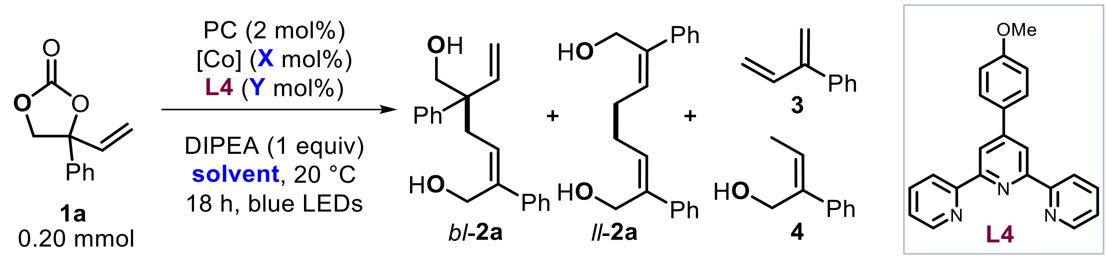

Entry	PC	[Co]	X:Y (mol%)	Solvent	Conv. (%)[a]	Yield (%)[a]	*bl*:*ll*[a]
**1**	4CzIPN	$Co(OAc)_2$	10:20	MeCN	Full	75	1:2
**2**	4CzIPN	$Co(OAc)_2$	10:20	DMA	Full	74	1:2
**3**	4CzIPN	$Co(OAc)_2$	10:20	DMF	Full	84	1:1.5
**4**	4CzIPN	$Co(OAc)_2$	10:20	TMA	Full	58	1:1.9
**5**	4CzIPN	$Co(OAc)_2$	10:20	MeCN + H_2O (10 eq)	Full	91	1:1.2
**5**	4CzIPN	$Co(OAc)_2$	10:10	DMA	Full	74	1:2
**6**	4CzIPN	$CoBr_2$	10:20	MeCN	40	31	1:2.8
**7**	4CzIPN	$Co(OTf)_2$	10:20	MeCN	–	–	–
**8**	4CzIPN	$Ni(glyme)Br_2$	10:20	DMA	–	–	–
**9**	4CzIPN	$Co(OAc)_2$	5:10	MeCN	Full	77	1:1.4
**10**	4CzIPN	$Co(OAc)_2$	1:2	MeCN	Full	77	1:1.4
**11**	$Ir(ppy)_3$	$Co(OAc)_2$	10:20	MeCN	< 20	n.d.	–
**12**	Rhodamine 6G	$Co(OAc)_2$	10:20	MeCN	< 10	n.d.	–

[a]Determined by ^{1}H NMR analysis using mesitylene as an internal standard according to **GP4**

Unsuccessful substrates

All substrates presented below were tested under the optimized conditions (Table 1, entry 8: main text):

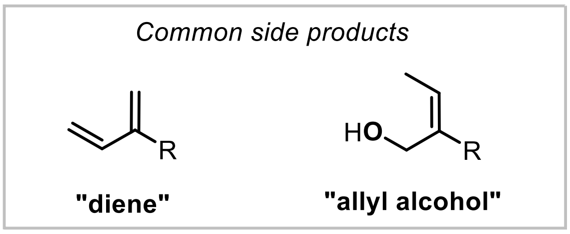

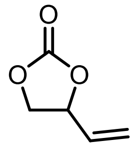

80% conv.
no product detected

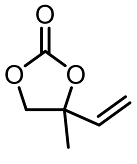

low conv.
no product detected

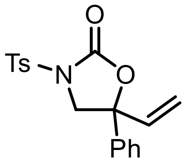

low regioselectivity
and yield <35%

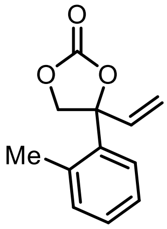

no conv.

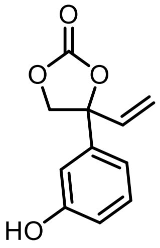

low conv.
trace of allyl alcohol

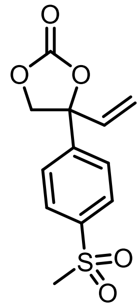

decomp.
21% of diene formation and
25% allyl alcohol

Optimization of **L6** synthesis

Table S3. Optimization of the lithiation-phosphorylation process.

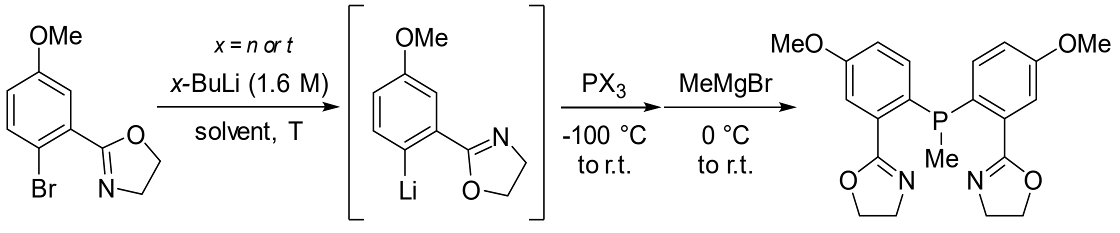

SI-4a **I** **L6**

Entry	Reagent modification	Lithiation (°C)a	$P(OPh)_3$ add. (°C)[a]	Conv. (%)[b]	Yield (%)[c]
1[d]	None	-78	-100	>99	8–40
2	*t*BuLi instead of *n*BuLi	-78	-100	>99	10–30
3	Dropwise addition of $P(OPh)_3$	-78	-100	>99	10
4	Reverse addition: **I** to a solution of $P(OPh)_3$	-78	-100	>99	n.d.
5	$PCl_2(NEt_2)$ instead of $P(OPh)_3$	-78	-100	>99	n.d.
6	PCl_2(*t*Bu) instead of $P(OPh)_3$ (no MeMgBr)	-78	-100	>99	n.d.
7	Et_2O instead of THF	-78	-100	>99	10–30
8	None	0	-100	>99[e]	n.d.
9	None	-100	-100	>99	15–30
**10**	**Dropwise $P(OPh)_3$ and vigorous stirring**	**-105**	**-105**	**>99**	**30–50**

[a]Determined externally by use of a cryogenic thermometer and known cooling bath combinations. [b]Determined by TLC and ^1H NMR. [c]Isolated yields, ranges represent the minimum and maximum yields obtained after 5 repetitions. [d]Reported conditions. [e]No debrominated product was detected by ^1H NMR, full decomposition of **SI-4a** was observed. n.d. stands for not determined.

5. Mechanistic discussion

Rationale for lower yields

We observed a correlation between the yield and the Hammett parameters (**Table S4** and **Figure S4**).[15] Although this is not a Hammett plot, we can see that for all highly polarized σ < - 0.2 and σ > + 0.3 our reaction yields drop.

Table S4. Hammet parameters and yield of homocoupling

Entry	VCC	Ph-substitution	σ_p	σ_m	Yield (%)
1	**1g**	*p*-OMe	- 0,268	-	22
2	**1c**	*p*-*t*Bu	- 0,197	-	76
3	**1b**	*p*-Me	- 0,17	-	70
4	**1k**	*m*-Me	-	- 0,069	67
5	**1a**	H	0	0	71
7	**1j**	*p*-Ph	+ 0,06	-	30
8	**1d**	*p*-F	+ 0,062	-	73
9	**1l**	*m*-OMe	+ 0,115	-	73
10	**1q**	β-napthyl	+ 0,17	-	67
11	**1e**	*p*-Cl	+ 0,227	-	59
12	**1f**	*p*-Br	+ 0,232	-	50
13	**1n**	*m*-CF_3	-	+ 0,43	26
14	**1i**	*p*-CF_3	+ 0,551	-	21

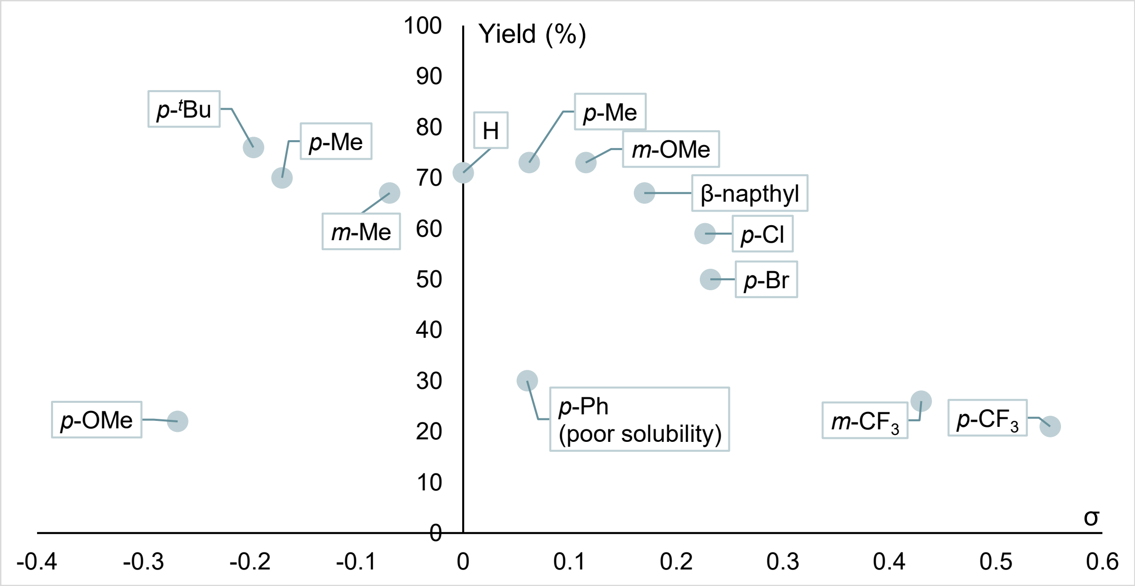

Figure S4. Graphical depiction of yield trends based on Hammett constants.

(15) (a) Hansch, Corwin.; Leo, A.; Taft, R. W. *Chem. Rev.* **1991**, *91*, 165–195. https://doi.org/10.1021/cr00002a004. (b) McDaniel, D. H.; Brown, H. C. *J. Org. Chem.* **1958**, *23*, 420–427. https://doi.org/10.1021/jo01097a026.

To gain a greater understanding of this correlation, we have compared crude ^{1}H NMR of three reactions (Figure S5). From the preoptimized stage, we can clearly see the formation of 4 products: conversion (> 95 %), ***bl*-2a** (15 %), ***ll*-2a** (20 %), **3** (diene, 11 %) and **4** (reduction product, < 5 %). With the optimized ligand **L6** and **1i,** we observe: conversion (75 %), ***bl*-2a** (24 %), ***ll*-2a** (< 5 %), **3** (diene, 10 %) and **4** (reduction product, 19 %) and **1g**: conversion (88 %), ***bl*-2a** (18 %), ***ll*-2a** (< 5 %), **3** (diene, 11 %) and **4** (reduction product, 7%). This illustrates that similar to when using **L1**, **L6** delivers more complex reaction mixtures with lower chemoselectivity: both the diene and reduction products are present and lower conversion.

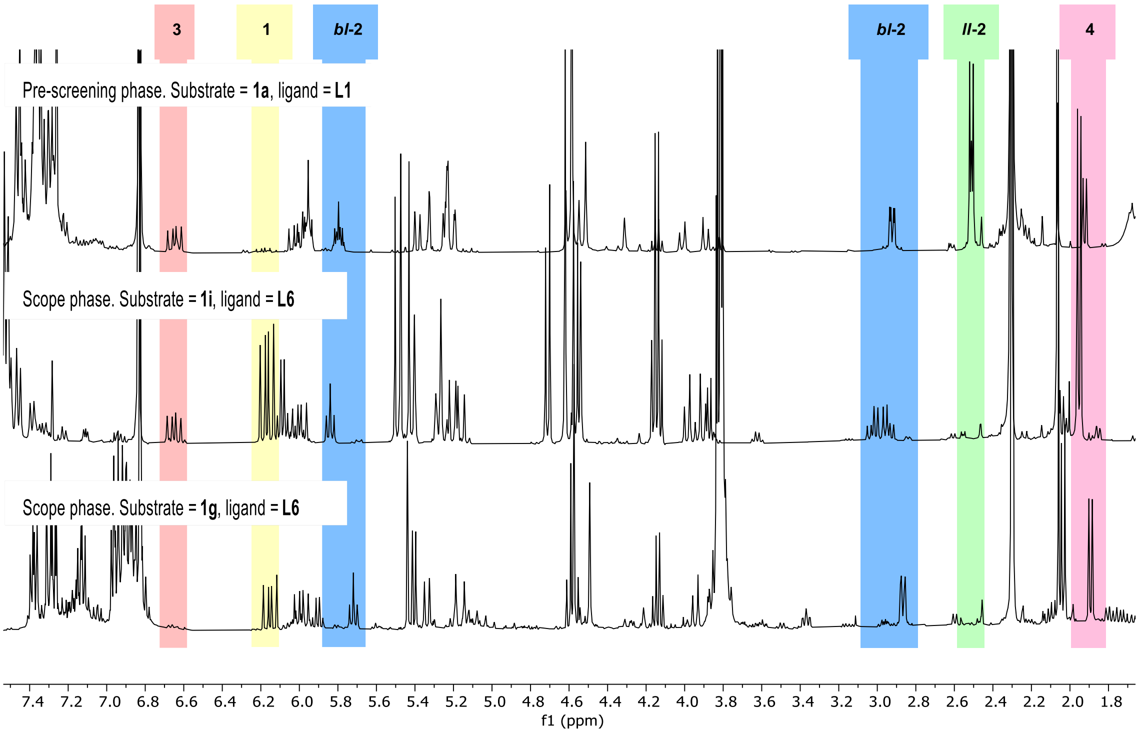

Figure S5. Stacked ^{1}H NMR analyses of the crude reaction mixtures accessing **2a** with a poorly suited ligand **L1** (top) and, **2i** (middle) and **2g** (bottom), with the optimized ligand **L6**

Electronic effects of the ligand

To push things further, we decided to compare the electronics of the ligand with an electron poor, rich and neutral substrate. We selected **1f** (p-Br derivative, σ_p = + 0.232), **1g** (p-OMe derivative, σ_p = - 0.268) and **1a** (p-H derivative, σ_p = 0).

The results in table S5 illustrate that **L5** and **L6** overall perform similarly. A slight increase in yield of **2g** is noted although this is also paired with higher conversion.

Table S5. Comparison between **L5** and **L6** with **1a**, **1f** and **1g**[a]

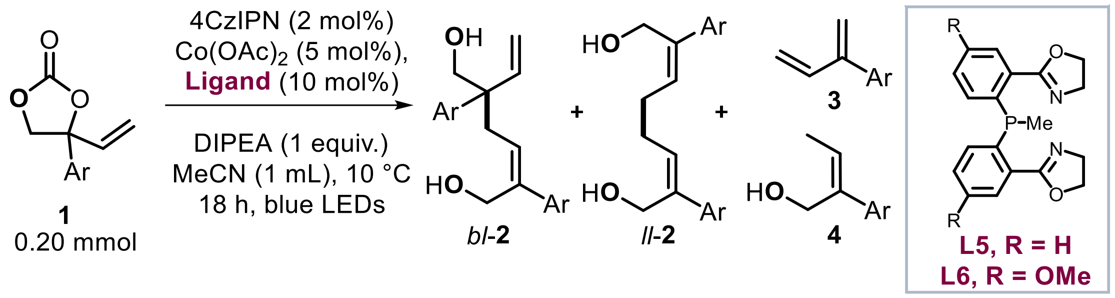

Entry	Substrate	Ligand	Conv. (%)[b]	Yield *bl*-2 (%)[b]	Comments
1	**1a**	**L5**	full	71	0.4 mmol VCC-
2	**1a**	**L6**	full	71	-
3	**1f**	**L5**	full	54	**4**, 7%
4	**1f**	**L6**	90%	52	**4,** 8%
5[c]	**1g**	**L5**	full	36	**4** impurity under the signal
6	**1g**	**L6**	88	18	**3**, 11 % and **4** 7%

Reactions were set-up following GP 1 [b]Determined by ^{1}H NMR analysis using Mesitylene as an internal standard according to **GP4**. [c]reaction performed on 0.123 mmol scale.

Control reactions

Table S6. Control reactions

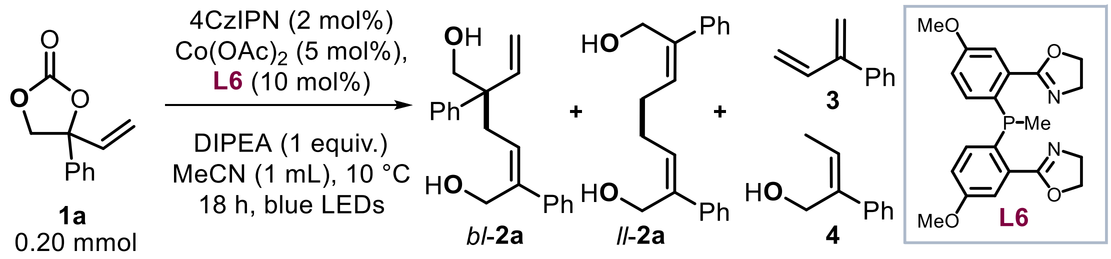

Entry	Modification to the standard conditions	Conv. (%)[a]	Yield (%)[a]
1	No PC (**L6**, 20 °C)	15	n.d.
2	No light (**L6**, 20 °C)	–	n.d.
3	No $Co(OAc)_2$, no ligand (20 °C)	–	n.d.
4	No ligand	30	n.d.[b]
5	Air, water (**L4**, 20 °C)	100	68
6	TEMPO (2.0 equiv)	–	n.d.
7	TEMPO (2.0 equiv no light)	–	n.d.
8	BHT (2.0 equiv)	Full	55%
9	DPE (2.0 equiv)	Full[c]	40%
10	Zn	–	n.d.
11	Et_3N	50	Traces
12	DIPEA 0.5 eq	85	47[d]
13	DIPEA 1 eq	–	71
14	DIPEA 2 eq	Full	60
15	No DIPEA (**L6**, 10 °C)	20	n.d.
16	No DIPEA (**L6**, 10 °C, 10 mol% of 4CzIPN)	20	n.d.
17	No DIPEA (**L4**, 10 °C)	-	n.d.
18	No DIPEA (**L4**, 10 °C, 10 mol% of 4CzIPN)	-	n.d.
19	No PC (**L4**, 20 °C)	-	n.d.

[a]Determined by ^1H NMR analysis using Mesitylene as an internal standard according to **GP4.** n.d. is not detected. [b]28% of **4** (reduction product). [c]75% residual DPE. [d]11% of *ll*-**2a**.

Homocoupling of a 6VCC using **L6**

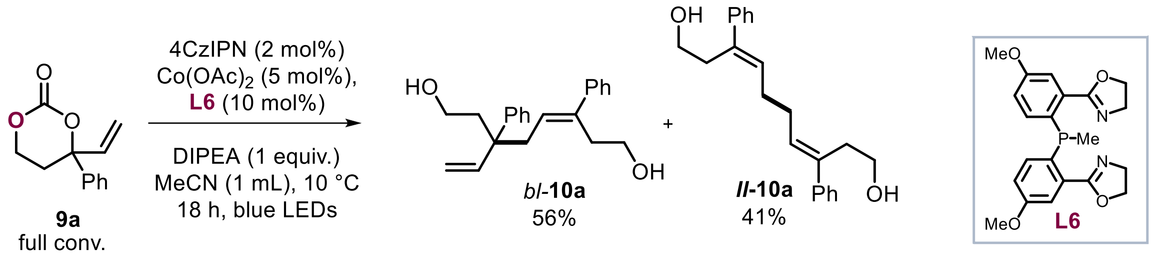

Following **GP4** using **9a** (40.8 mg, 0.20 mmol, 2.0 equiv). The ^{1}H NMR spectrum of the crude containing mesitylene (13.9 µL, 0.100 mmol, 1.0 equiv) as an internal standard showed the formation of *bl*-**10a** (56%, NMR yield) and *ll*-**10a** (41%, NMR yield).

Crossover experiment

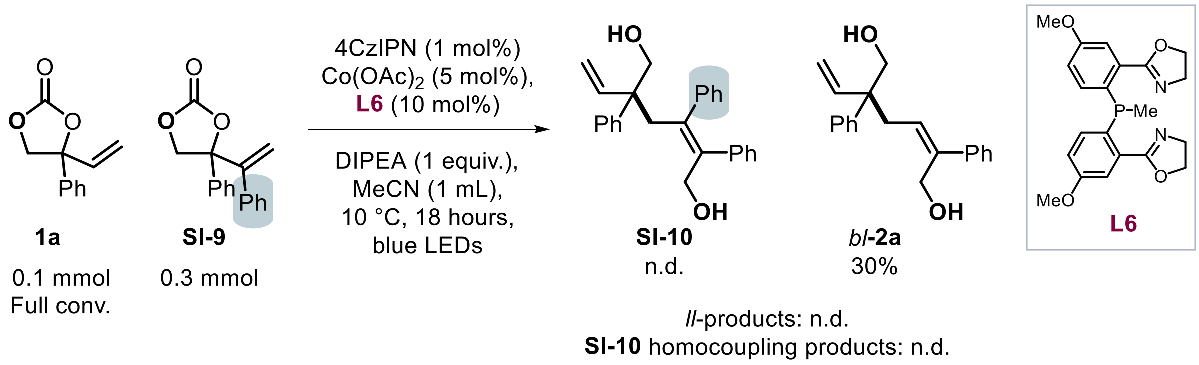

Following a modified **GP4** with **1a** (19 mg, 0.10 mmol, 1.0 equiv) and **SI-9** (80 mg, 0.30 mmol, 3.0 equiv) and DIPEA (17.4 µL, 0.100 mmol, 1.0 equiv). ^{1}H NMR of the crude with mesitylene (13.9 µL, 0.100 mmol, 1.0 equiv) as an internal standard showed the formation of *bl*-**2a** (30%, NMR yield) and 33% conversion of **SI-9**. Cross-allyl products (**SI-10** or other) were not detected, and products resulting from the homocoupling of **SI-10** were also not detected. This activated VCC (**SI-9**) mimics the DPE radical trap while maintaining the VCCs capacity to undergo decarboxylation. It has been reported to be a suitable reaction partner for nucleophilic radicals, yet here we observed no cross-coupling products further confirming our prior attempts that were focused on trapping an allyl radical with no success.

Complete mechanistic proposal

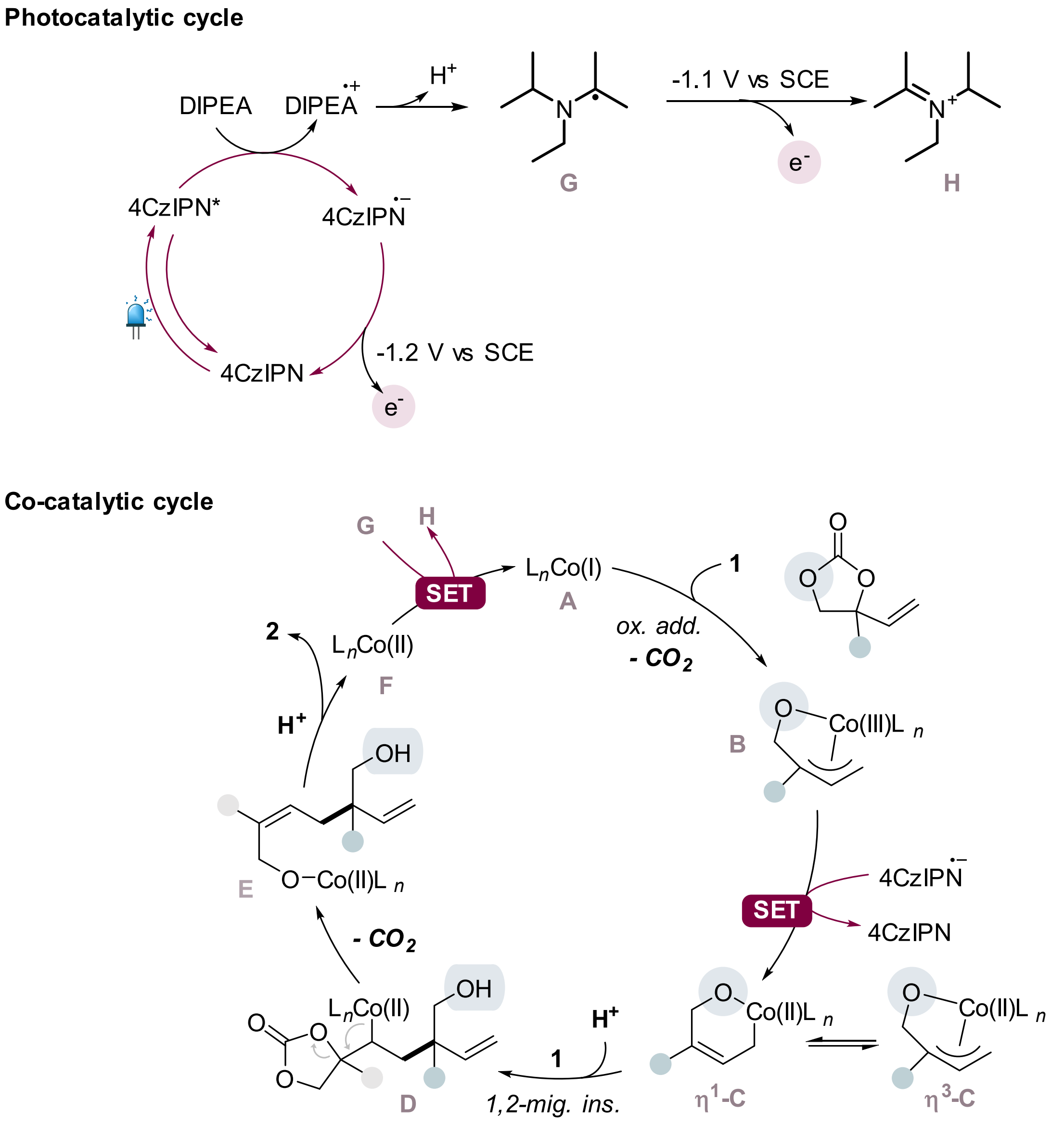

Scheme S1. Complete proposed catalytic system. Top: photocatalytic cycle and redox data. Bottom: cobalt-based catalytic cycle.

Under blue LED irradiation, the ground state 4CzIPN can undergo photoexcitation to its excited state 4CzIPN*. Based on our previous studies,[16] the TADF triplet 4CzIPN* is the active photocatalyst, and undergoes rapid oxidative quenching with DIPEA generating the DIPEA$^{\bullet+}$. Our control reactions in absence of DIPEA (both with the optimized 2 mol% and 10 mol% of 4CzIPN) illustrate that DIPEA is required to see product formation.

(16) (a) Limburg, B.; Cristòfol, À.; Kleij, A. W. *J. Am. Chem. Soc.* **2022,** *144*, 10912–10920. https://doi.org/10.1021/jacs.2c03692. (b) Xue, S.; Cristòfol, A.; Limburg, B.; Zeng, Q.; Kleij, A. W. *ACS Catal.* **2022**, *12*, 3651–3659. https://doi.org/10.1021/acscatal.2c00660

Although this does not exclude the possibility of 4CzIPN* reducing L_nCo(II) **F**[17] to our active L_nCo(I) intermediate **A**, we believe this is unlikely in presence of DIPEA.[18]

DIPEA$^{•+}$ being highly acidic can act as a complementary proton source to the water from undried glassware we use, allowing protonation of intermediates **C** or **E** and forming a transient neutral α-amino radical **G**. The latter has a similar $E_{1/2}$(Ox/Red)[19,20] to 4CzIPN$^{•-}$ [capable of reducing Co(III) to Co(II) and Co(II) to Co(I)]. Both 4CzIPN$^{•-}$ and **G** could act as single electron reductants in this transformation. Our control using Et_{3s}N (50% conv. trace product formation) demonstrates that Et_3N can indeed promote the formation of the key intermediates allowing the reaction to initiate but unlike DIPEA the process is less efficient leading to decomposition and potentially catalyst deactivation. We believe this can be explained by the structural differences between DIPEA and Et_3N. Where the oxidation/deprotonation of DIPEA delivers a tertiary α-amino radical with increased protective steric bulk, Et_3N delivers a sensibly less stable and less sterically protected secondary α-amino radical. We therefore speculate that radical **G** likely turns over the Co-catalytic cycle (**F** to **A**) and the 4CzIPN$^{•-}$ reduces **B** to **C**, further supporting our discussion in the manuscript.

---

(17) We postulate that this also corresponds to the speciation of Co(OAc)2 and **L6** in the initiation of the catalytic cycle

(18) This side reactivity is also observed in absence of the photocatalyst and could result from the fact that **L6** is a phosphine ligand prone to oxidation (the exact same controls run with terpyridine **L4** no conversion is observed cf. table **S6**).

(19) (a) Speckmeier, E.; Fischer, T. G.; Zeitler, K. *J. Am. Chem. Soc.* **2018**, *140*, 15353–15365. https://doi.org/10.1021/jacs.8b08933. (b) Wayner, D. D. M.; Dannenberg, J. J.; Griller, D. *Chem. Phys. Lett.* **1986**, *131*, 189–191. https://doi.org/10.1016/0009-2614(86)80542-5.

(20) Example of a reported to promote a reductive cyclization promoted by DIPEA radical **G**: Ismaili, H.; Pitre, S. P.; Scaiano, J. C. *Catal. Sci. Technol.* **2013**, *3*, 935–937. https://doi.org/10.1039/C3CY20759E.

6. Characterization data for all new compounds

2,2'-((methylphosphanediyl)bis(2,1-phenylene))bis(4,5-dihydrooxazole) (L5)

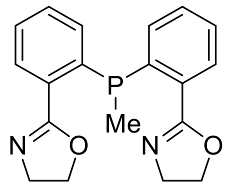

^{1}H NMR (400 MHz, $CDCl_3$) δ 7.68 (ddd, *J* = 7.3, 3.8, 2.0 Hz, 2H), 7.39 – 7.25 (m, 6H), 4.26 – 4.10 (m, 4H), 3.84 (dd, *J* = 10.6, 8.5 Hz, 4H), 1.61 (d, *J* = 5.5 Hz, 3H). **^{13}C NMR** (101 MHz, $CDCl_3$) δ 165.1, 142.7, 142.5, 132.4, 132.2, 131.7, 130.5, 129.5, 129.5, 127.8, 67.4, 55.1, -19.1. **^{31}P NMR** (162 MHz, $CDCl_3$) δ -23.5. **HRMS** (ESI/TOF) *m/z*: $[M + H]^+$ Calcd. for $C_{19}H_{20}N_2O_2P^+$ 339.1257; found 339.1260.

2,2'-((methylphosphanediyl)bis(5-methoxy-2,1-phenylene))bis(4,5-dihydrooxazole) (L6)

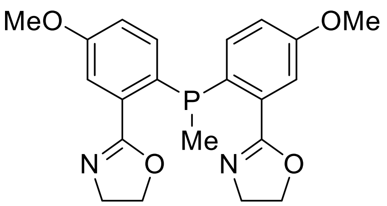

^{1}H NMR (400 MHz, $CDCl_3$) δ 7.27 (m, 2H), 7.21 (dd, *J* = 8.5, 3.6 Hz, 2H), 6.92 (dd, *J* = 8.6, 2.8 Hz, 2H), 4.33 – 4.18 (m, 4H), 3.92 (m, 4H), 3.82 (s, 6H), 1.58 (d, *J* = 5.6 Hz, 3H). **^{13}C NMR** (101 MHz, $CDCl_3$) δ 165.0, 159.1, 155.4, 133.0, 116.8, 114.3, 77.2, 67.4, 55.4, 55.0. **^{31}P NMR** (162 MHz, $CDCl_3$) δ -27.9. **HRMS** (ESI/TOF) *m/z*: $[M + H]^+$ Calcd. for $C_{21}H_{24}N_2O_4P^+$ 399.1468; found 399.1474.

4-(3-benzyloxyphenyl)-4-vinyl-1,3-dioxolan-2-one (1m)

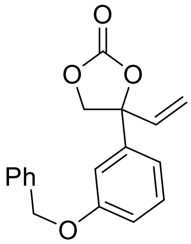

Following **GP3** starting on 8.2 mmol scale. Step 3: from diol precursor (1.26 g, 4.64 mmol, 1.00 equiv), **1m** was obtained as a yellowish oil (802 mg, 2.71 mmol, 58%). **^{1}H NMR** (400 MHz, $CDCl_3$) δ 7.47 – 7.40 (m, 4H), 7.44 – 7.30 (m, 2H), 7.03 – 6.95 (m, 2H), 6.92 (m, 1H), 6.13 (dd, *J* = 17.2, 10.7 Hz, 1H), 5.46 – 5.39 (m, 2H), 5.08 (s, 2H), 4.63 (d, *J* = 8.5 Hz, 1H), 4.54 (d, *J* = 8.5 Hz, 1H). **^{13}C NMR** (101 MHz, $CDCl_3$) δ 159.2, 154.1, 140.1, 136.54, 136.47, 130.3, 128.7, 128.2, 127.7, 117.6, 117.3, 115.0, 112.0, 85.4, 74.6, 70.3. **HRMS** (ESI/TOF) *m/z*: $[M + H]^+$ Calcd. for $C_{18}H_{17}O_4^+$ 297.1121; found 297.1123.

4-(3,5-dimethoxyphenyl)-4-vinyl-1,3-dioxolan-2-one (1s)

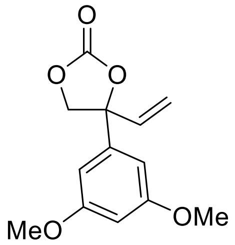

Following **GP3** starting on 8.2 mmol scale. Step 3: from diol precursor (1.31 g, 5.82 mmol, 1.00 equiv). **1s** was obtained as a yellowish oil (968 mg, 3.87 mmol, 66% yield). **^{1}H NMR** (500 MHz, $CDCl_3$) δ 6.46 (d, *J* = 2.2 Hz, 2H), 6.43 (t, *J* = 2.2 Hz, 1H), 6.11 (dd, *J* = 17.1, 10.7 Hz, 1H), 5.44 (d, *J* = 17.1 Hz, 1H), 5.40 (d, *J* = 10.7 Hz, 1H), 4.62 (d, *J* = 8.4 Hz, 1H), 4.53 (d, *J* = 8.5 Hz, 1H), 3.80 (s, 6H). **^{13}C NMR** (126 MHz, $CDCl_3$) δ 161.4, 154.1, 141.0, 136.4, 117.6, 103.1, 100.4, 85.5, 74.6, 55.6. **HRMS** (ESI/TOF) *m/z*: $[M + H]^+$ Calcd. for $C_{13}H_{15}O_5^+$ 251.0912; found 251.0913.

(*Z*)-2,5-diphenyl-5-vinylhex-2-ene-1,6-diol (*bl*-2a)

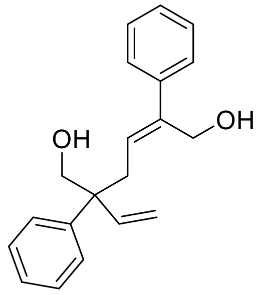

GP1 afforded **2a** as a yellowish oil (42 mg, 0.14 mmol 71% yield). **^{1}H NMR** (400 MHz, $CDCl_3$) δ 7.46 – 7.22 (m, 10H), 6.02 (dd, *J* = 17.8, 11.0 Hz, 1H), 5.80 (t, *J* = 8.0 Hz, 1H), 5.38 (dd, *J* = 11.0, 0.9 Hz, 1H), 5.20 (dd, *J* = 17.8, 0.9 Hz, 1H), 4.51 (s, 2H), 4.00 (d, *J* = 11.1 Hz, 1H), 3.88 (d, *J* = 11.1 Hz, 1H), 3.00 – 2.85 (m, 2H). **^{13}C NMR** (101 MHz, $CDCl_3$) δ 142.98, 142.97, 142.0, 141.7, 128.7, 128.5, 128.1, 127.3, 127.2, 126.9, 126.3, 115.6, 65.7, 60.2, 50.4, 33.4. **HRMS** (ESI/micrOTOF) *m/z*: $[M + Na]^+$ Calcd. for $C_{20}H_{22}NaO_2^+$ 317.1512; Found 317.1514.

(2*Z*,6*Z*)-2,7-diphenylocta-2,6-diene-1,8-diol (*ll*-2a)

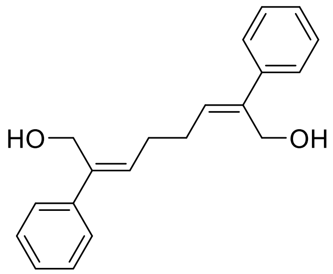

^{1}H NMR (400 MHz, $CDCl_3$) δ 7.45 – 7.40 (m, 4H), 7.36 – 7.30 (m, 4H), 7.28 – 7.22 (m, 2H), 5.97 – 5.88 (m, 2H), 4.56 (s, 4H), 2.52 – 2.45 (m, 4H). **^{13}C NMR** (101 MHz, $CDCl_3$) δ 140.9, 140.0, 131.3, 128.7, 127.4, 126.4, 59.9, 28.7. **HRMS** (ESI/micrOTOF) *m/z*: $[M + Na]^+$ Calcd. for $C_{20}H_{22}NaO_2^+$ 317.1512; Found 317.1507.

(*Z*)-2,5-di-*p*-tolyl-5-vinylhex-2-ene-1,6-diol (2b)

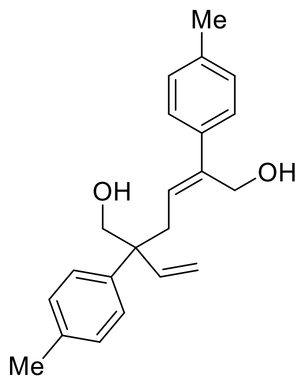

GP1 afforded **2b** as a yellowish oil (45 mg, 0.14 mmol, 70% yield). **^{1}H NMR** (400 MHz, $CDCl_3$) δ 7.34 – 7.29 (m, 2H), 7.25 – 7.21 (m, 2H), 7.17 (d, *J* = 8.1 Hz, 2H), 7.12 (d, *J* = 8.0 Hz, 2H), 5.98 (dd, *J* = 17.8, 11.0 Hz, 1H), 5.76 (t, *J* = 8.0 Hz, 1H), 5.34 (dd, *J* = 11.0, 0.9 Hz, 1H), 5.17 (dd, *J* = 17.8, 0.9 Hz, 1H), 4.47 (s, 2H), 3.96 (d, *J* = 11.0 Hz, 1H), 3.84 (d, *J* = 11.0 Hz, 1H), 2.87 (m, 2H), 2.34 (s + s, 6H). **^{13}C NMR** (101 MHz, $CDCl_3$) δ 143.2, 141.5, 139.9, 139.2, 136.9, 136.5, 129.4, 129.2, 127.3, 127.2, 126.2, 115.4, 65.8, 60.2, 50.1, 33.4, 21.2, 21.0. **HRMS** (ESI/micrOTOF) *m/z*: $[M + Na]^+$ Calcd. for $C_{22}H_{26}NaO_2^+$ 345.1825; Found 345.1828.

(*Z*)-2,5-bis(4-(*tert*-butyl)phenyl)-5-vinylhex-2-ene-1,6-diol (2c)

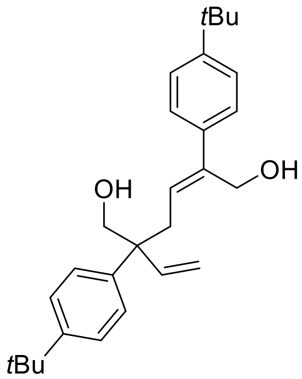

GP1 afforded **2c** as a colorless amorphous solid (57 mg, 0.14 mmol, 70% yield). **^{1}H NMR** (400 MHz, $CDCl_3$) δ 7.40 – 7.34 (m, 6H), 7.31 – 7.27 (m, 2H), 6.00 (dd, *J* = 17.8, 11.0 Hz, 1H), 5.84 (t, *J* = 8.0 Hz, 1H), 5.34 (dd, *J* = 11.0, 1.0 Hz, 1H), 5.17 (dd, *J* = 17.8, 1.0 Hz, 1H), 4.54 – 4.42 (m, 2H), 3.96 (d, *J* = 11.0 Hz, 1H), 3.86 (d, *J* = 11.0 Hz, 1H), 2.95 (m, 1H), 2.86 (m, 1H), 1.36 (s + s, 18H). **^{13}C NMR** (101 MHz, $CDCl_3$) δ 150.0, 149.6, 143.2, 141.3, 139.9, 139.1, 127.5, 127.0, 126.0, 125.5, 125.4, 115.2, 65.8, 60.1, 50.0, 34.6, 34.5, 33.4, 31.4. Note that 1 carbon is not resolved. **HRMS** (ESI/micrOTOF) *m/z*: $[M + Na]^+$ Calcd. for $C_{28}H_{38}NaO_2^+$ 429.2764; Found 429.2765.

(*Z*)-2,5-bis(4-fluorophenyl)-5-vinylhex-2-ene-1,6-diol (2d)

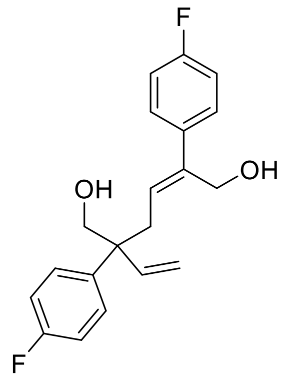

GP1 afforded **2d** as a yellowish oil (48 mg, 0.15 mmol, 73% yield). **^{1}H NMR** (400 MHz, $CDCl_3$) δ 7.38 – 7.27 (m, 4H), 7.01 (dt, J = 24.6, 8.7 Hz, 4H), 5.95 (dd, J = 17.8, 11.0 Hz, 1H), 5.67 (t, J = 7.9 Hz, 1H), 5.35 (dd, J = 11.0, 0.8 Hz, 1H), 5.14 (dd, J = 17.7, 0.8 Hz, 1H), 4.44 (s, 2H), 3.89 (d, J = 11.0 Hz, 1H), 3.81 (d, J = 11.1 Hz, 1H), 2.85 (dd, J = 8.0, 5.2 Hz, 2H). **^{13}C NMR** (101 MHz, $CDCl_3$) δ 162.3 (d, J = 245.9 Hz), 161.7 (d, J = 245.9 Hz), 142.8, 140.9, 138.7 (d, J = 3.3 Hz), 138.1 (d, J = 3.3 Hz), 129.0 (d, J = 7.8 Hz), 127.8 (d, J = 8.0 Hz), 127.8, 115.4 (d, J = 20.9 Hz), 115.8, 115.3 (d, J = 21.2 Hz), 65.7, 60.2, 49.9, 33.6. **^{19}F NMR** (376 MHz, $CDCl_3$) δ -115.6, -116.0. **HRMS** (ESI/micrOTOF) *m/z*: $[M + Na]^+$ Calcd. for $C_{20}H_{20}F_2NaO_2^+$ 353.1324; Found 353.1334.

(*Z*)-2,5-bis(4-chlorophenyl)-5-vinylhex-2-ene-1,6-diol (2e)

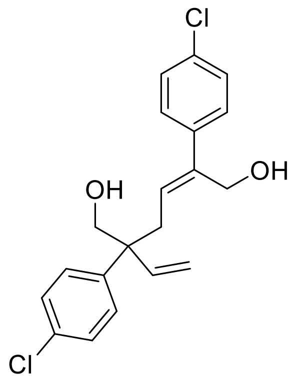

GP1 afforded **2e** as a yellowish oil (41 mg, 0.11 mmol, 56% yield, 83% conv., 67% yield brsm). **^{1}H NMR** (400 MHz, $CDCl_3$) δ 7.36 – 7.29 (m, 4H), 7.29 – 7.22 (m, 4H), 5.93 (dd, J = 17.8, 11.0 Hz, 1H), 5.71 (t, J = 8.0 Hz, 1H), 5.36 (d, J = 11.0 Hz, 1H), 5.15 (d, J = 17.8 Hz, 1H), 4.45 (s, 2H), 3.89 (d, J = 11.1 Hz, 1H), 3.80 (d, J = 11.1 Hz, 1H), 3.34 (bs, 1H), 2.85 (dd, J = 8.0, 1.7 Hz, 2H), 2.68 (bs, 1H). **^{13}C NMR** (101 MHz, $CDCl_3$) δ 142.5, 141.5, 141.0, 140.4, 133.1, 132.9, 128.8, 128.6, 128.2, 127.5, 116.1, 65.6, 60.0, 50.0, 33.4. One carbon is unresolved. **HRMS** (ESI/micrOTOF) *m/z*: $[M + Na]^+$ Calcd. for $C_{20}H_{20}Cl_2NaO_2^+$ 385.0733; Found 385.0728.

(*Z*)-2,5-bis(4-bromophenyl)-5-vinylhex-2-ene-1,6-diol (2f)

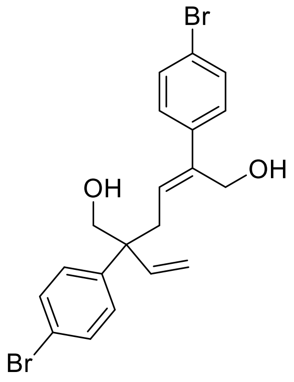

GP1 afforded **2f** as a yellowish oil (45 mg, 0.10 mmol, 50% yield). **^{1}H NMR** (400 MHz, CDCl3) δ 7.51 – 7.38 (m, 4H), 7.30 – 7.24 (m, 2H), 7.24 – 7.16 (m, 2H), 5.93 (dd, *J* = 17.8, 11.0 Hz, 1H), 5.71 (t, *J* = 8.0 Hz, 1H), 5.36 (d, *J* = 11.0 Hz, 1H), 5.15 (dd, *J* = 17.8, 1.8 Hz, 1H), 4.44 (s, 2H), 3.92 – 3.84 (m, 1H), 3.83 – 3.76 (m, 1H), 3.32 (bs, 1H), 2.88 – 2.81 (m, 2H). ^{13}C NMR (101 MHz, $CDCl_3$) δ 142.4, 142.1, 141.1, 140.9, 131.7, 131.6, 129.2, 128.3, 127.9, 121.3, 121.0, 116.2, 65.5, 60.0, 50.1, 33.4. **HRMS** (ESI/micrOTOF) *m/z*: [M + Na]$^+$ Calcd. for $C_{20}H_{20}Br_2NaO_2^+$ 472.9722; Found 472.9729.

(*Z*)-2,5-bis(4-methoxyphenyl)-5-vinylhex-2-ene-1,6-diol (2g)

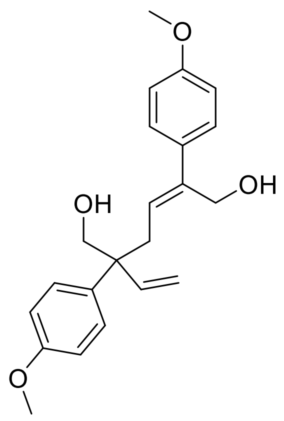

GP1 afforded **2g** as a yellowish oil (16 mg, 0.045 mmol, 22% yield). **GP1** using **L5** instead of **L6** on 0.123 mmol scale afforded **2g** as a yellowish oil (14 mg, 38 μmol, 31% yield). **^{1}H NMR** (400 MHz, $CDCl_3$) δ 7.38 – 7.31 (m, 2H), 7.27 – 7.23 (m, 2H), 6.93 – 6.87 (m, 2H), 6.87 – 6.81 (m, 2H), 5.97 (dd, *J* = 17.8, 11.0 Hz, 1H), 5.69 (t, *J* = 8.0 Hz, 1H), 5.34 (dd, *J* = 11.0, 1.0 Hz, 1H), 5.17 (dd, *J* = 17.8, 1.0 Hz, 1H), 4.47 (d, *J* = 1.2 Hz, 2H), 3.94 (d, *J* = 11.0 Hz, 1H), 3.83 (d, *J* = 11.0 Hz, 1H), 3.80 (s, 3H), 3.79 (s, 3H), 2.87 – 2.81 (m, 2H). **^{13}C NMR** (101 MHz, $CDCl_3$) δ 159.0, 158.4, 143.2, 141.2, 134.8, 134.5, 128.4, 127.4, 126.4, 115.5, 114.1, 113.9, 66.0, 60.2, 55.44, 55.41, 49.9, 33.6. **HRMS** (ESI/micrOTOF) *m/z*: [M + Na]$^+$ Calcd. for $C_{22}H_{26}NaO_4^+$ 377.1723; Found 377.1740.

(*Z*)-2,5-bis(4-(benzyloxy)phenyl)-5-vinylhex-2-ene-1,6-diol (2h)

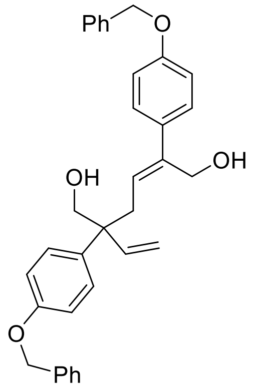

GP1 afforded **2h** as a yellowish oil (41 mg, 0.080 mmol, 40% yield).^{1}**H NMR** (400 MHz, $CDCl_3$) δ 7.48 – 7.28 (m, 12H), 7.25 (d, *J* = 8.9 Hz, 2H), 7.01 – 6.93 (m, 2H), 6.96 – 6.88 (m, 2H), 5.97 (dd, *J* = 17.8, 11.0 Hz, 1H), 5.70 (t, *J* = 8.0 Hz, 1H), 5.34 (dd, *J* = 11.0, 0.9 Hz, 1H), 5.17 (dd, *J* = 17.8, 1.0 Hz, 1H), 5.06 (s, 4H), 4.47 (s, 2H), 3.94 (d, *J* = 11.0 Hz, 1H), 3.83 (d, *J* = 11.0 Hz, 1H), 2.88 – 2.81 (m, 2H). ^{13}**C NMR** (101 MHz, $CDCl_3$) δ 158.2, 157.7, 143.2, 141.1, 137.2, 137.1, 135.1, 134.8, 128.74, 128.72, 128.4, 128.1, 128.1, 127.63, 127.57, 127.4, 126.4, 115.5, 115.0, 114.8, 70.2, 65.9, 60.2, 49.9, 33.5. One carbon is not resolved. **HRMS** (ESI/micrOTOF) *m/z*: [M + Na]$^+$ Calcd. for $C_{34}H_{34}NaO_4^+$ 529.2349; Found 529.2361.

(*Z*)-2,5-bis(4-(trifluoromethyl)phenyl)-5-vinylhex-2-ene-1,6-diol (2i)

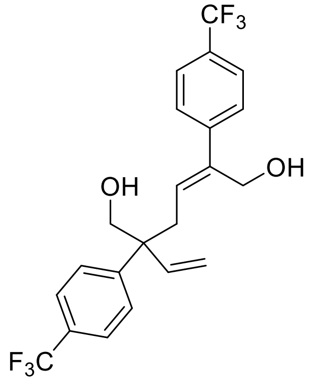

GP1 afforded **2i** as a yellowish oil (18 mg, 0.041 mmol, 21% yield).^{1}**H NMR** (400 MHz, $CDCl_3$) δ 7.62 (d, *J* = 8.3 Hz, 2H), 7.59 – 7.45 (m, 6H), 5.97 (dd, *J* = 17.8, 11.0 Hz, 1H), 5.79 (t, *J* = 8.0 Hz, 1H), 5.41 (dd, *J* = 11.0, 0.7 Hz, 1H), 5.20 (dd, *J* = 17.8, 0.7 Hz, 1H), 4.51 (s, 2H), 3.98 (d, *J* = 11.2 Hz, 1H), 3.88 (d, *J* = 11.2 Hz, 1H), 3.02 – 2.88 (m, 2H). ^{13}**C NMR** (101 MHz, $CDCl_3$) δ 147.2, 145.5, 142.0, 141.4, 129.5 - 129.2 (m), 127.8, 126.5, 125.5 – 125.3 (m), 116.6, 65.6, 60.1, 50.5, 33.5. 3 carbons and their multiplicity are not resolved. ^{19}**F NMR** (376 MHz, $CDCl_3$) δ -62.61, -62.66. **HRMS** (ESI/micrOTOF) *m/z*: [M + Na]$^+$ Calcd. for $C_{22}H_{20}F_6NaO_2^+$ 453.1260; Found 453.1250.

(Z)-2,5-di([1,1'-biphenyl]-4-yl)-5-vinylhex-2-ene-1,6-diol (2j)

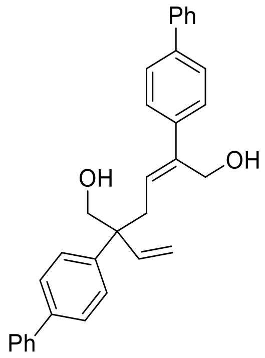

A modified **GP1** (the reaction was set up in the same fashion as **GP1**, however the filtration through silica prior to purification was performed with CH_2Cl_2, as the product is extremely poorly soluble) afforded **2j** as an off-white amorphous solid (27 mg, 0.059 mmol, 30% yield). **^{1}H NMR** (400 MHz, $CDCl_3$) δ 7.65 – 7.30 (m, 18H), 6.05 (dd, *J* = 17.8, 11.0 Hz, 1H), 5.89 (t, *J* = 8.0 Hz, 1H), 5.42 (dd, *J* = 11.0, 0.9 Hz, 1H), 5.25 (dd, *J* = 17.8, 0.9 Hz, 1H), 4.57 (s, 2H), 4.06 (d, *J* = 11.1 Hz, 1H), 3.93 (d, *J* = 11.1 Hz, 1H), 2.97 (dd, *J* = 8.0, 1.7 Hz, 2H). **^{13}C NMR** (101 MHz, $CDCl_3$) δ 142.9, 142.0, 141.5, 140.90, 140.89, 140.7, 140.1, 139.9, 129.0, 128.9, 127.9, 127.8, 127.5, 127.39, 127.38, 127.3, 127.2, 127.1, 126.7, 115.9, 65.9, 60.2, 50.4, 33.6. **HRMS** (ESI/micrOTOF) *m/z*: $[M + Na]^+$ Calcd. for $C_{32}H_{30}NaO_2^+$ 469.2138; Found 469.2147.

(*Z*)-2,5-di-*m*-tolyl-5-vinylhex-2-ene-1,6-diol (2k)

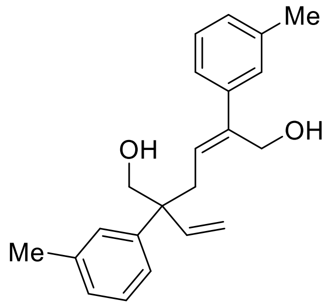

GP1 afforded **2k** as a yellowish oil (43 mg, 0.13 mmol, 67% yield). **^{1}H NMR** (400 MHz, $CDCl_3$) δ 7.27 – 7.09 (m, 6H), 7.09 – 7.00 (m, 2H), 5.97 (dd, *J* = 17.8, 11.0 Hz, 1H), 5.76 (t, *J* = 8.0 Hz, 1H), 5.32 (dd, *J* = 11.0, 1.0 Hz, 1H), 5.15 (dd, *J* = 17.8, 1.0 Hz, 1H), 4.46 (s, 2H), 3.95 (d, *J* = 11.0 Hz, 1H), 3.84 (d, *J* = 11.0 Hz, 1H), 2.95 – 2.79 (m, 2H), 2.35 (s, 3H), 2.32 (s, 3H). **^{13}C NMR** (101 MHz, $CDCl_3$) δ 143.1, 142.9, 142.1, 141.8, 138.2, 138.0, 128.5, 128.4, 128.1, 127.29, 127.90, 127.7, 127.0, 124.3, 123.4, 115.4, 65.7, 60.2, 50.3, 33.4, 21.8, 21.6. **HRMS** (ESI/micrOTOF) *m/z*: $[M + Na]^+$ Calcd. for $C_{22}H_{26}NaO_2^+$ 345.1825; Found 345.1812.

(*Z*)-2,5-bis(3-methoxyphenyl)-5-vinylhex-2-ene-1,6-diol (2l)

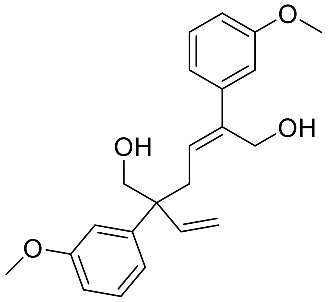

GP1 afforded **2l** as a yellowish oil (52 mg, 0.15 mmol, 73% yield). **^{1}H NMR** (400 MHz, $CDCl_3$) δ 7.30 – 7.19 (m, 2H), 7.02 – 6.97 (m, 1H), 6.97 – 6.89 (m, 3H), 6.81-6.78 (m, 2H), 5.98 (dd, *J* = 17.8, 11.0 Hz, 1H), 5.78 (t, *J* = 8.0 Hz, 1H), 5.34 (dd, *J* = 11.0, 0.9 Hz, 1H), 5.17 (dd, *J* = 17.8, 0.9 Hz, 1H), 4.47 (s, 2H), 3.95 (d, *J* = 11.1 Hz, 1H), 3.84 (d, *J* = 11.1 Hz, 1H), 3.80 (s, 3H), 3.79 (s, 3H), 2.94 – 2.82 (m, 2H). ^{13}C NMR (101 MHz, $CDCl_3$) δ 159.8, 159.7, 144.7, 143.6, 142.8, 141.7, 129.6, 129.4, 128.3, 119.6, 118.8, 115.6, 114.1, 112.6, 112.2, 111.6, 65.8, 60.2, 55.3, 50.4, 33.4. One carbon is not resolved. **HRMS** (ESI/micrOTOF) *m/z*: [M + Na]$^+$ Calcd. for $C_{22}H_{26}NaO_4^+$ 377.1723; Found 377.1715.

(*Z*)-2,5-bis(3-(benzyloxy)phenyl)-5-vinylhex-2-ene-1,6-diol (2m)

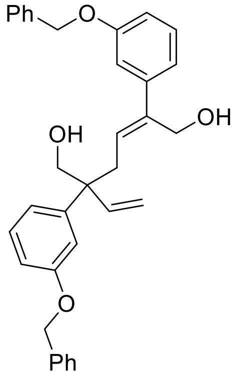

GP1 afforded **2m** as a yellowish oil (52 mg, 0.10 mmol, 51% yield). **^{1}H NMR** (400 MHz, $CDCl_3$) δ 7.47 – 7.19 (m, 12H), 7.10 – 6.84 (m, 6H), 5.97 (dd, *J* = 17.8, 11.0 Hz, 1H), 5.79 (t, *J* = 8.0 Hz, 1H), 5.38 – 5.27 (m, 1H), 5.17 (dd, *J* = 17.8, 0.9 Hz, 1H), 5.06 (s, 4H), 4.47 (s, 2H), 3.94 (d, *J* = 11.1 Hz, 1H), 3.84 (d, *J* = 11.1 Hz, 1H), 2.88 (qd, *J* = 13.9, 8.0 Hz, 2H). **^{13}C NMR** (101 MHz, $CDCl_3$) δ 159.1, 158.9, 144.7, 143.7, 142.7, 141.7, 137.1, 137.0, 129.6, 129.5, 128.71, 128.68, 128.2, 128.14, 128.05, 127.72, 127.69, 119.8, 119.1, 115.7, 115.0, 113.4, 113.2, 112.6, 70.2, 70.1, 65.8, 60.2, 50.4, 33.4, 29.8. One carbon is not resolved. **HRMS** (ESI/micrOTOF) *m/z*: [M + Na]$^+$ Calcd. for $C_{34}H_{34}NaO_4^+$ 529.2349; Found 529.2359.

(*Z*)-2,5-bis(3-(trifluoromethyl)phenyl)-5-vinylhex-2-ene-1,6-diol (2n)

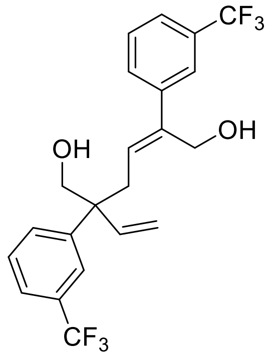

GP1 afforded **2n** as a yellowish oil (22 mg, 0.051 mmol, 26% yield). **^{1}H NMR** (500 MHz, $CDCl_3$) δ 7.65 (d, *J* = 1.8 Hz, 1H), 7.60 – 7.53 (m, 4H), 7.52 – 7.47 (m, 2H), 7.42 (t, *J* = 7.8 Hz, 1H), 5.98 (dd, *J* = 17.8, 11.0 Hz, 1H), 5.77 (t, *J* = 8.0 Hz, 1H), 5.43 (d, *J* = 11.0 Hz, 1H), 5.21 (d, *J* = 17.8 Hz, 1H), 4.55 – 4.46 (m, 2H), 3.99 (d, *J* = 11.2 Hz, 1H), 3.90 (d, *J* = 11.2 Hz, 1H), 2.98 (dd, *J* = 13.8, 8.2 Hz, 1H), 2.90 (dd, *J* = 13.8, 7.8 Hz, 1H). **^{13}C NMR** (126 MHz, $CDCl_3$) δ 144.2, 142.8, 142.0, 141.3, 131.1 (q, *J* = 32.0 Hz), 130.9 (q, *J* = 31.8 Hz), 130.8, 130.7 (d, *J* = 21.1 Hz), 129.6, 129.5, 129.14, 129.10, 129.0, 124.3 (q, *J* = 272.4 Hz), 124.2 (q, *J* = 3.8 Hz), 124.1 – 123.9 (m, 2C), 123.0 (q, *J* = 3.7 Hz), 116.6, 65.5, 59.9, 50.3, 33.4. **^{19}F NMR** (471 MHz, $CDCl_3$) δ -62.49, -62.62. **HRMS** (ESI/micrOTOF) *m/z*: $[M + Na]^+$ Calcd. for $C_{22}H_{20}F_6NaO_4^+$ 453.1260; Found 453.1263.

(*Z*)-2,5-di(thiophen-3-yl)-5-vinylhex-2-ene-1,6-diol (2o)

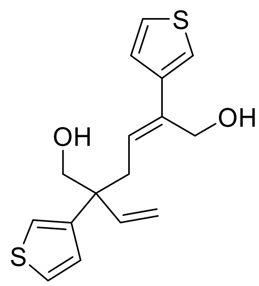

GP1 afforded **2o** as a yellowish oil (27 mg, 0.088 mmol, 44% yield, conv. 63%, 69% yield brsm). **^{1}H NMR** (400 MHz, $CDCl_3$) δ 7.34 (dd, *J* = 5.0, 2.9 Hz, 1H), 7.29 – 7.21 (m, 2H), 7.16 (dd, *J* = 5.0, 1.4 Hz, 1H), 7.11 – 7.01 (m, 2H), 6.00 (dd, *J* = 17.7, 10.9 Hz, 1H), 5.89 (t, *J* = 8.1 Hz, 1H), 5.29 (dd, *J* = 10.9, 0.9 Hz, 1H), 5.11 (dd, *J* = 17.7, 0.9 Hz, 1H), 4.46 (s, 2H), 3.87 (d, *J* = 11.1 Hz, 1H), 3.79 (d, *J* = 11.1 Hz, 1H), 3.29 (bs, 1H), 2.92 – 2.77 (m, 2H), 2.67 (bs, 1H). **^{13}C NMR** (101 MHz, $CDCl_3$) δ 144.4, 142.8, 142.5, 136.6, 127.1, 126.3, 125.9, 125.8, 125.7, 121.1, 120.2, 115.4, 66.0, 59.9, 49.0, 33.6. **HRMS** (ESI/micrOTOF) *m/z*: $[M + Na]^+$ Calcd. for $C_{16}H_{18}NaO_2S_2^+$ 329.0640; Found 329.0639.

(*E*)-2,5-di(furan-2-yl)-5-vinylhex-2-ene-1,6-diol (2p)

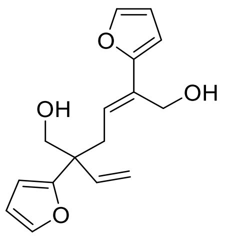

GP1 afforded **2p** as a yellowish oil (21 mg, 0.075 mmol, 38% yield). ^{1}H NMR (400 MHz, CD_3CN) δ 7.49 (dd, *J* = 1.9, 0.8 Hz, 1H), 7.44 – 7.39 (m, 1H), 6.47 – 6.37 (m, 3H), 6.24 (dd, *J* = 3.3, 0.8 Hz, 1H), 6.10 – 6.03 (m, 1H), 6.02 (d, *J* = 8.0 Hz, 1H), 5.24 (dd, *J* = 10.9, 1.2 Hz, 1H), 5.05 (dd, *J* = 17.6, 1.2 Hz, 1H), 4.38 (d, *J* = 2.8 Hz, 2H), 3.85 – 3.69 (m, 2H), 3.39 (s, 2H), 2.95 – 2.80 (m, 2H). **^{13}C NMR** (101 MHz, CD_3CN) δ 156.9, 154.5, 141.8, 141.7, 140.8, 131.6, 123.9, 114.8, 111.3, 110.1, 106.4, 105.7, 64.8, 57.0, 48.4, 31.6. **HRMS** (ESI/micrOTOF) m/z: $[M + Na]^+$ Calcd. for $C_{16}H_{18}NaO_4^+$ 297.1097; Found 297.1103.[21]

Structural determination

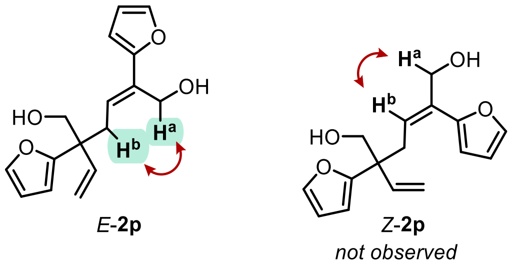

E-**2p**   *Z*-**2p** *not observed*

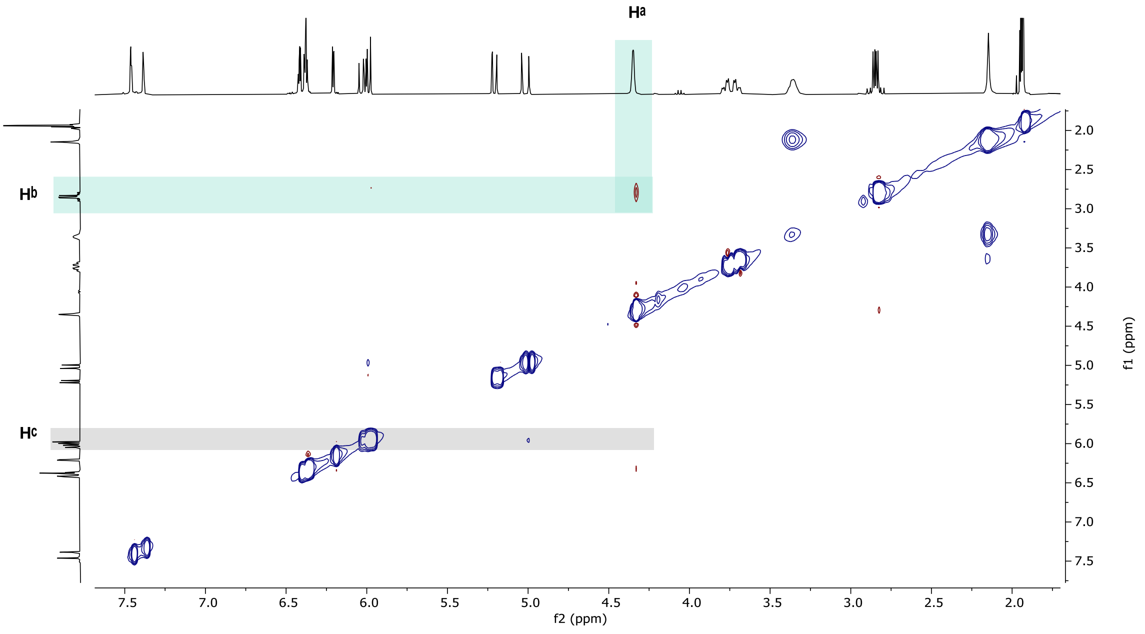

(21) Compound was unstable in $CDCl_3$, however we were able to record a ^{1}H NMR: **^{1}H NMR** (400 MHz, $CDCl_3$) δ 7.41 (dd, *J* = 1.9, 0.8 Hz, 1H), 7.34 (dd, *J* = 1.8, 0.8 Hz, 1H), 6.41 – 6.33 (m, 3H), 6.18 (dd, *J* = 3.3, 0.8 Hz, 1H), 6.13 (t, *J* = 8.3 Hz, 1H), 5.99 (dd, *J* = 17.6, 10.9 Hz, 1H), 5.30 (dd, *J* = 10.8, 0.8 Hz, 1H), 5.10 (dd, *J* = 17.7, 0.8 Hz, 1H), 4.47 (d, *J* = 12.1 Hz, 1H), 4.41 (d, *J* = 12.2 Hz, 1H), 3.92 (d, *J* = 11.2 Hz, 1H), 3.80 (d, *J* = 11.2 Hz, 1H), 3.01 – 2.91 (m, 1H), 2.85 – 2.75 (m, 1H).

(*Z*)-2,5-di(naphthalen-2-yl)-5-vinylhex-2-ene-1,6-diol (2q)

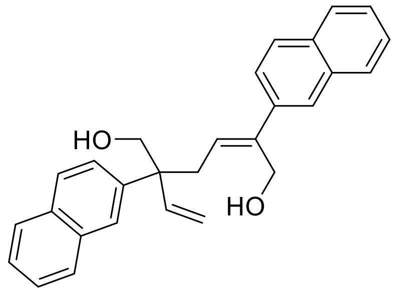

GP1 afforded **2q** as an off-white amorphous solid (53 mg, 0.13 mmol, 67% yield). **^{1}H NMR** (400 MHz, CDCl3) δ 7.90 – 7.71 (m, 8H), 7.56 – 7.37 (m, 6H), 6.08 (dd, *J* = 17.8, 11.0 Hz, 1H), 5.94 (t, *J* = 8.0 Hz, 1H), 5.43 (d, *J* = 11.0 Hz, 1H), 5.26 (d, *J* = 17.8 Hz, 1H), 4.64 (s, 2H), 4.14 (d, *J* = 11.1 Hz, 1H), 3.99 (d, *J* = 11.1 Hz, 1H), 3.16 – 3.00 (m, 2H). **^{13}C NMR** (101 MHz, $CDCl_3$) δ 142.9, 141.9, 140.3, 139.3, 133.6, 133.4, 132.7, 132.4, 128.5, 128.4, 128.3, 128.2, 128.1, 127.6, 126.4, 126.24, 126.21, 125.9, 125.8, 125.7, 124.8, 124.7, 116.1, 65.8, 60.3, 50.6, 33.6. One carbon is not resolved. **HRMS** (ESI/micrOTOF) *m/z*: [M + Na]$^+$ Calcd. for $C_{28}H_{26}NaO_2^+$ 417.1825; Found 417.1823.

(*Z*)-2,5-bis(3,5-dimethylphenyl)-5-vinylhex-2-ene-1,6-diol (2r)

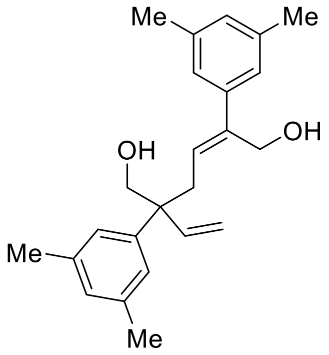

GP1 afforded **2r** as an off-white amorphous solid (45 mg, 0.13 mmol, 64% yield). **^{1}H NMR** (500 MHz, CDCl3) δ 7.07 – 7.03 (m, 2H), 6.98 – 6.94 (m, 2H), 6.94 – 6.89 (m, 2H), 5.98 (dd, *J* = 17.8, 11.0 Hz, 1H), 5.77 (t, *J* = 8.0 Hz, 1H), 5.36 (dd, *J* = 11.0, 1.0 Hz, 1H), 5.19 (dd, *J* = 17.8, 1.0 Hz, 1H), 4.52 – 4.43 (m, 2H), 3.98 (d, *J* = 11.0 Hz, 1H), 3.85 (d, *J* = 11.0 Hz, 1H), 2.94 – 2.79 (m, 2H), 2.34 (s, 6H), 2.32 (s, 6H). **^{13}C NMR** (126 MHz, $CDCl_3$) δ 143.1, 142.9, 142.2, 141.9, 138.0, 137.9, 128.8, 128.6, 127.8, 125.1, 124.2, 115.3, 65.7, 60.3, 50.2, 33.4, 21.6, 21.5. **HRMS** (ESI/micrOTOF) *m/z*: [M + Na]$^+$ Calcd. for $C_{24}H_{30}NaO_2^+$ 373.2138; Found 373.2127.

(*Z*)-2,5-bis(3,5-dimethoxyphenyl)-5-vinylhex-2-ene-1,6-diol (2s)

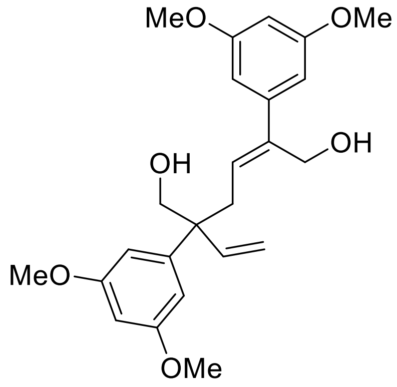

GP1 afforded **2s** as a yellowish oil (53 mg, 0.13 mmol, 64% yield). **^{1}H NMR** (400 MHz, $CDCl_3$) δ 6.57 (d, *J* = 2.2 Hz, 2H), 6.49 (d, *J* = 2.2 Hz, 2H), 6.36 (m, 2H), 5.96 (dd, *J* = 17.8, 11.0 Hz, 1H), 5.77 (t, *J* = 8.0 Hz, 1H), 5.33 (dd, *J* = 11.0, 0.9 Hz, 1H), 5.18 (dd, *J* = 17.8, 0.9 Hz, 1H), 4.46 (d, *J* = 1.6 Hz, 2H), 3.93 (d, *J* = 11.1 Hz, 1H), 3.85 – 3.75 (m, 13H), 2.92 – 2.77 (m, 2H). ^{13}C NMR (101 MHz, $CDCl_3$) δ 161.0, 160.8, 145.5, 144.4, 142.6, 141.9, 128.3, 115.6, 106.0, 104.7, 99.3, 98.2, 65.8, 60.2, 55.4, 50.5, 33.3. One carbon is not resolved. **HRMS** (ESI/micrOTOF) *m/z*: $[M + H]^+$ Calcd. for $C_{24}H_{31}O_6^+$ 415.2115; Found 415.2132.

(*Z*)-2,5-bis(benzo[*d*][1,3]dioxol-5-yl)-5-vinylhex-2-ene-1,6-diol (2t)

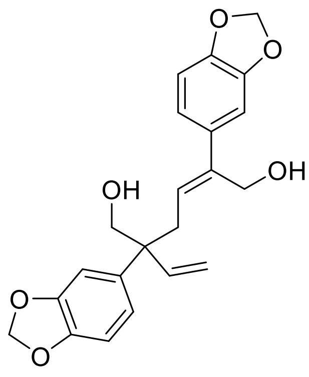

GP1 afforded **2t** as a yellowish oil (21 mg, 0.055 mmol, 27% yield). **^{1}H NMR** (400 MHz, CDCl3) δ 6.93 – 6.86 (m, 2H), 6.86 – 6.71 (m, 4H), 5.99 – 5.87 (m, 5H), 5.66 (t, *J* = 7.9 Hz, 1H), 5.37 – 5.27 (m, 1H), 5.16 (dd, *J* = 17.8, 0.9 Hz, 1H), 4.45 (s, 2H), 3.89 (d, *J* = 11.0 Hz, 1H), 3.80 (d, *J* = 11.0 Hz, 1H), 2.81 (dd, *J* = 8.0, 1.5 Hz, 2H). **^{13}C NMR** (101 MHz, $CDCl_3$) δ 148.1, 147.8, 146.9, 146.4, 143.0, 141.4, 136.8, 136.4, 126.9, 120.2, 119.7, 115.6, 108.3, 108.2, 108.1, 107.0, 101.2, 101.1, 65.9, 60.4, 50.2, 33.6. **HRMS** (ESI/micrOTOF) *m/z*: $[M + Na]^+$ Calcd. for $C_{22}H_{22}NaO_6^+$ 405.1309; Found 405.1314.

(Z)-2,5-diphenyl-5-vinylhex-2-ene-1,6-diyl diacetate (5)

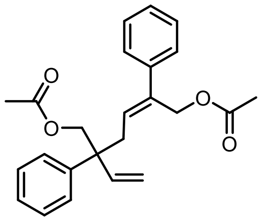

5 was obtained as a yellowish oil (120 mg, 0.317 mmol, 98% yield). **^{1}H NMR** (400 MHz, $CDCl_3$) δ 7.37 – 7.32 (m, 4H), 7.29 – 7.19 (m, 6H), 6.04 (dd, *J* = 17.8, 11.0 Hz, 1H), 5.84 (t, *J* = 7.5 Hz, 1H), 5.32 (d, *J* = 11.0 Hz, 1H), 5.18 (dd, *J* = 17.8, 0.8 Hz, 1H), 4.94 (t, *J* = 2.4 Hz, 2H), 4.49 – 4.41 (m, 1H), 4.41 – 4.34 (m, 1H), 2.91 (dd, *J* = 7.5, 2.7 Hz, 2H), 1.99 (s, 3H), 1.98 (s, 3H). **^{13}C NMR** (101 MHz, $CDCl_3$) δ 171.0, 170.9, 142.0, 141.7, 140.6, 136.7, 129.9, 128.39, 128.35, 127.33, 127.31, 126.9, 126.2, 115.3, 68.2, 61.1, 48.2, 34.5, 20.92, 20.88. **HRMS** (ESI/micrOTOF) *m/z*: $[M + Na]^+$ Calcd. for $C_{24}H_{26}NaO_4^+$ 401.1723; Found 401.1722.

(3-(2-(acetoxymethyl)-2-phenylbut-3-en-1-yl)-2-phenyloxiran-2-yl)methyl acetate (6)

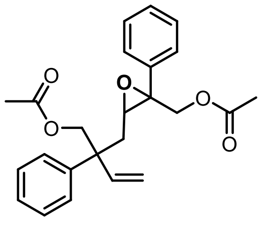

6 was obtained as a yellowish oil (*dr* = 1:1; 20 mg, 0.051 mmol, 90 wt% purity, 37% yield). **^{1}H NMR** (400 MHz, $CDCl_3$) δ 7.31 – 7.26 (m, 4H), 7.26 – 7.17 (m, 12H), 7.15 – 7.09 (m, 4H), 5.97 (dt, *J* = 17.7, 10.9 Hz, 2H), 5.28 (ddd, *J* = 14.1, 11.0, 0.6 Hz, 2H), 5.23 – 5.05 (m, 3H), 4.56 – 4.28 (m, 6H), 4.14 (dd, *J* = 22.2, 12.2 Hz, 2H), 2.87 – 2.75 (m, 2H), 2.34 (ddd, *J* = 14.6, 5.5, 1.8 Hz, 2H), 1.93 (s, 3H), 1.92 (s, 3H), 1.91 (s, 3H), 1.90 (s, 3H). **^{13}C NMR** (101 MHz, $CDCl_3$) δ 170.98, 170.95, 170.86, 141.9, 141.8, 141.5, 140.8, 138.44, 138.40, 137.6, 130.4, 128.8, 128.5, 128.4, 128.0, 127.33, 127.27, 127.25, 127.23, 125.9, 125.8, 116.0, 115.5, 68.8, 68.1, 64.9, 64.8, 63.0, 62.9, 61.7, 61.6, 47.6, 47.5, 34.71, 34.69, 29.8, 21.0, 20.9. 2 carbons signals are not resolved. **HRMS** (ESI/micrOTOF) *m/z*: $[M + Na]^+$ Calcd. for $C_{24}H_{26}NaO_5^+$ 417.1672; Found 417.1672.

6-(1,3-dioxoisoindolin-2-yl)-2,5-diphenyl-2-vinylhex-4-en-1-yl acetate (7)

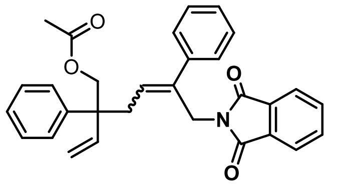

7 was obtained as a colorless amorphous solid (101 mg, 217 µmol, 63%). **^{1}H NMR** (400 MHz, $CDCl_3$, as a mixture of *E*:*Z* isomers in 6:4 ratio, (*E*) **major**, (*Z*) *minor*) δ ***7.85*** (m, 5H), ***7.56 – 7.26*** (m, 8H), ***7.25 – 7.15*** (m, 1H), *6.23* (dd, *J* = 17.8, 11.0 Hz, 0.4H), **5.99** (dd, *J* = 17.7, 11.0 Hz, 0.6H), *5.84* (t, *J* = 7.1 Hz, 0.4H), ***5.53 – 5.25*** (m, 2H), **5.11** (d, *J* = 17.7 Hz, 0.6H), *4.74* (s, 0.8H), *4.65* (d, *J* = 11.0 Hz, 0.4H), ***4.57*** (m, 1.6H), **4.42** (d, *J* = 10.8 Hz, 0.6H), **4.33** (d, *J* = 10.9 Hz, 0.6H), *3.24* (d, *J* = 7.2 Hz, 0.8H), **2.67** (d, *J* = 7.3 Hz, 1.2H), 2.15 (s, 0.8H), 1.90 (s, 2.2H). **^{13}C NMR** (101 MHz, $CDCl_3$, as a mixture of *E*:*Z* in 6:4 ratio) δ 171.0, 170.9, 168.2, 168.0, 167.9, 142.2, 141.9, 141.8, 140.2, 137.9, 137.6, 137.0, 134.3, 133.91, 133.87, 132.8, 132.0, 131.9, 128.84, 128.78, 128.4, 128.3, 128.13, 128.11, 127.6, 127.4, 127.3, 127.1, 126.9, 126.5, 124.2, 123.6, 123.3, 123.2, 115.5, 114.9, 74.2, 71.0, 68.6, 67.8, 48.7, 48.5, 48.1, 44.4, 36.8, 34.7, 34.3, 29.8, 21.0, 20.8. 2 carbons are not resolved. **HRMS** (ESI/micrOTOF) *m/z*: $[M + Na]^+$ Calcd. for $C_{30}H_{27}NNaO_4^+$ 488.1832; Found 488.1828.

(*Z*)-2,5-diphenyl-5-vinylhex-2-enedial (*Z*-8)

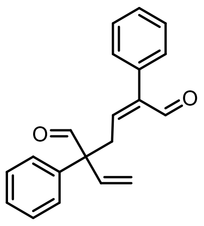

Z-**8** was obtained as a yellowish oil (d.r. = 13:1, 28 mg, 0.96 mmol, 98% yield). **^{1}H NMR** (400 MHz, $CDCl_3$) ^{1}H NMR (400 MHz, $CDCl_3$) δ 10.06 (d, *J* = 0.5 Hz, 1H), 9.61 (s, 1H), 7.47 – 7.39 (m, 2H), 7.39 – 7.23 (m, 6H), 7.18 – 7.11 (m, 2H), 6.59 – 6.50 (m, 1H), 6.22 (dd, *J* = 17.8, 10.9 Hz, 1H), 5.63 (d, *J* = 10.9 Hz, 1H), 5.34 (d, *J* = 17.8 Hz, 1H), 3.45 (dd, *J* = 7.9, 1.3 Hz, 2H), 1.21 (t, *J* = 7.0 Hz, 1H). **^{13}C NMR** (101 MHz, $CDCl_3$) δ 197.5, 190.4, 145.0, 141.8, 137.3, 136.8, 135.7, 129.4, 128.4, 128.34, 128.29, 128.2, 128.14, 128.06, 120.7, 61.9, 32.2. **HRMS** (ESI/micrOTOF) *m/z*: $[M + Na]^+$ Calcd. for $C_{20}H_{18}NaO_2^+$ 313.1199; Found 313.1199.

(*E*)-2,5-diphenyl-5-vinylhex-2-enedial (*E*-8)

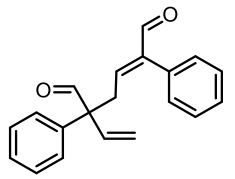

E-**8** was obtained as a yellowish oil (17 mg, 59 μmol, 59 %). **^{1}H NMR** (400 MHz, $CDCl_3$) δ 9.52 (s, 1H), 9.48 (s, 1H), 7.44 – 7.38 (m, 2H), 7.38 – 7.30 (m, 6H), 7.20 – 7.13 (m, 2H), 7.01 – 6.94 (m, 2H), 6.60 (t, *J* = 7.3 Hz, 1H), 6.13 (dd, *J* = 17.8, 10.9 Hz, 1H), 5.57 (d, *J* = 10.9 Hz, 1H), 5.19 (d, *J* = 17.8 Hz, 1H), 3.09 – 3.02 (m, 2H). **^{13}C NMR** (101 MHz, $CDCl_3$) δ 197.2, 193.2, 150.7, 145.7, 135.5, 129.3, 128.3, 128.1, 128.0, 120.4, 61.4, 53.4, 34.1, 29.7. **HRMS** (ESI/micrOTOF) *m/z*: $[M + Na]^+$ Calcd. for $C_{20}H_{18}NaO_2^+$ 313.1199; Found 313.1199.

(*E*)-3,6-diphenyl-6-vinyloct-3-ene-1,8-diol (*bl*-10a)

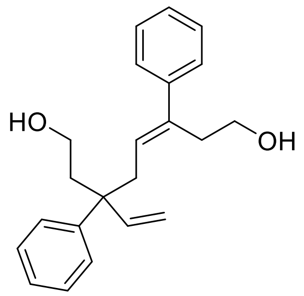

Mixture of regio-isomers. **^{1}H NMR** (400 MHz, $CDCl_3$) δ 7.39 – 7.12 (m, 12H), 6.03 (dd, *J* = 17.7, 10.9 Hz, 1H), 5.64 (t, *J* = 7.1 Hz, 1H), 5.29 (dd, *J* = 10.9, 1.0 Hz, 1H), 5.21 (dd, *J* = 17.7, 1.0 Hz, 1H), 3.63 – 3.49 (m, 4H), 2.83 – 2.68 (m, 4H), 2.24 – 2.10 (m, 2H). ^{13}C NMR (101 MHz, $CDCl_3$) δ 145.0, 144.9, 142.7, 138.4, 128.5, 128.4, 127.7, 127.3, 127.1, 126.6, 126.5, 113.8, 61.1, 59.9, 47.2, 40.0, 36.8, 33.3. **HRMS** (ESI/micrOTOF) *m/z*: $[M + Na]^+$ Calcd. for $C_{22}H_{26}NaO_2^+$ 345.1825, Found 345.1816.

(3*E*,7*E*)-3,8-diphenyl-dec-3,7-diene-1,10-diol (*ll*-10a)

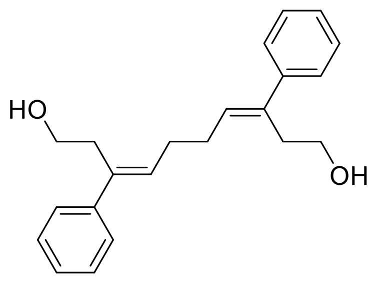

Using **L4**, *ll*-**10a** was obtained as a yellowish oil (21 mg, 0.071 mmol, 71%). **^{1}H NMR** (400 MHz, $CDCl_3$) δ 7.39 – 7.28 (m, 8H), 7.26 – 7.21 (m, 2H), 5.94 – 5.81 (m, 2H), 3.62 (t, *J* = 6.8 Hz, 4H), 2.83 (t, *J* = 6.8 Hz, 4H), 2.48 – 2.33 (m, 4H). ^{13}C NMR (101 MHz, $CDCl_3$) δ 142.5, 137.0, 130.8, 128.5, 127.1, 126.5, 61.4, 33.4, 29.3. **HRMS** (ESI/micrOTOF) *m/z*: $[M + Na]^+$ Calcd. for $C_{22}H_{26}NaO_2^+$ 345.1825; Found 345.1811.

7. X-ray molecular structure of **2j** (CCDC-2553598)

Experimental. Compound **2j** was recrystallized from MeCN (reflux to r.t. then fridge, heating mantle). Single crystals of **2j** were used as supplied. A suitable crystal was selected and mounted on a **XtaLAB AFC11 (RCD3): quarter-chi single** diffractometer. The crystal was kept at 100.00(10) K during data collection. Using Olex2,[22] the structure was solved with the SHELXT[23] structure solution program using Intrinsic Phasing and refined with the SHELXL [3][24] refinement package using Least Squares minimisation.

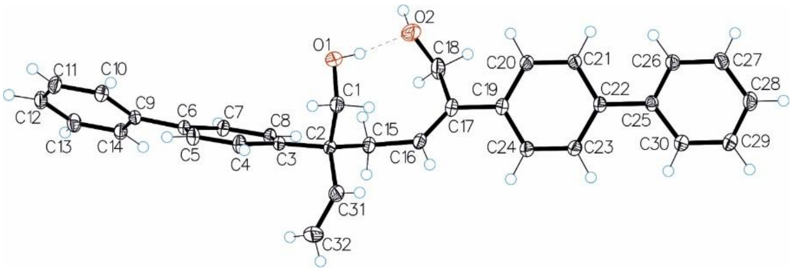

Figure S6. Molecular structure of **2j** with an adopted numbering scheme and ellipsoid contour probability at 50%.

Table S7. Crystal data and structure refinement for **2j**

Empirical formula	$C_{32}H_{30}O_2$	a/Å	10.1771(3)
Formula weight	446.56	b/Å	10.3585(2)
Temperature/K	100.00(10)	c/Å	12.1463(4)
Crystal system	triclinic	α/°	100.182(2)
Space group	P-1	β/°	112.175(3)
γ/°		90.090(2)	
Volume/Å^3		1163.81(6)	
Z		2	
ρ_{calc}g/cm^3		1.274	
μ/mm^{-1}		0.078	
F(000)		476.0	
Crystal size/mm^3		0.6 × 0.3 × 0.2	
Radiation		Mo Kα (λ = 0.71073)	
2Θ range for data collection/°		4.006 to 64.948	
Index ranges		-15 ≤ h ≤ 11, -15 ≤ k ≤ 15, -18 ≤ l ≤ 18	
Reflections collected		23169	
Independent reflections		7877 [R_{int} = 0.0273, R_{sigma} = 0.0342]	
Data/restraints/parameters		7877/0/316	
Goodness-of-fit on F^2		1.035	
Final R indexes [I>=2σ (I)]		R_1 = 0.0511, wR_2 = 0.1375	
Final R indexes [all data]		R_1 = 0.0667, wR_2 = 0.1463	
Largest diff. peak/hole / e Å^{-3}		0.51/-0.29	

(22) Dolomanov, O. V.; Bourhis, L. J.; Gildea, R. J.; Howard, J. A. K.; Puschmann, H. *J. Appl. Cryst*. **2009**, *42*, 339-341.
(23) Sheldrick, G.M. *Acta Cryst*. **2015**, *A71*, 3-8.
(24) Sheldrick, G.M. *Acta Cryst*. **2015**, *C71*, 3-8.

8. NMR spectra for all new compounds

2,2'-((methylphosphanediyl)bis(2,1-phenylene))bis(4,5-dihydrooxazole) (L5 with 10% phosphine oxide)

^{1}H NMR (400 MHz, $CDCl_3$)

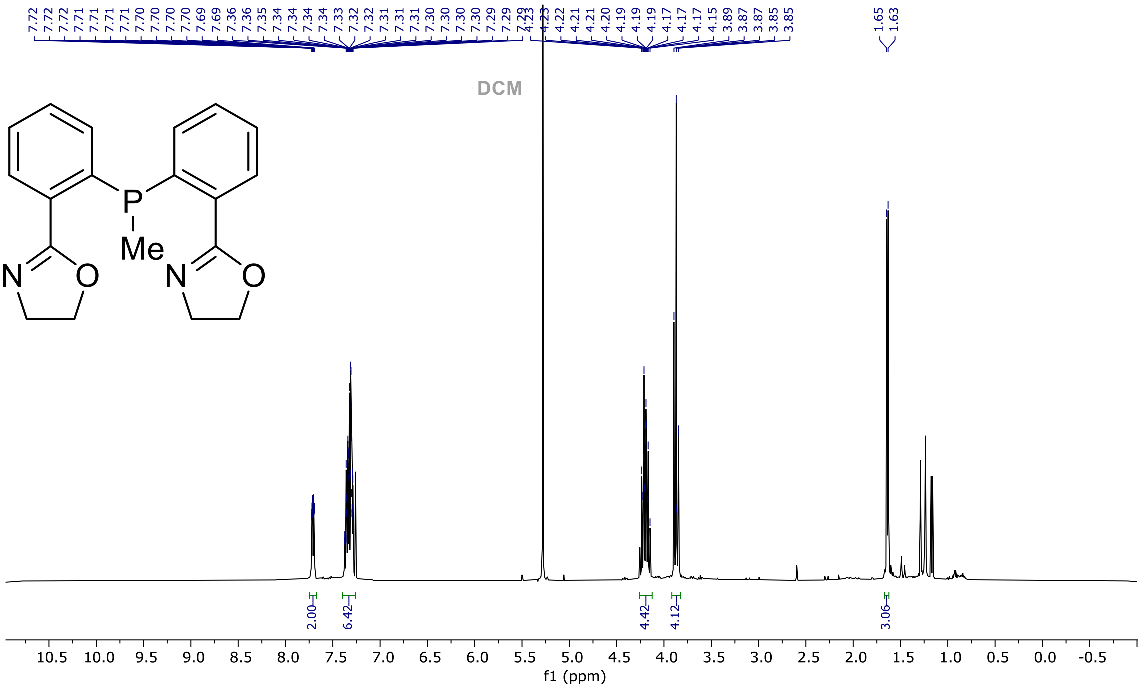

^{13}C NMR (101 MHz, $CDCl_3$) after 72 hours

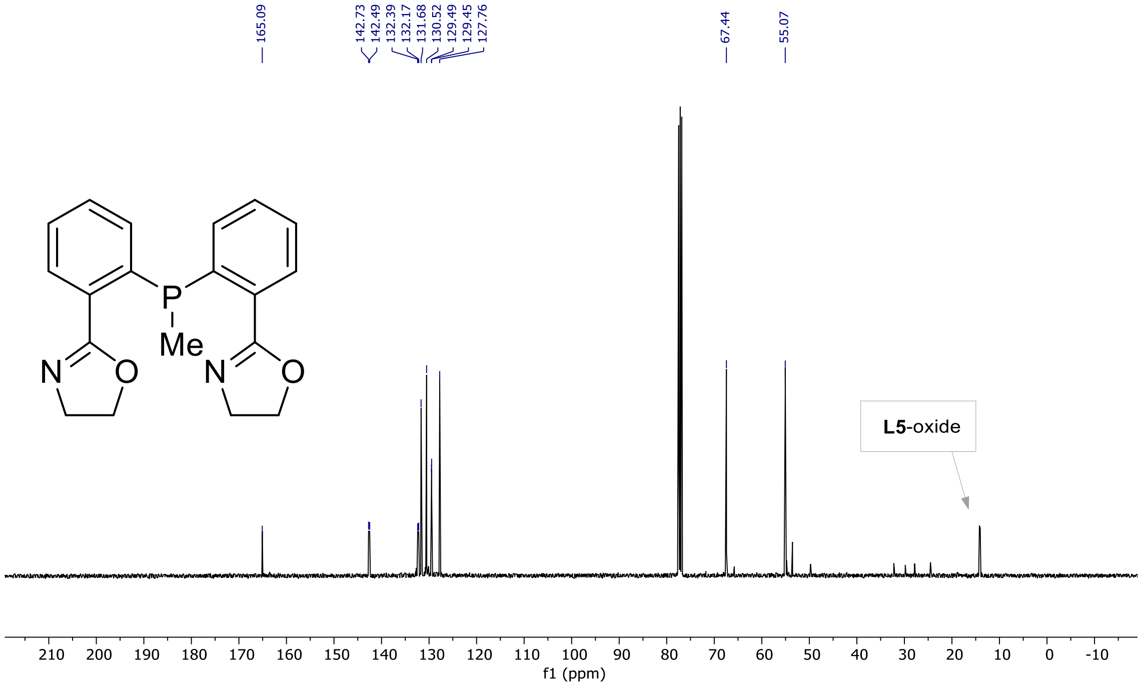

^{31}P NMR (162 MHz, $CDCl_3$)

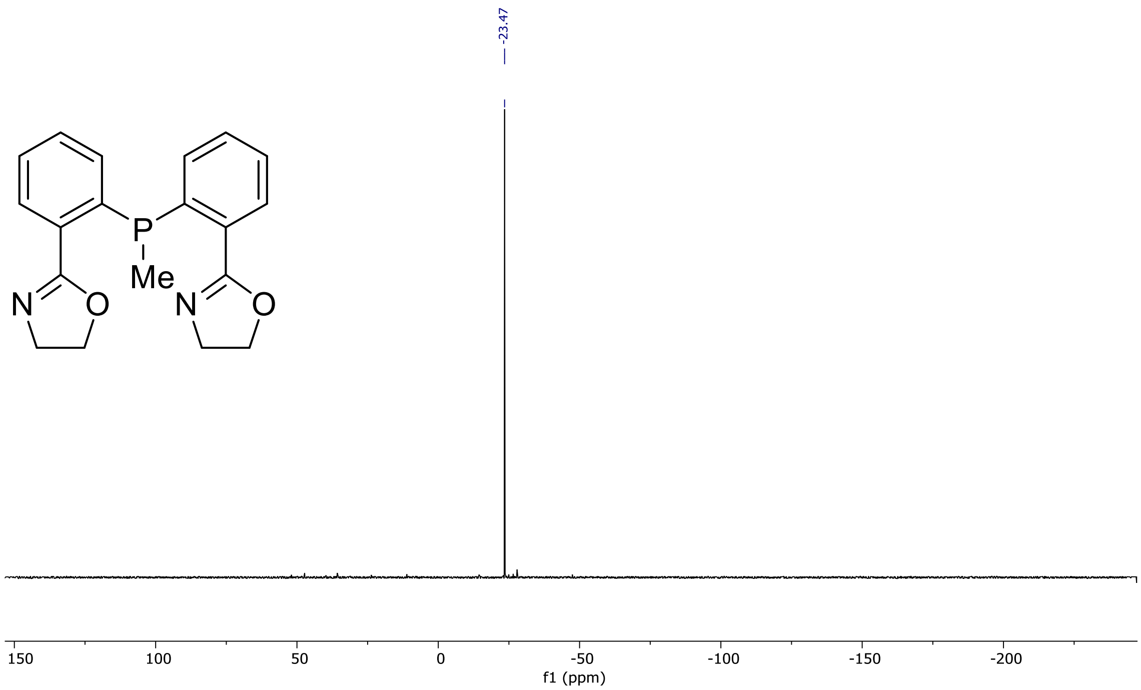

^{31}P NMR (162 MHz, $CDCl_3$) after 72 hours

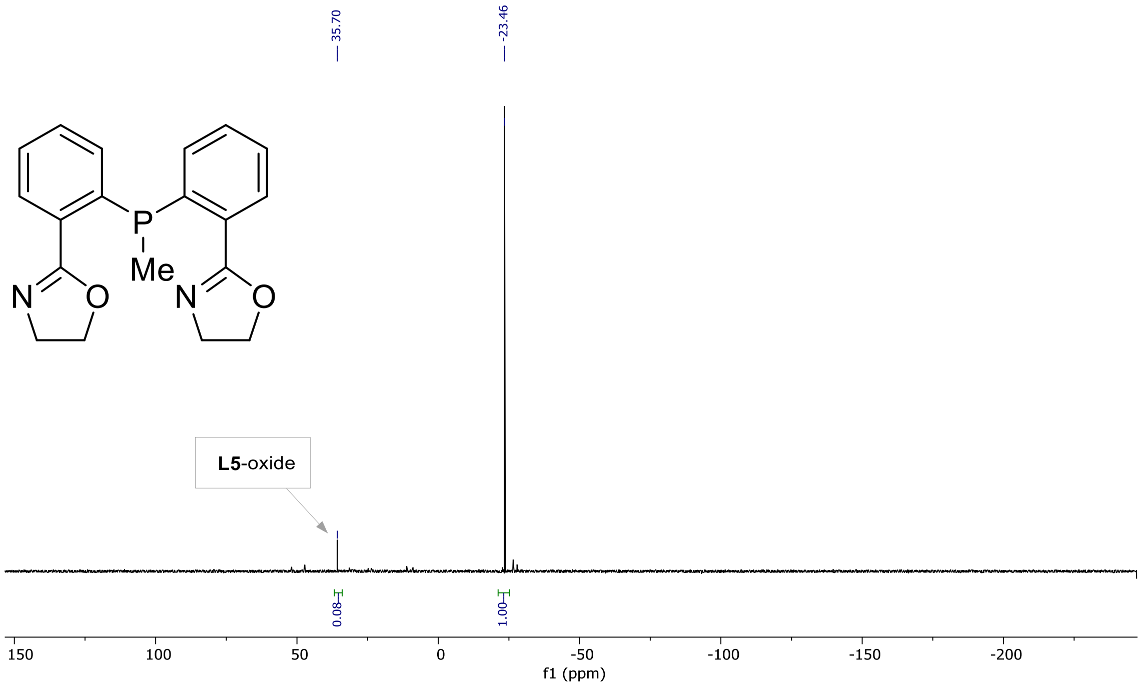

2,2'-((methylphosphanediyl)bis(5-methoxy-2,1-phenylene))bis(4,5-dihydrooxazole) (L6)

^{1}H NMR (400 MHz, $CDCl_3$)

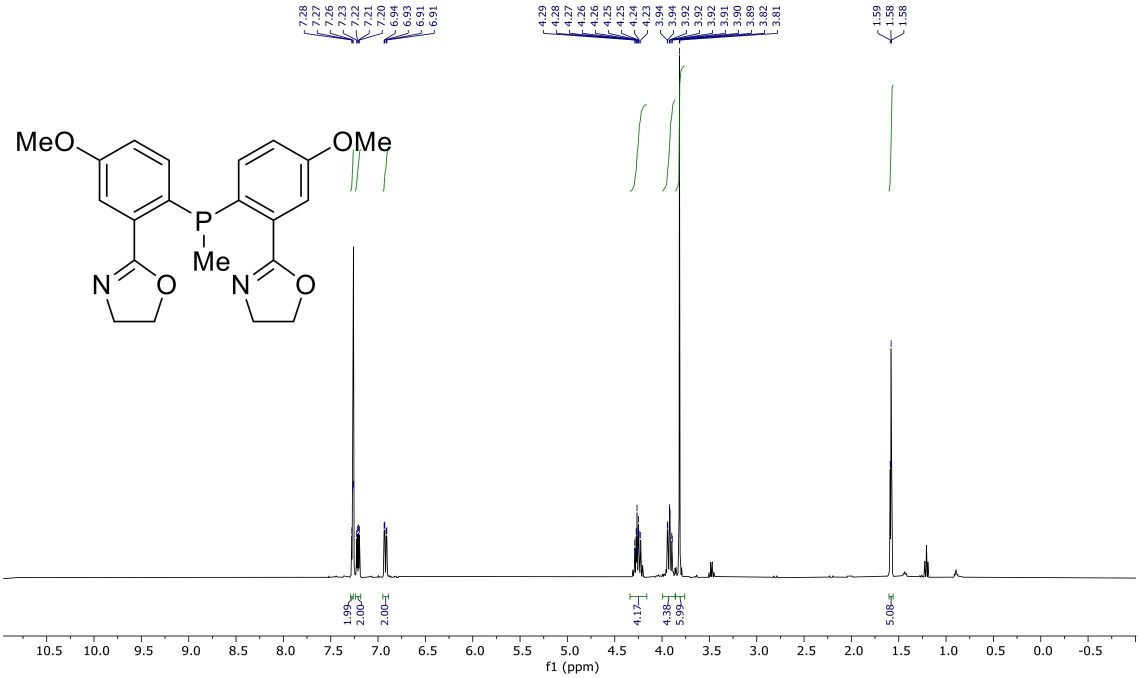

^{13}C NMR (101 MHz, $CDCl_3$)

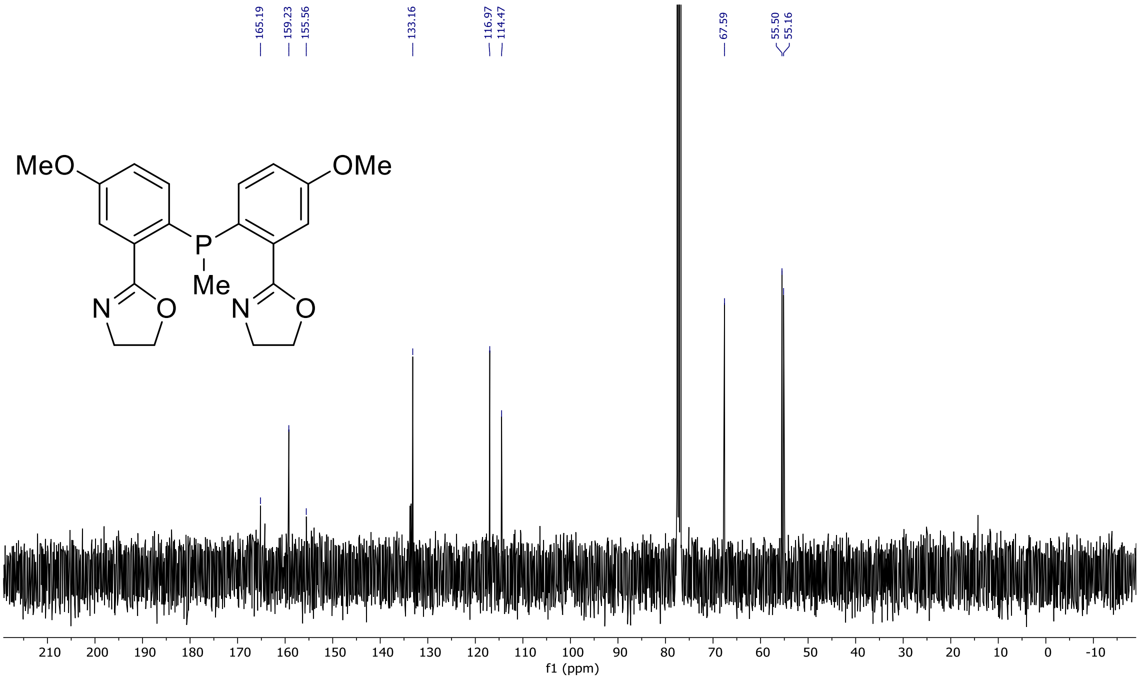

^{31}P NMR (162 MHz, $CDCl_3$)

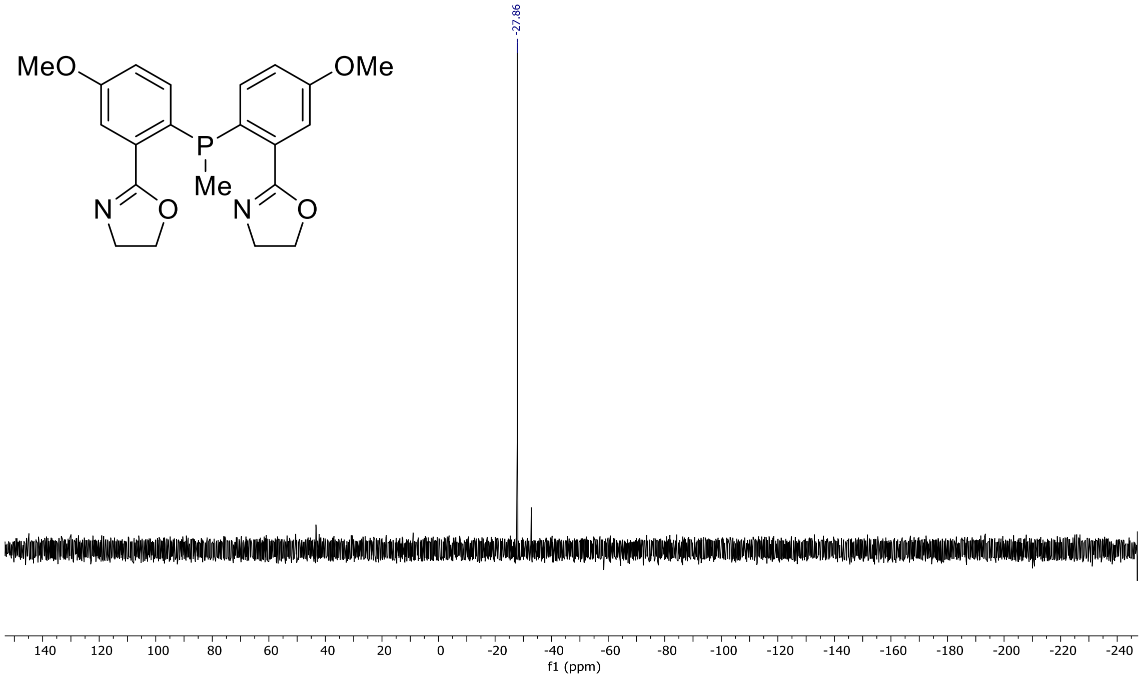

4-(3-benzyloxyphenyl)-4-vinyl-1,3-dioxolan-2-one (1m)

^{1}H NMR (400 MHz, $CDCl_3$)

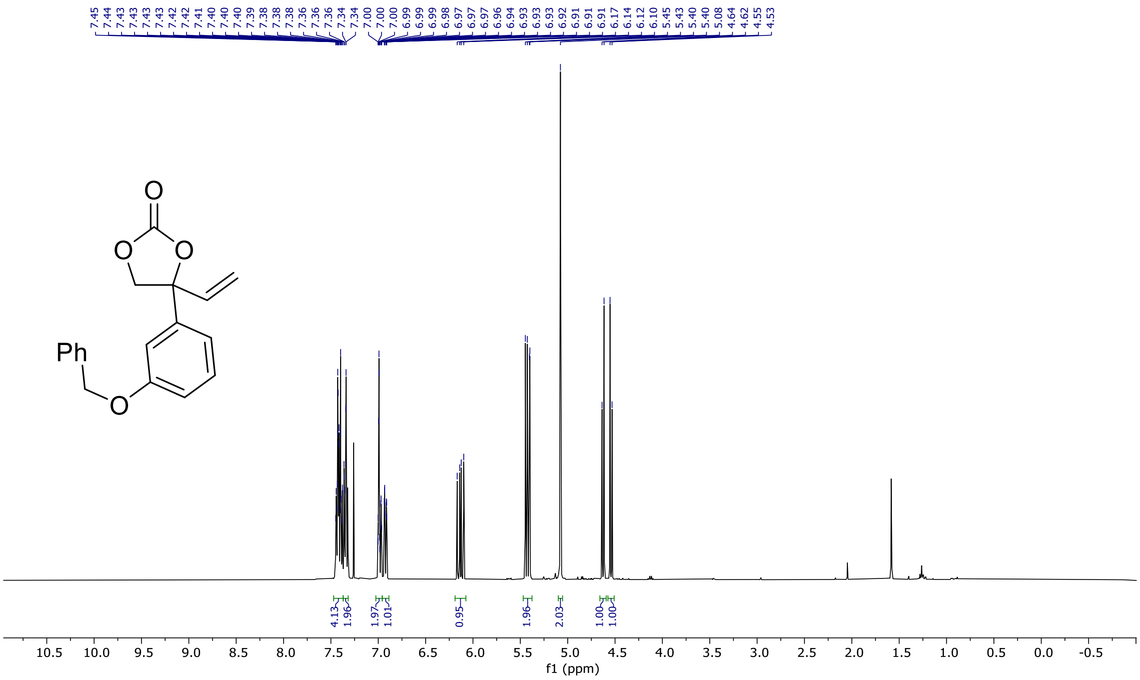

^{13}C NMR (101 MHz, $CDCl_3$)

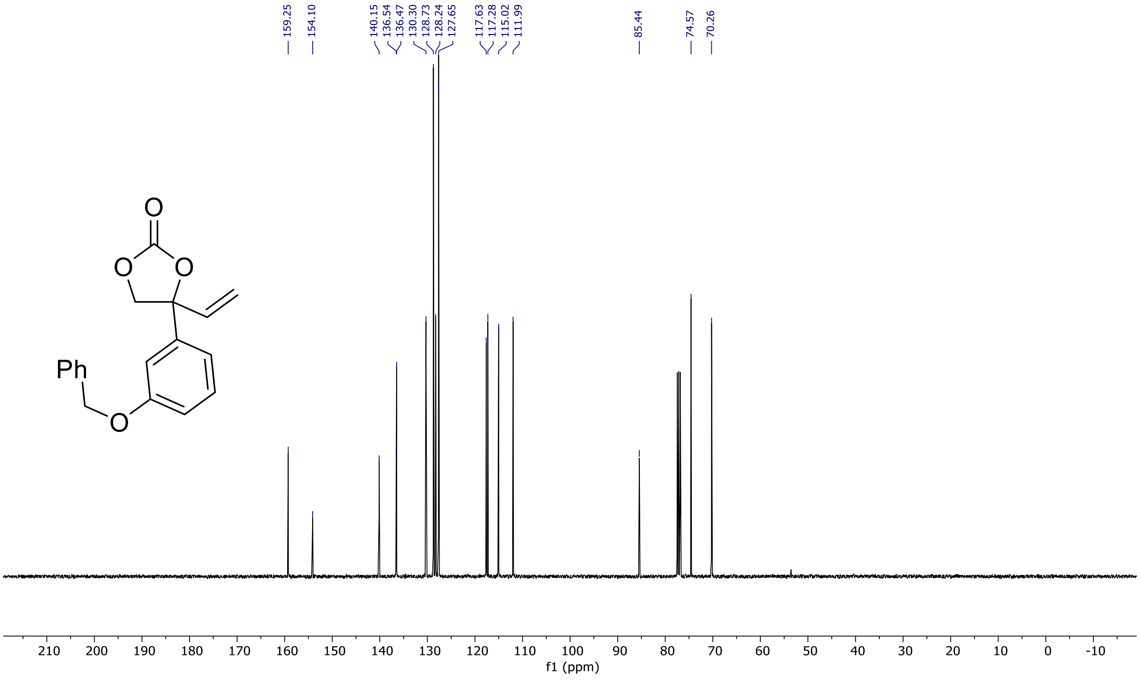

4-(3,5-dimethoxyphenyl)-4-vinyl-1,3-dioxolan-2-one (1s)

^{1}H NMR (400 MHz, $CDCl_3$)

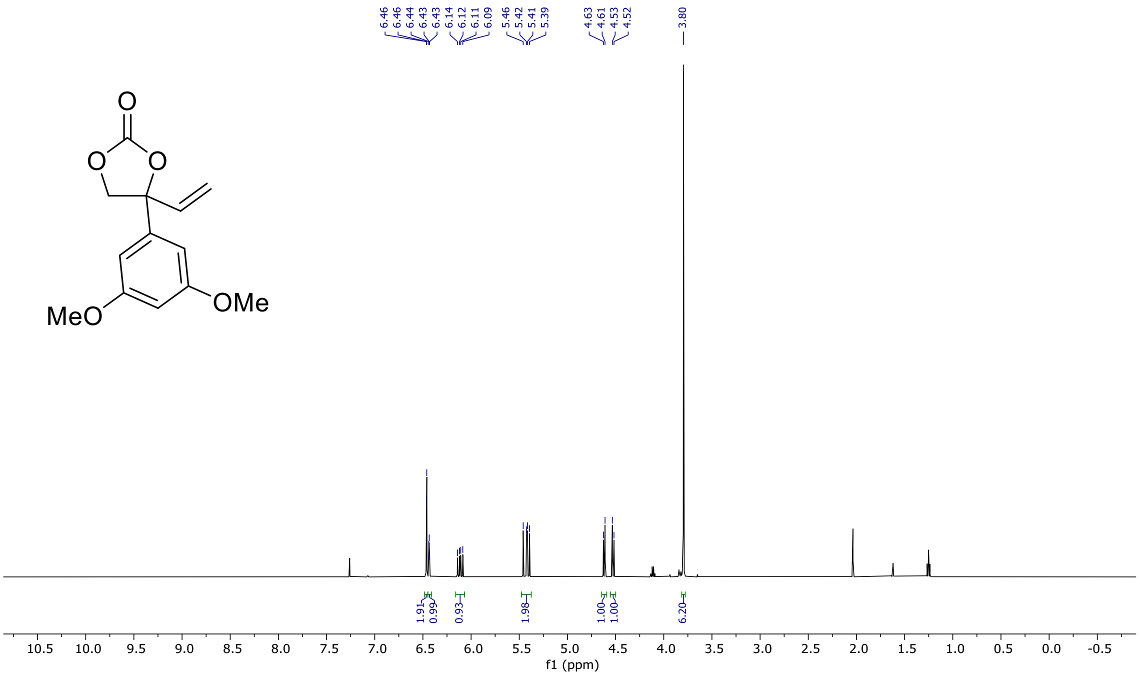

^{13}C NMR (101 MHz, $CDCl_3$)

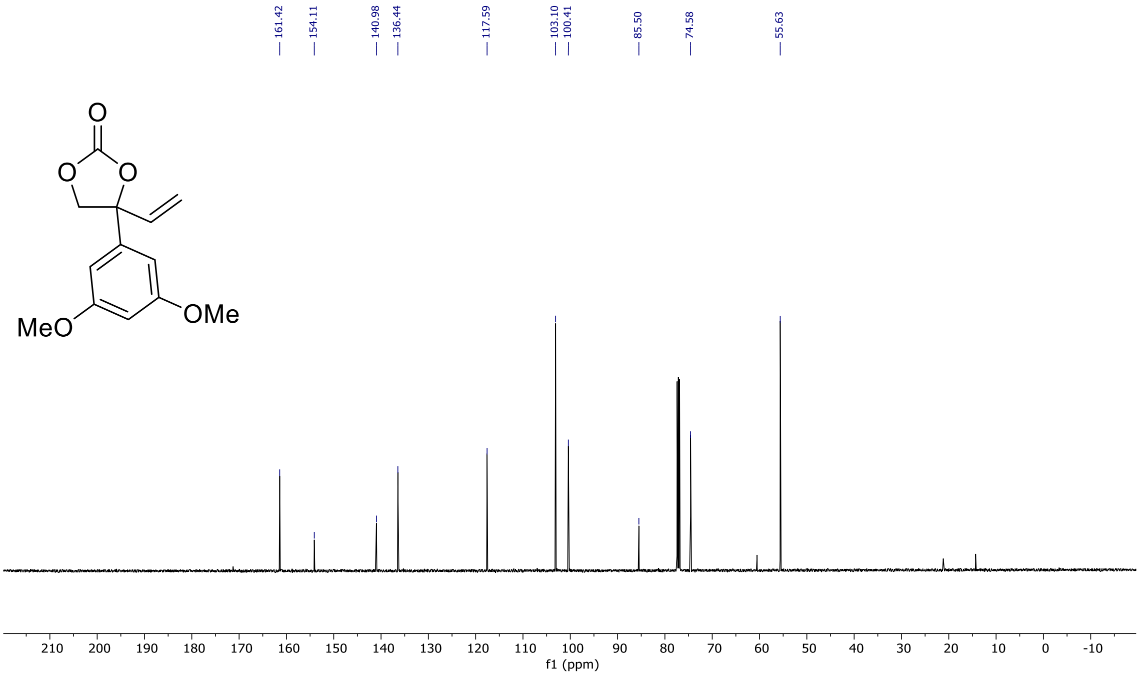

(*Z*)-2,5-diphenyl-5-vinylhex-2-ene-1,6-diol (*bl*-2a)

^{1}H NMR (400 MHz, $CDCl_3$)

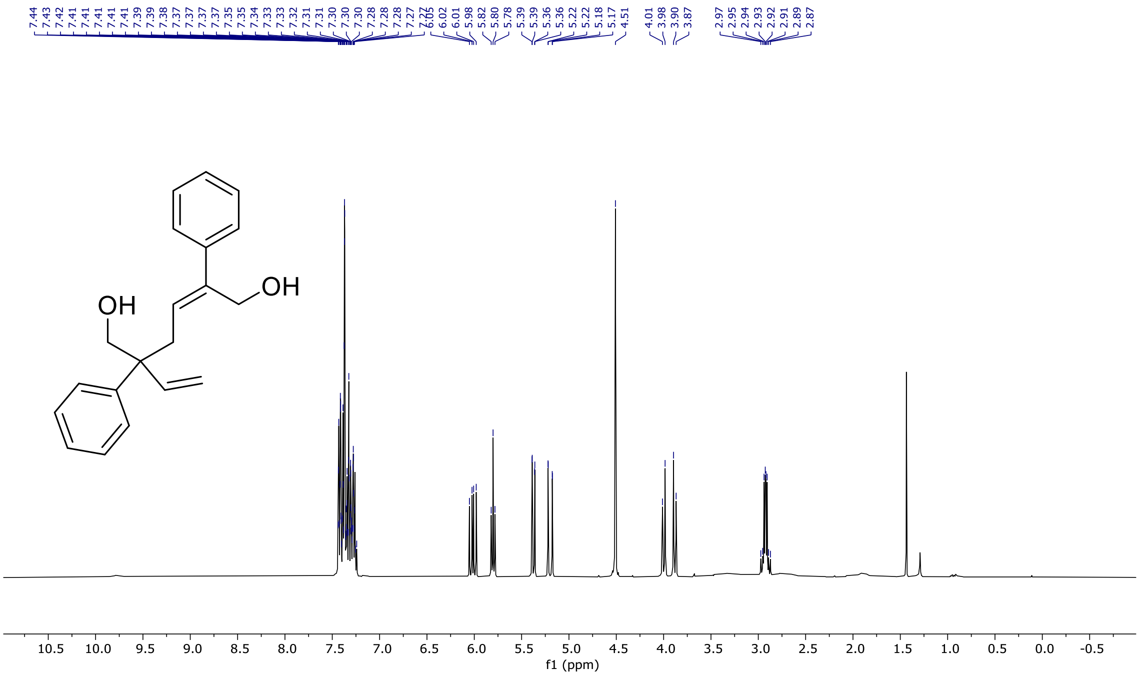

^{13}C NMR (101 MHz, $CDCl_3$)

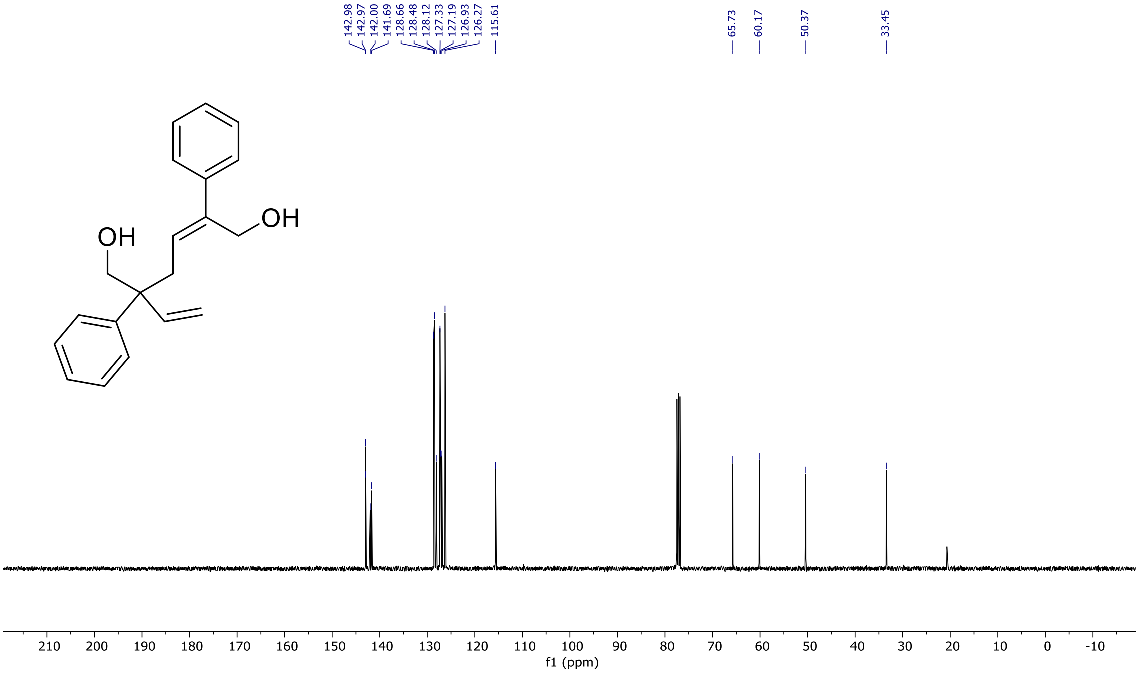

(2*Z*,6*Z*)-2,7-diphenylocta-2,6-diene-1,8-diol (//-2a)

^{1}H NMR (400 MHz, $CDCl_3$)

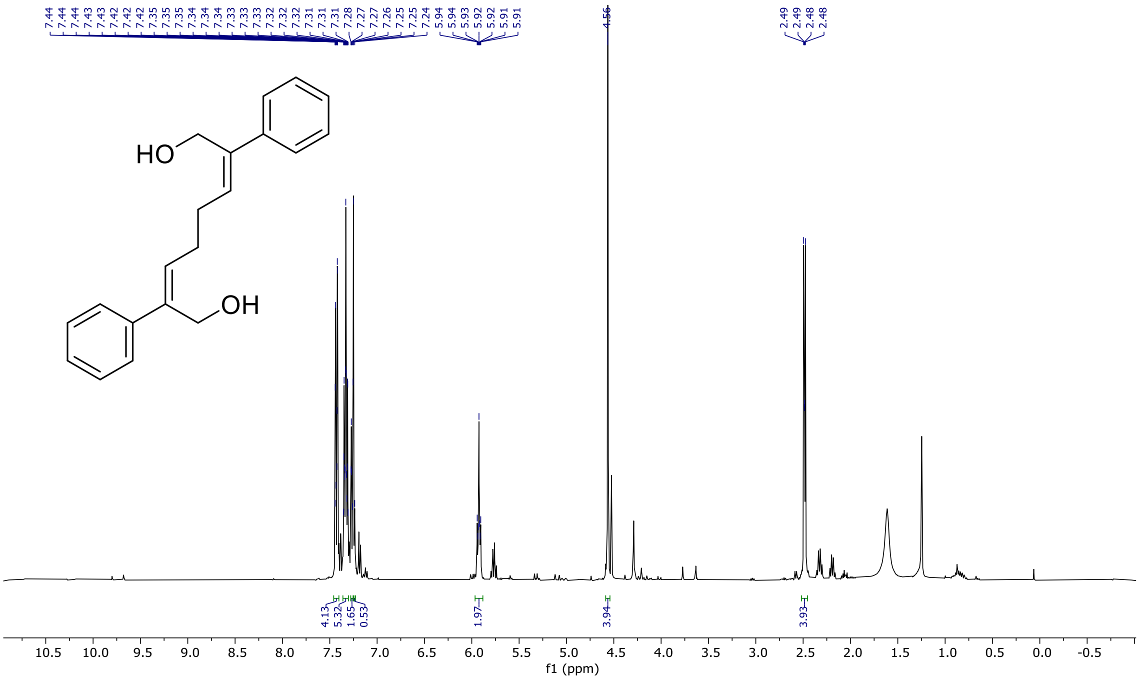

^{13}C NMR (101 MHz, $CDCl_3$)

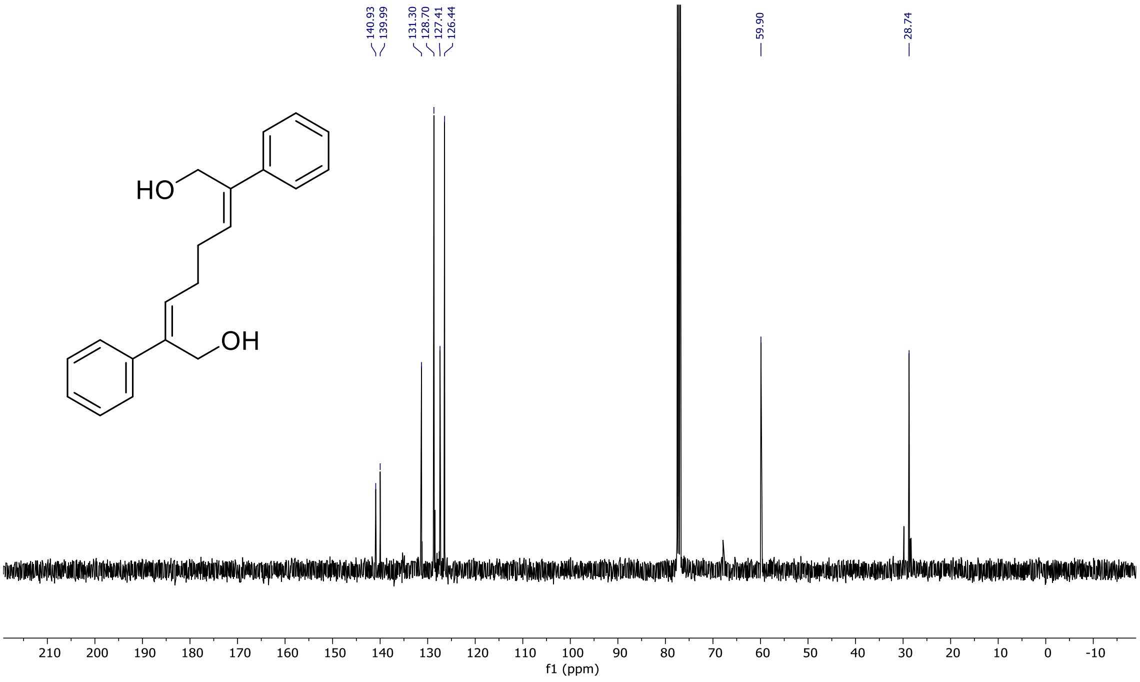

(*Z*)-2,5-di-*p*-tolyl-5-vinylhex-2-ene-1,6-diol (2b)

^{1}H NMR (400 MHz, $CDCl_3$)

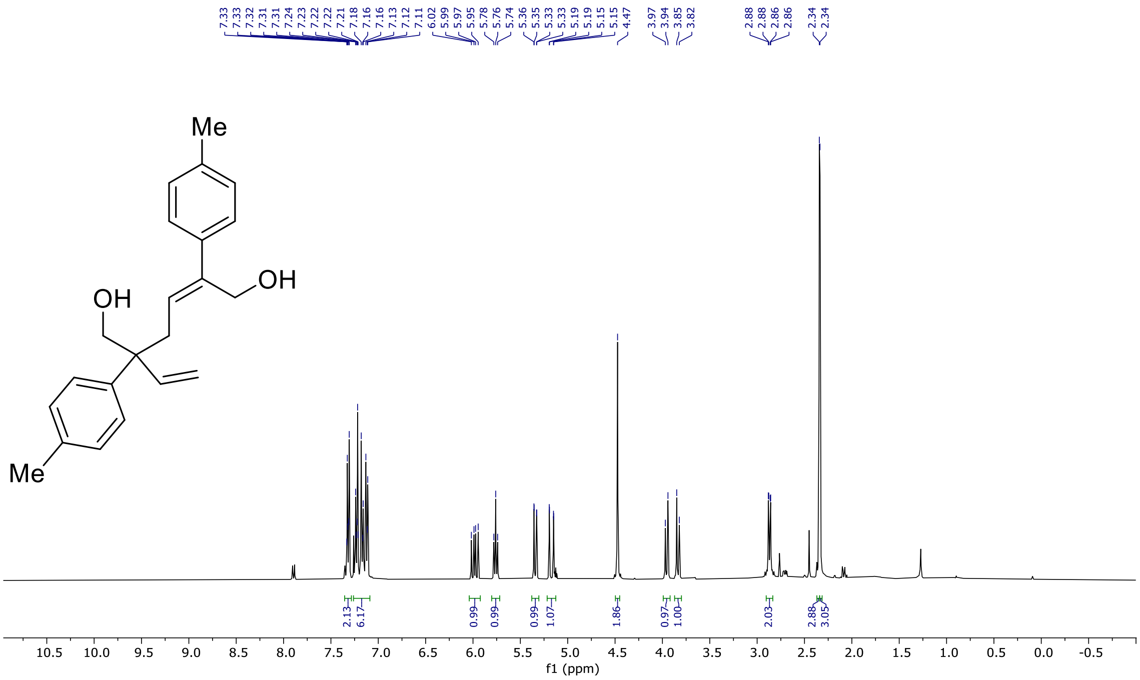

^{13}C NMR (101 MHz, $CDCl_3$)

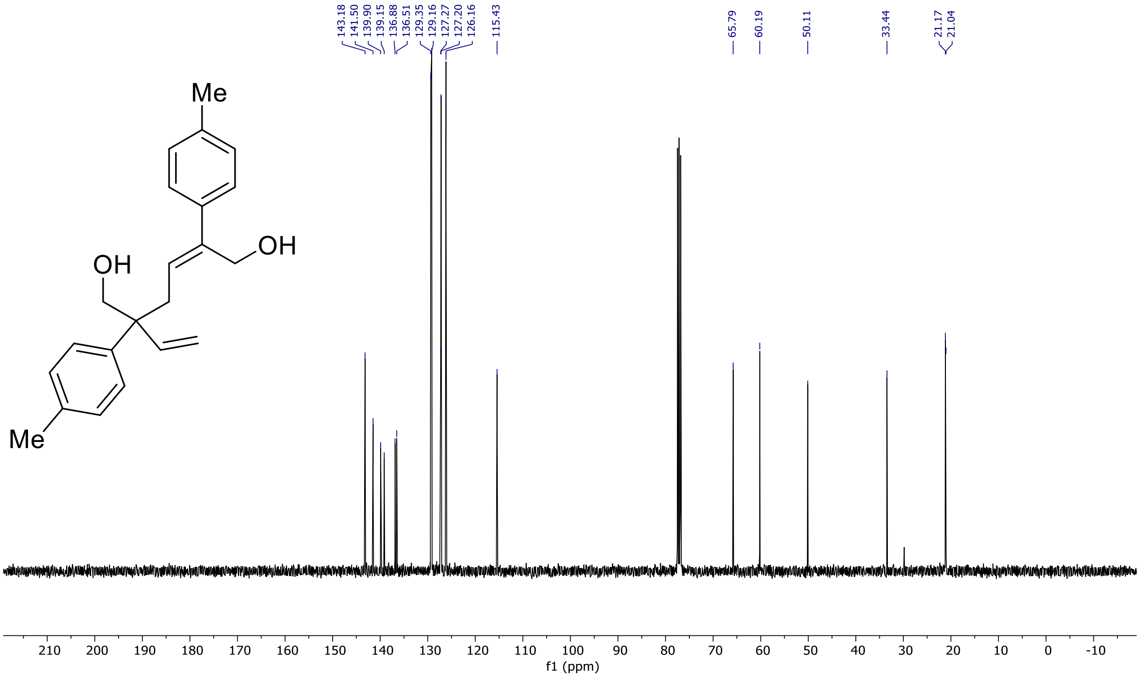

(*Z*)-2,5-bis(4-(*tert*-butyl)phenyl)-5-vinylhex-2-ene-1,6-diol (2c)

^{1}H NMR (400 MHz, $CDCl_3$)

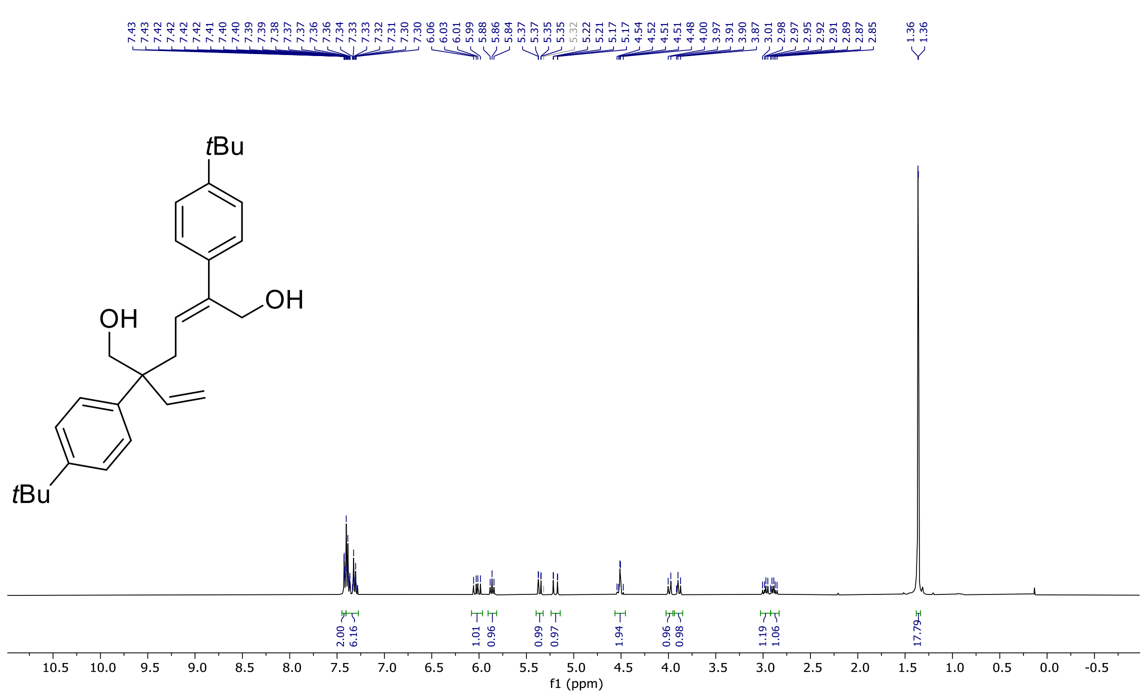

^{13}C NMR (101 MHz, $CDCl_3$)

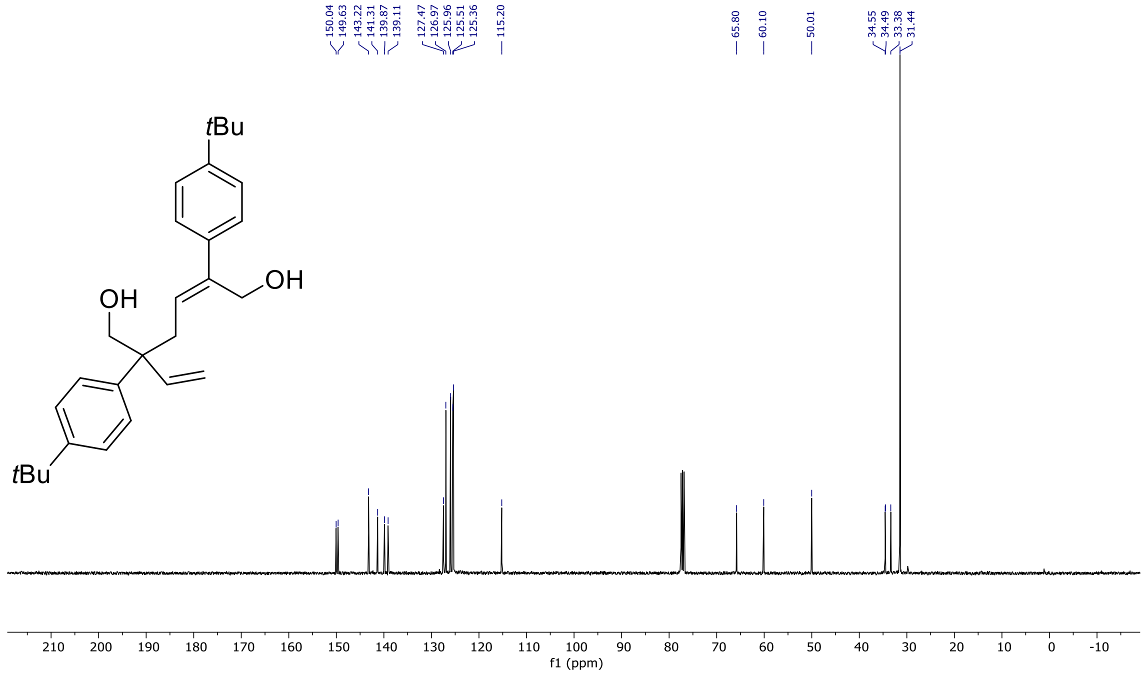

(*Z*)-2,5-bis(4-fluorophenyl)-5-vinylhex-2-ene-1,6-diol (2d)

^{1}H NMR (400 MHz, $CDCl_3$)

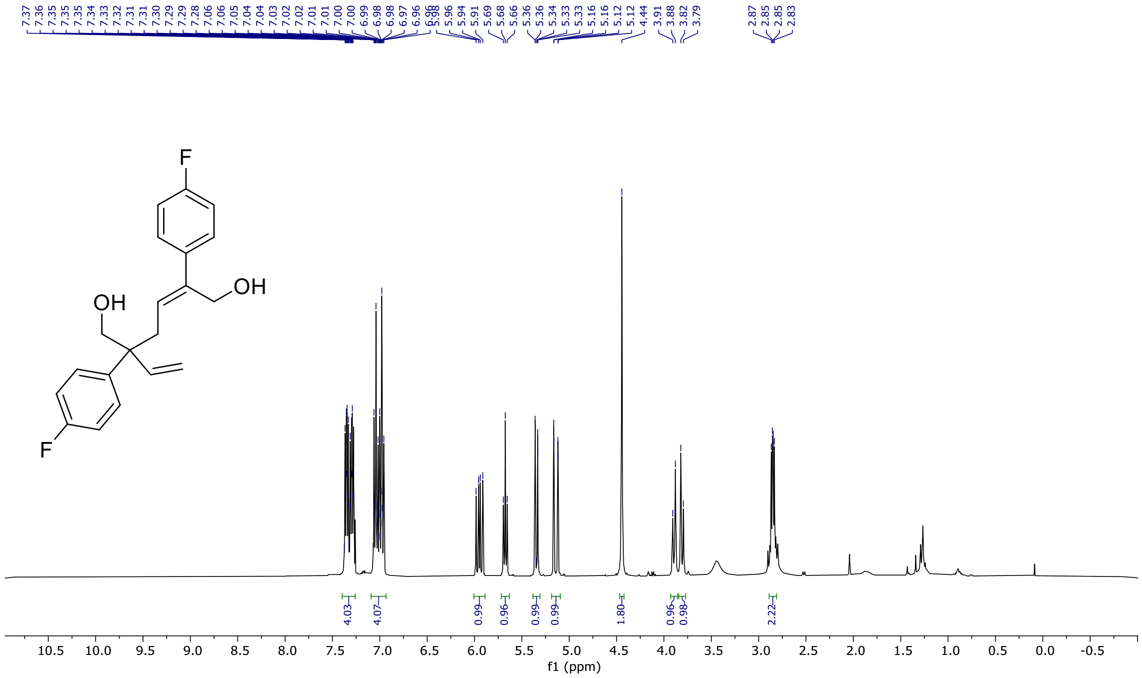

^{13}C NMR (101 MHz, $CDCl_3$)

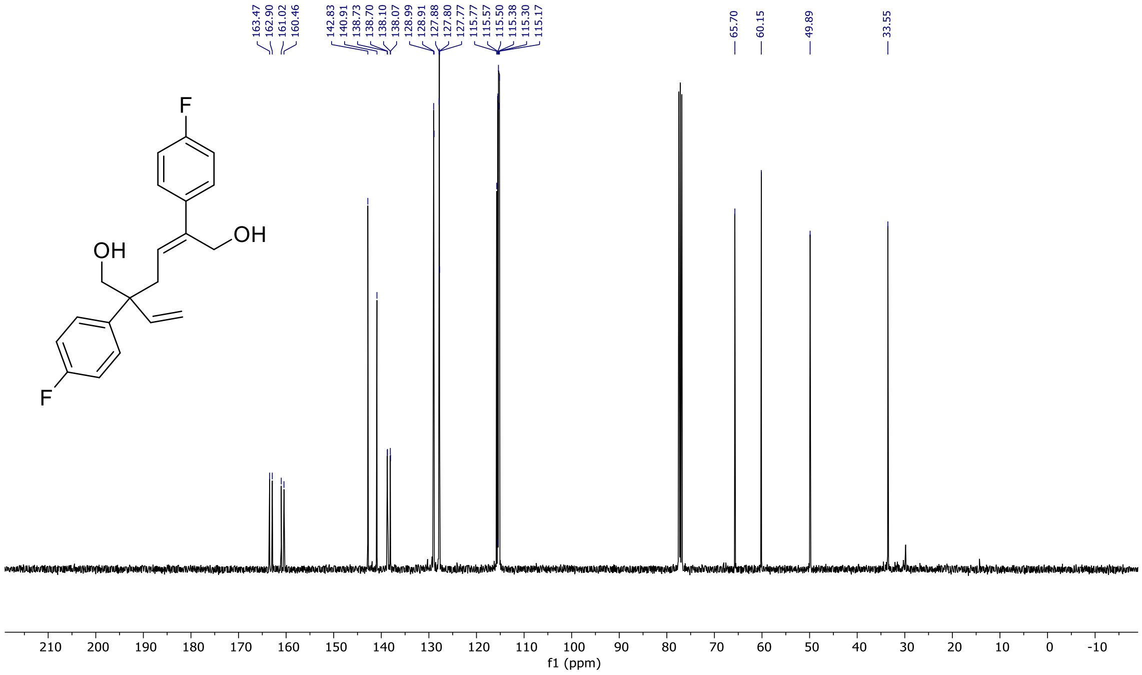

^{19}F NMR (376 MHz, CDCl$_3$)

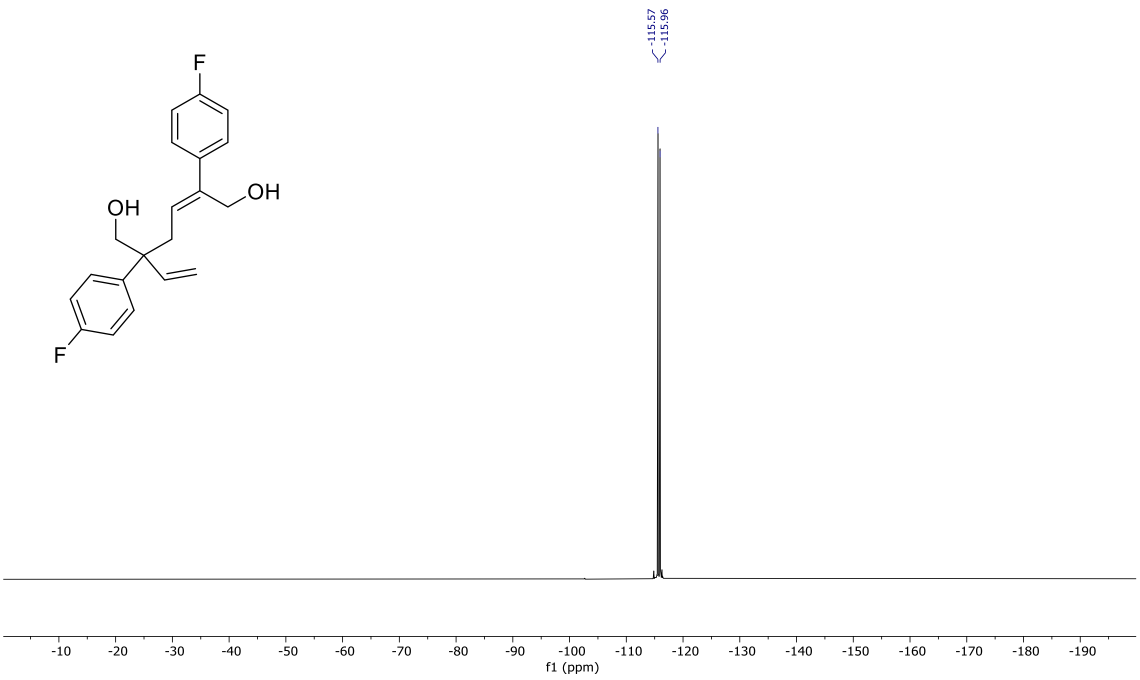

(*Z*)-2,5-bis(4-chlorophenyl)-5-vinylhex-2-ene-1,6-diol (2e)

^{1}H NMR (400 MHz, $CDCl_3$)

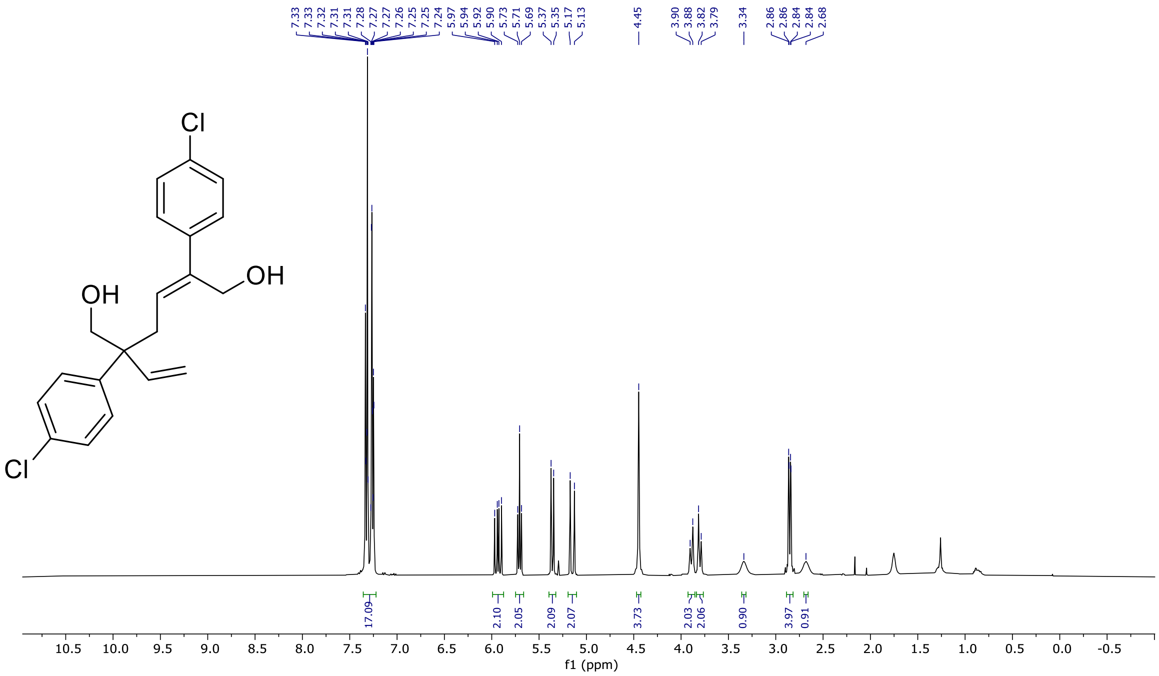

^{13}C NMR (101 MHz, $CDCl_3$)

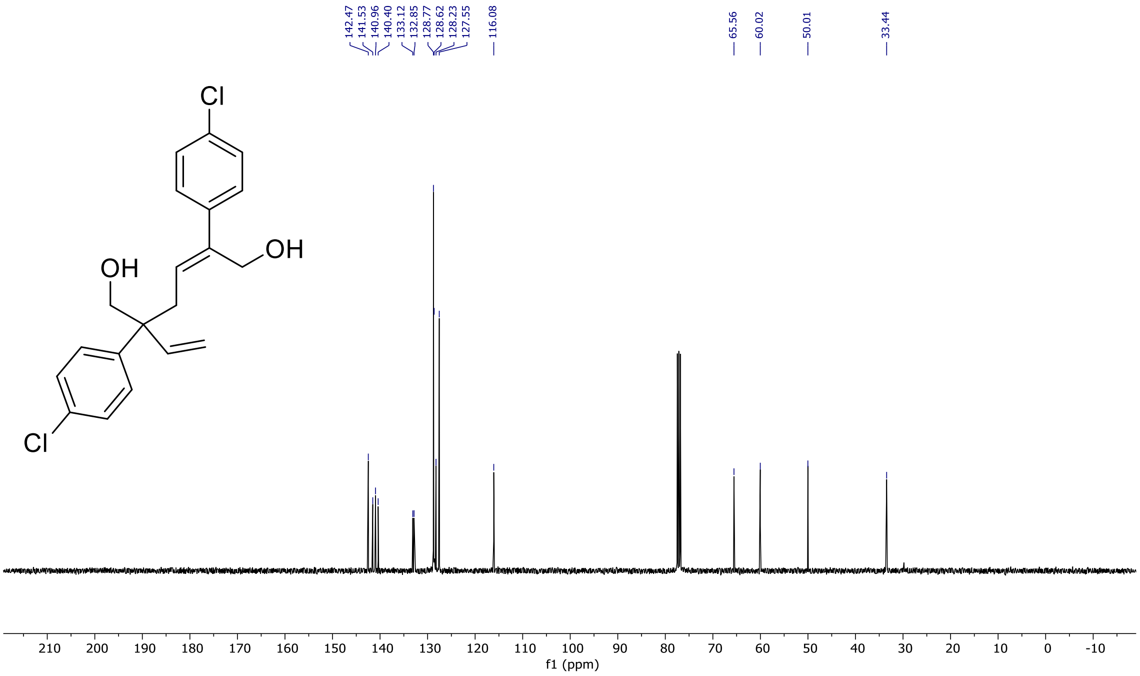

(*Z*)-2,5-bis(4-bromophenyl)-5-vinylhex-2-ene-1,6-diol (2f)

^{1}H NMR (400 MHz, $CDCl_3$)

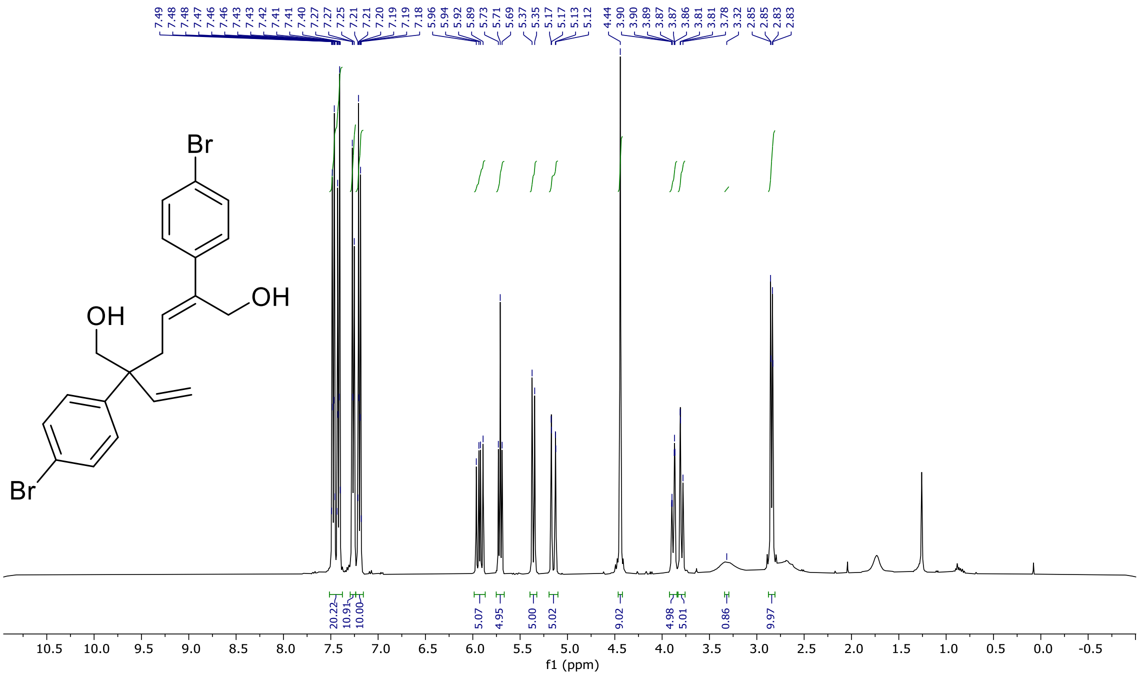

^{13}C NMR (101 MHz, $CDCl_3$)

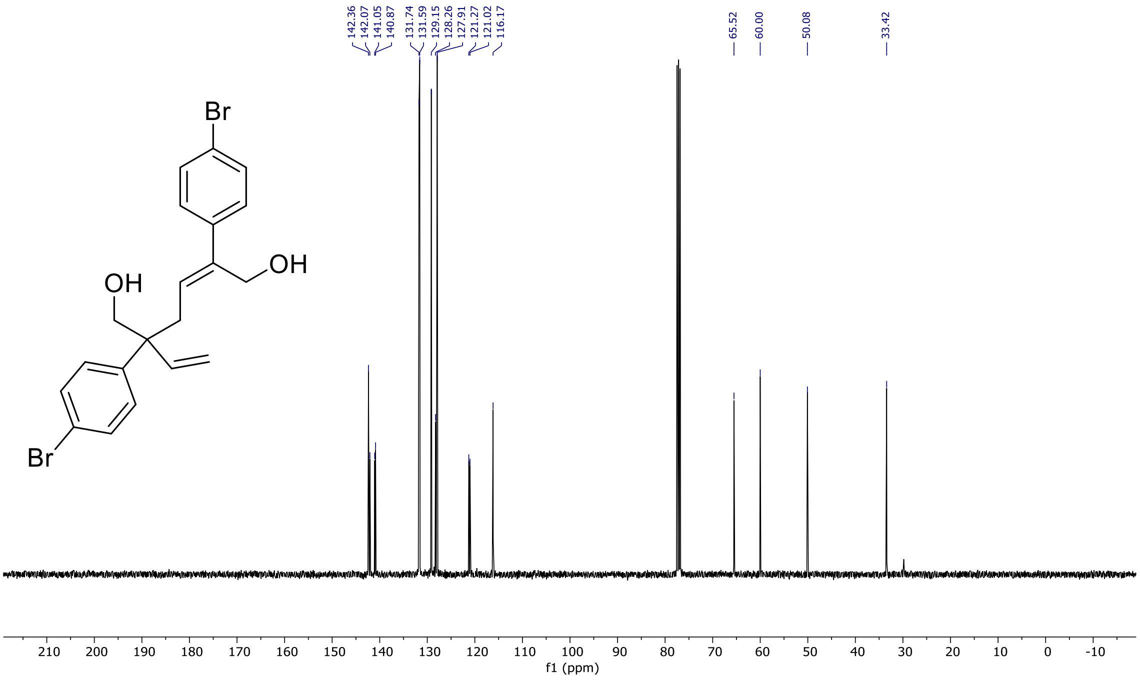

(*Z*)-2,5-bis(4-methoxyphenyl)-5-vinylhex-2-ene-1,6-diol (2g)

^{1}H NMR (400 MHz, $CDCl_3$)

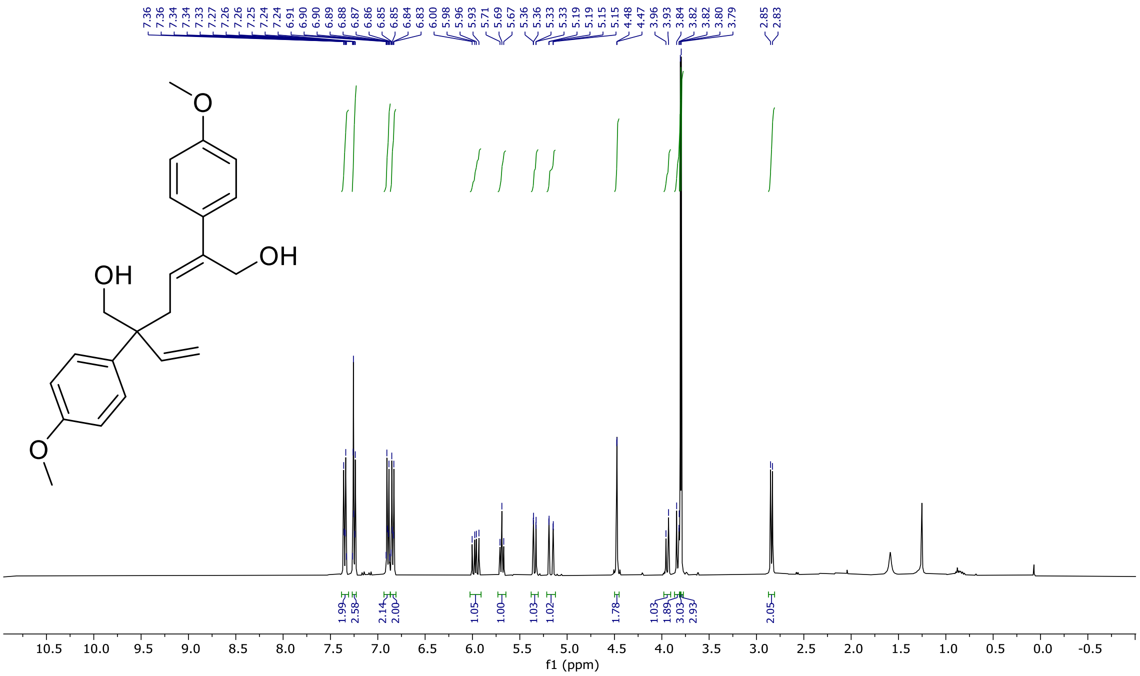

^{13}C NMR (101 MHz, $CDCl_3$)

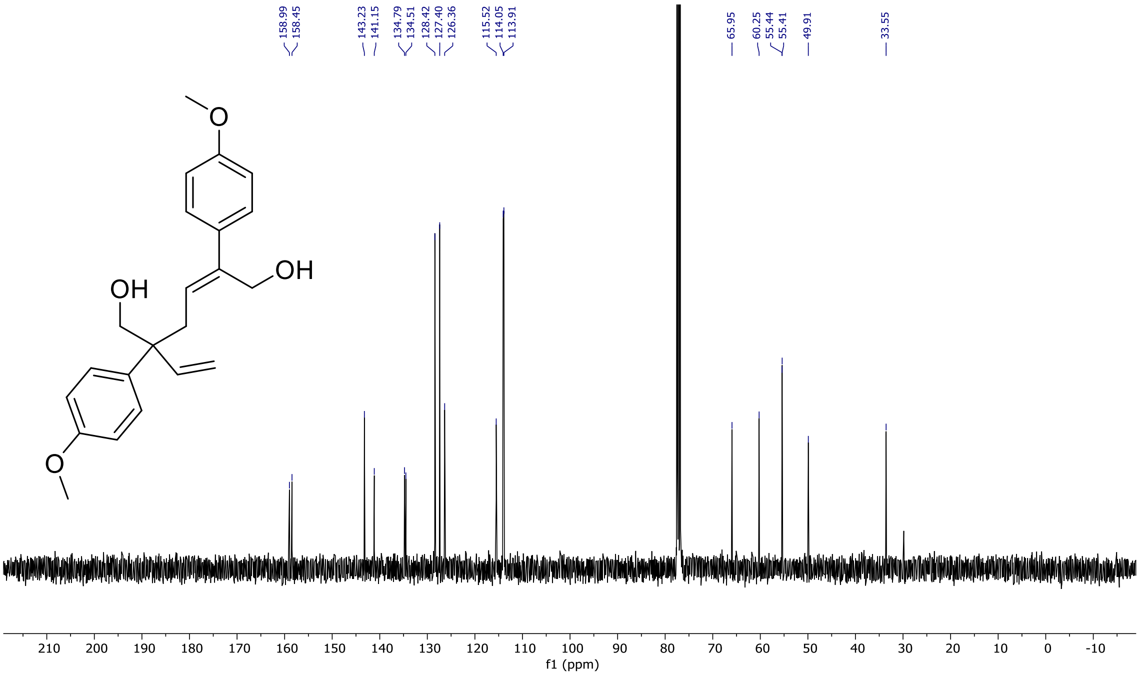

(*Z*)-2,5-bis(4-(benzyloxy)phenyl)-5-vinylhex-2-ene-1,6-diol (2h)

^{1}H NMR (400 MHz, $CDCl_3$)

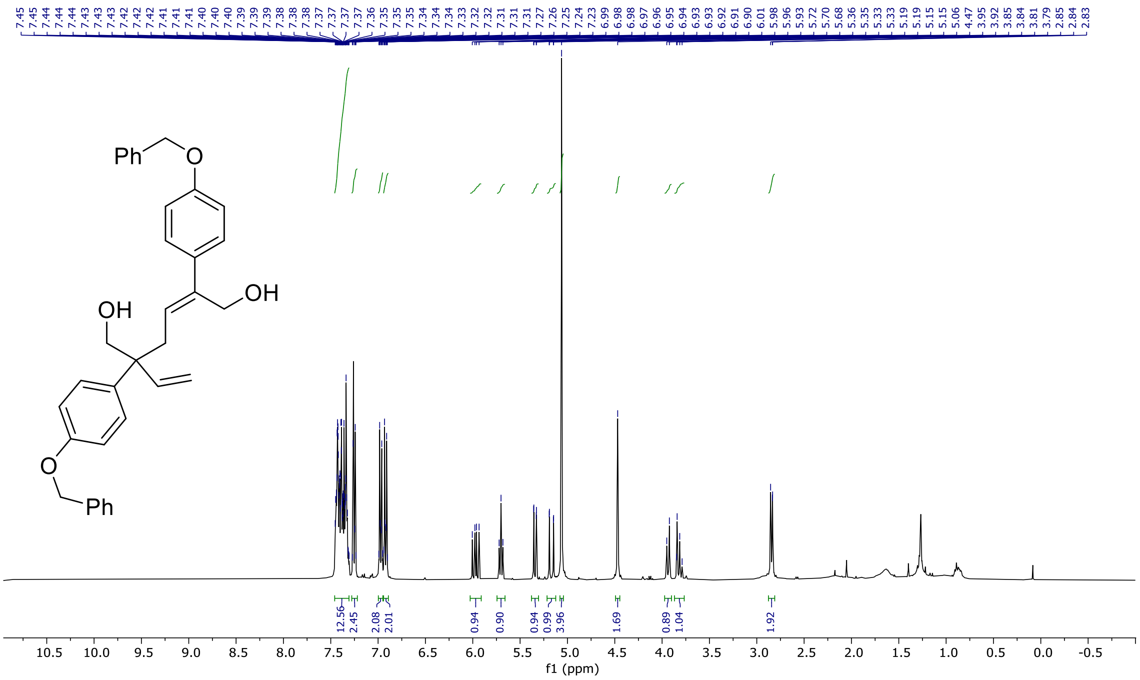

^{13}C NMR (101 MHz, $CDCl_3$)

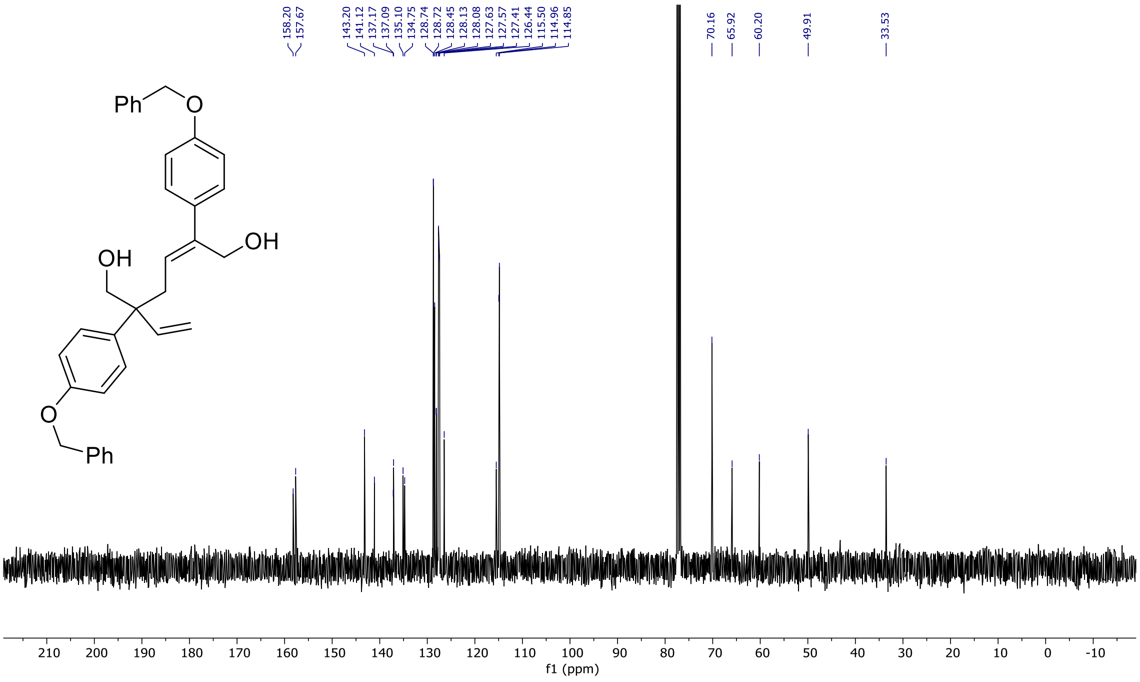

(*Z*)-2,5-bis(4-(trifluoromethyl)phenyl)-5-vinylhex-2-ene-1,6-diol (2i)

^{1}H NMR (400 MHz, $CDCl_3$)

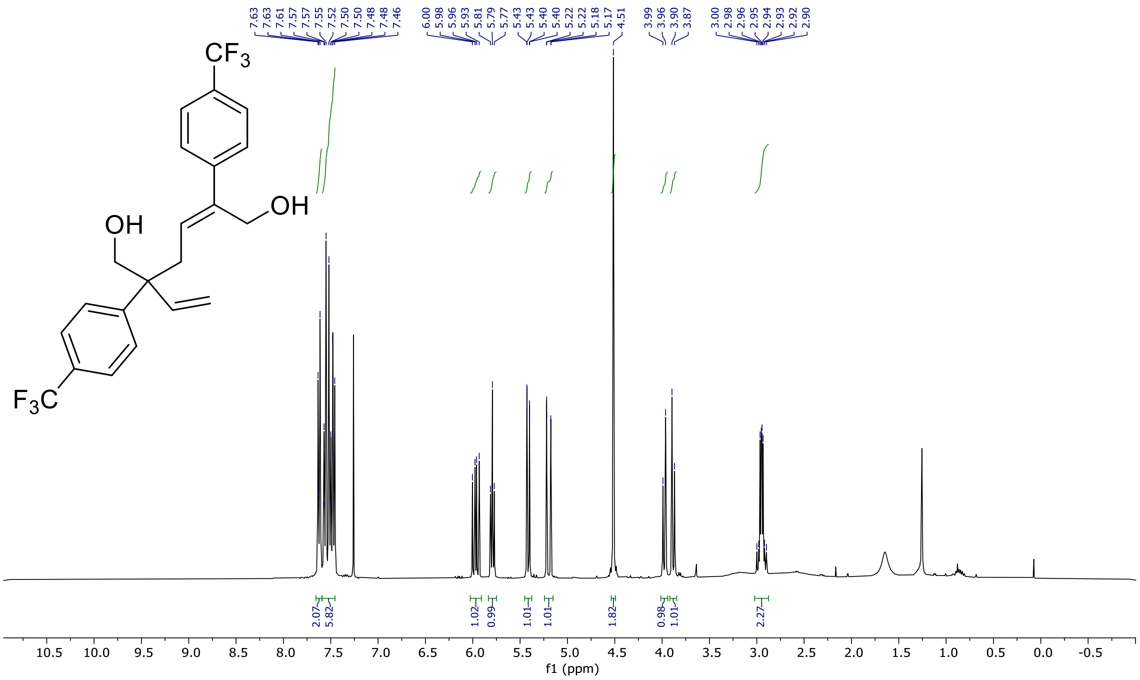

^{13}C NMR (101 MHz, $CDCl_3$)

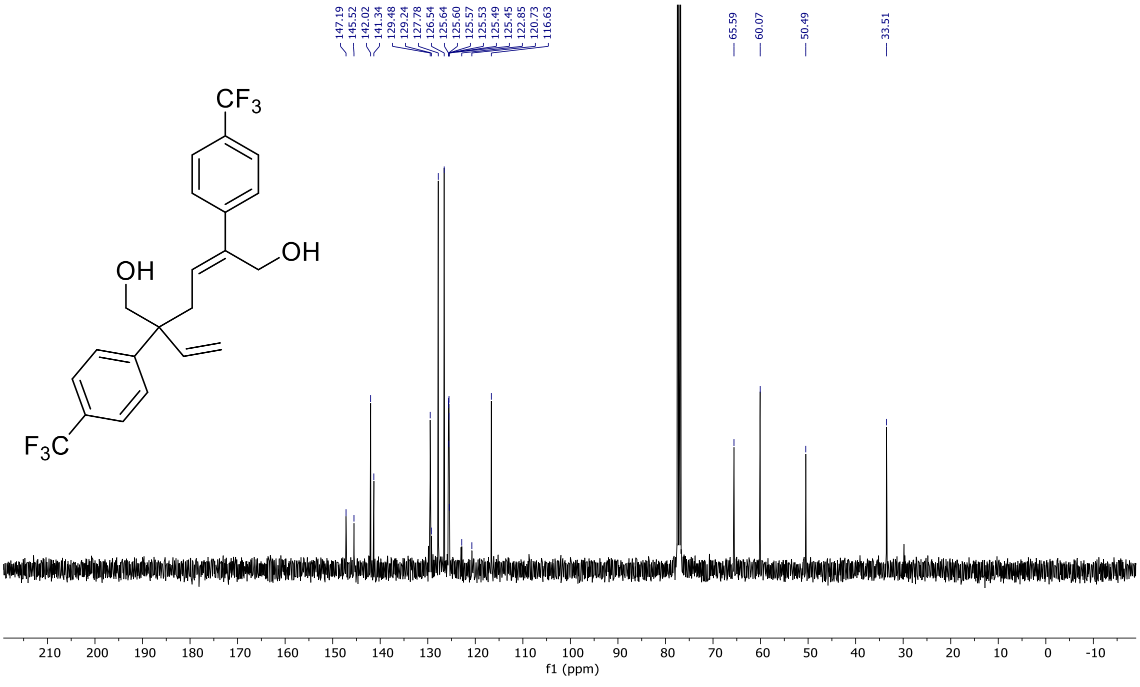

^{19}F NMR (376 MHz, $CDCl_3$)

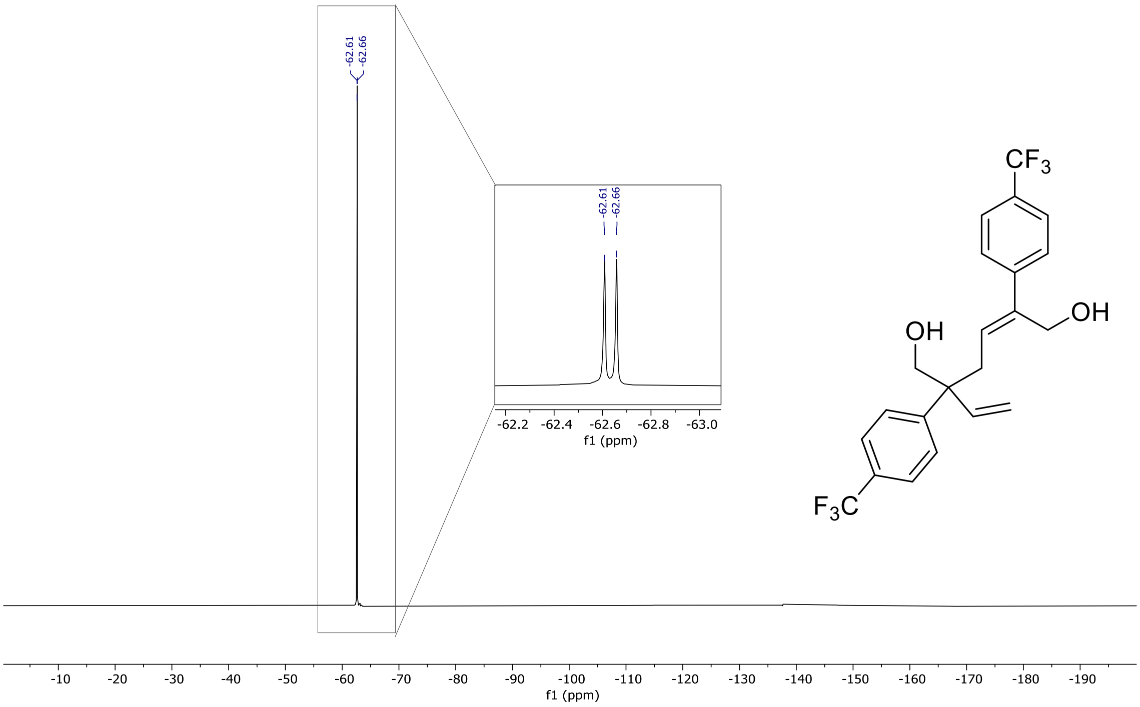

(*Z*)-2,5-di([1,1'-biphenyl]-4-yl)-5-vinylhex-2-ene-1,6-diol (2j)

^{1}H NMR (400 MHz, $CDCl_3$)

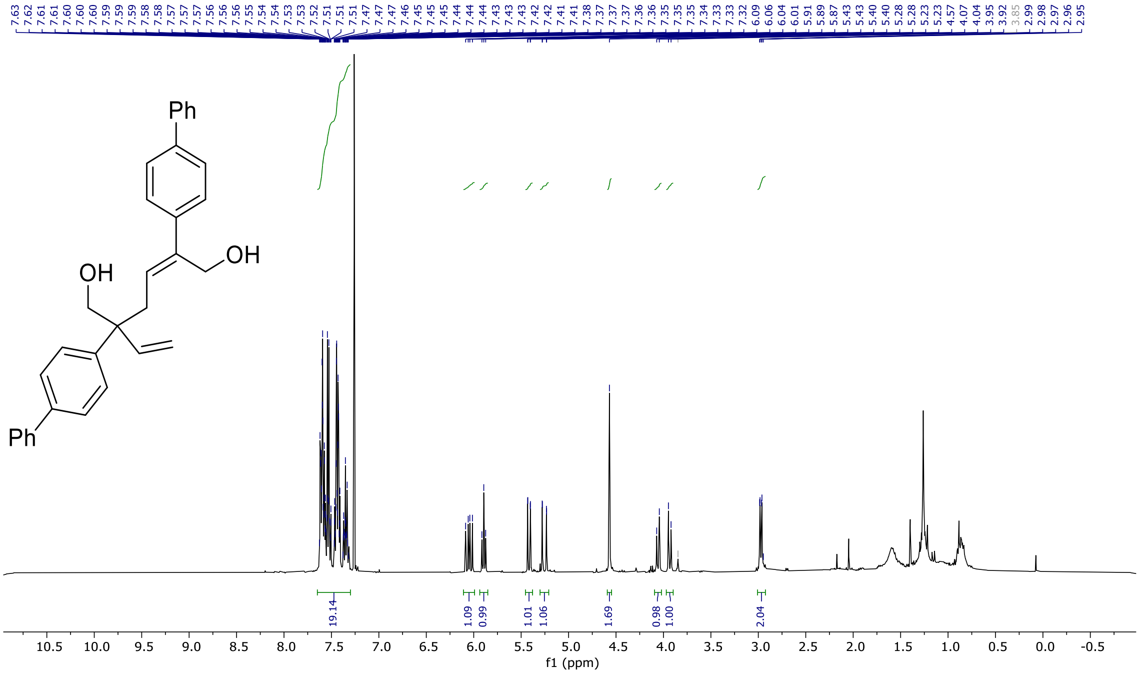

^{13}C NMR (101 MHz, $CDCl_3$)

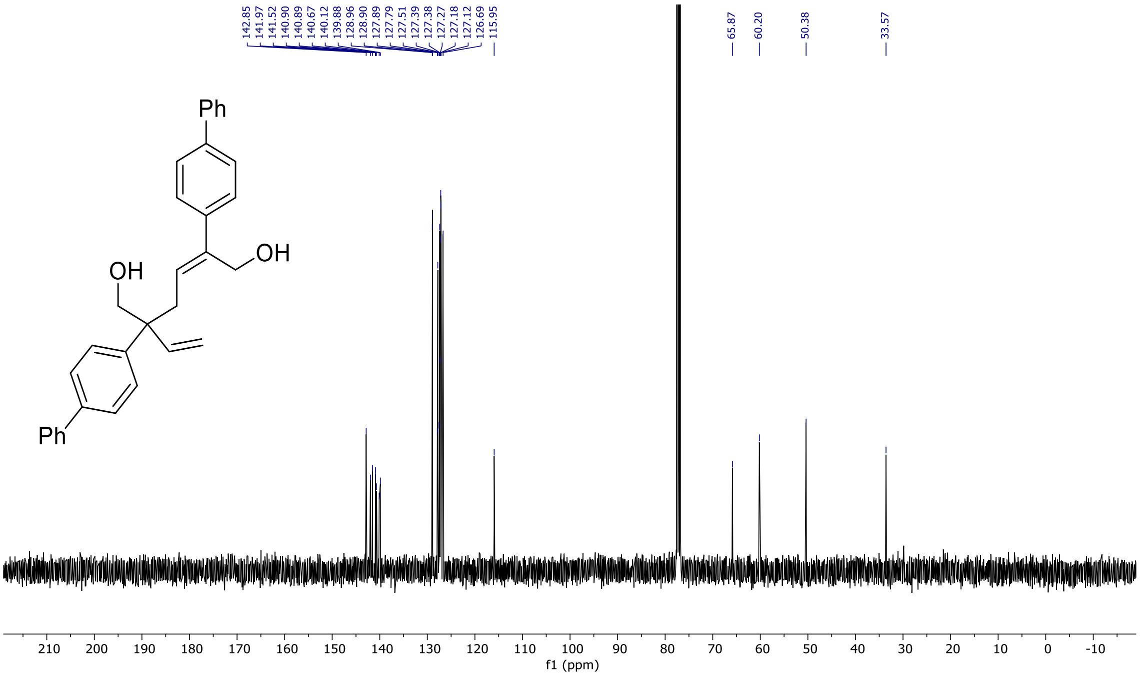

(*Z*)-2,5-di-*m*-tolyl-5-vinylhex-2-ene-1,6-diol (2k)

^{1}H NMR (400 MHz, $CDCl_3$)

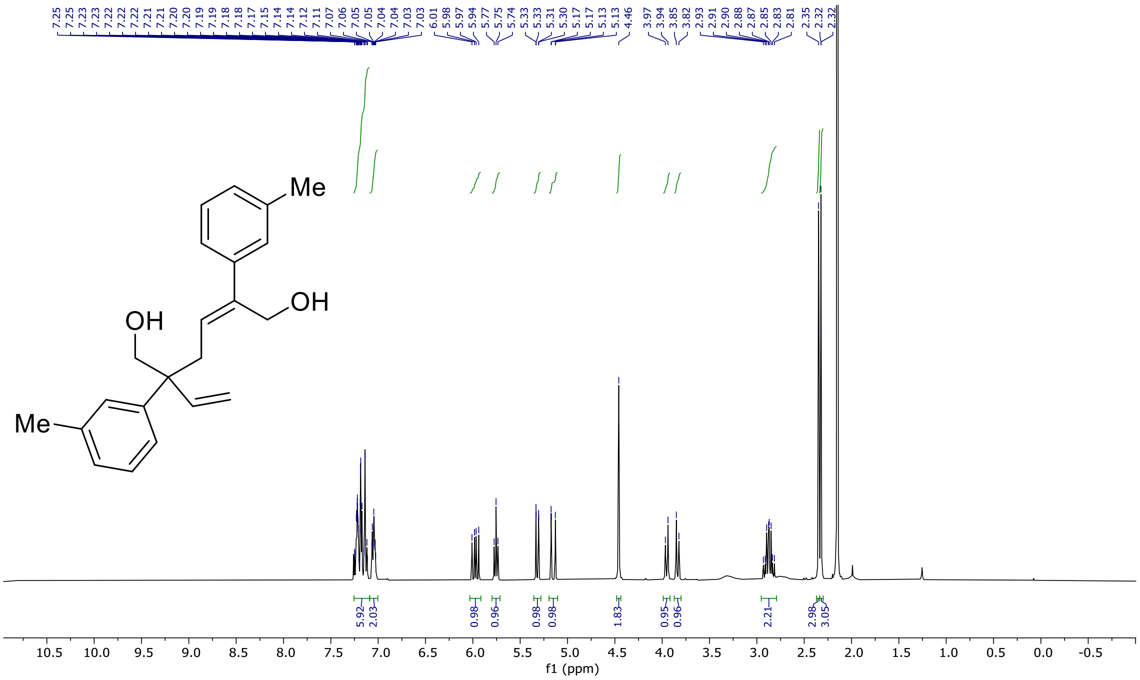

^{13}C NMR (101 MHz, $CDCl_3$)

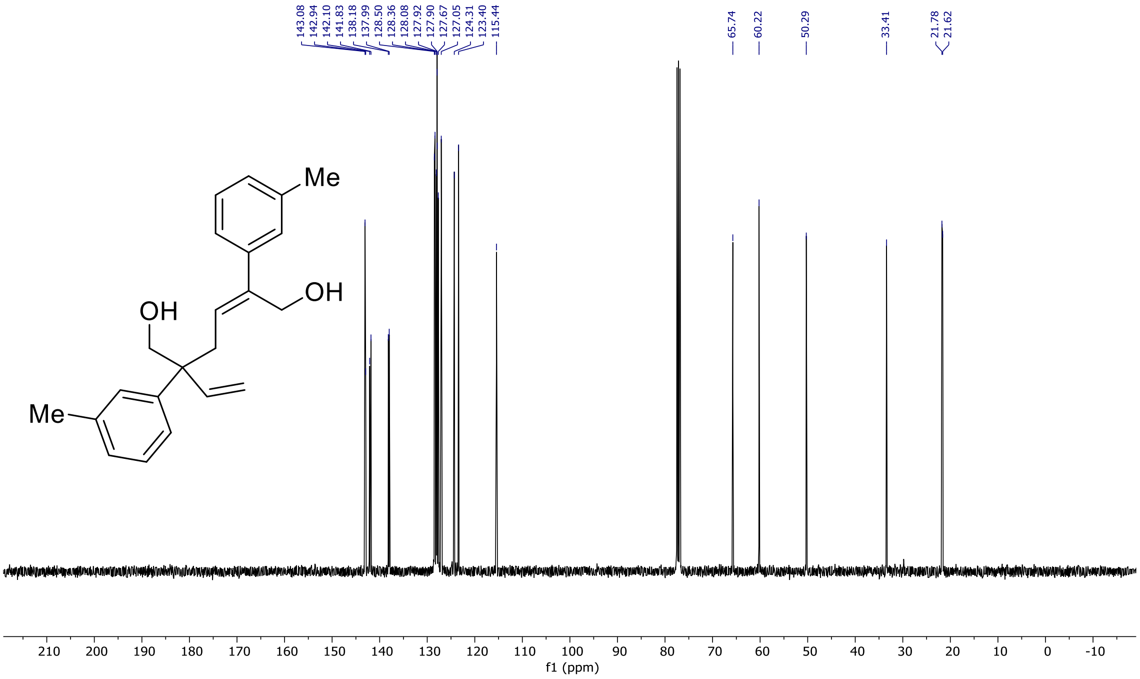

(*Z*)-2,5-bis(3-methoxyphenyl)-5-vinylhex-2-ene-1,6-diol (2l)

^{1}H NMR (400 MHz, $CDCl_3$)

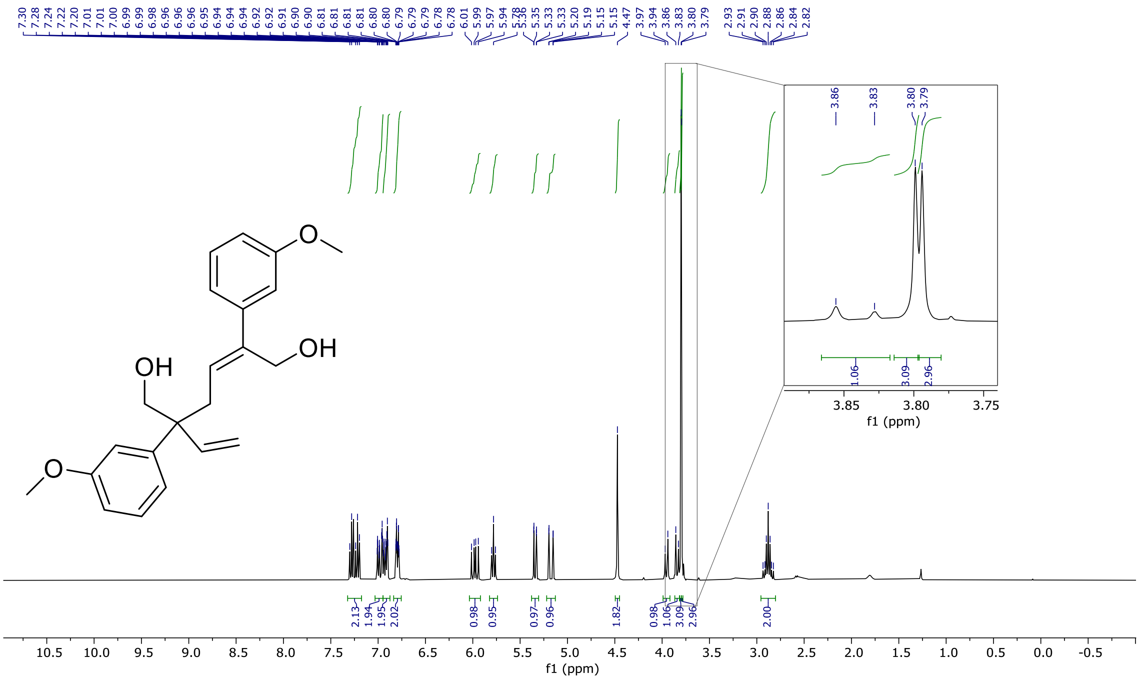

^{13}C NMR (101 MHz, $CDCl_3$)

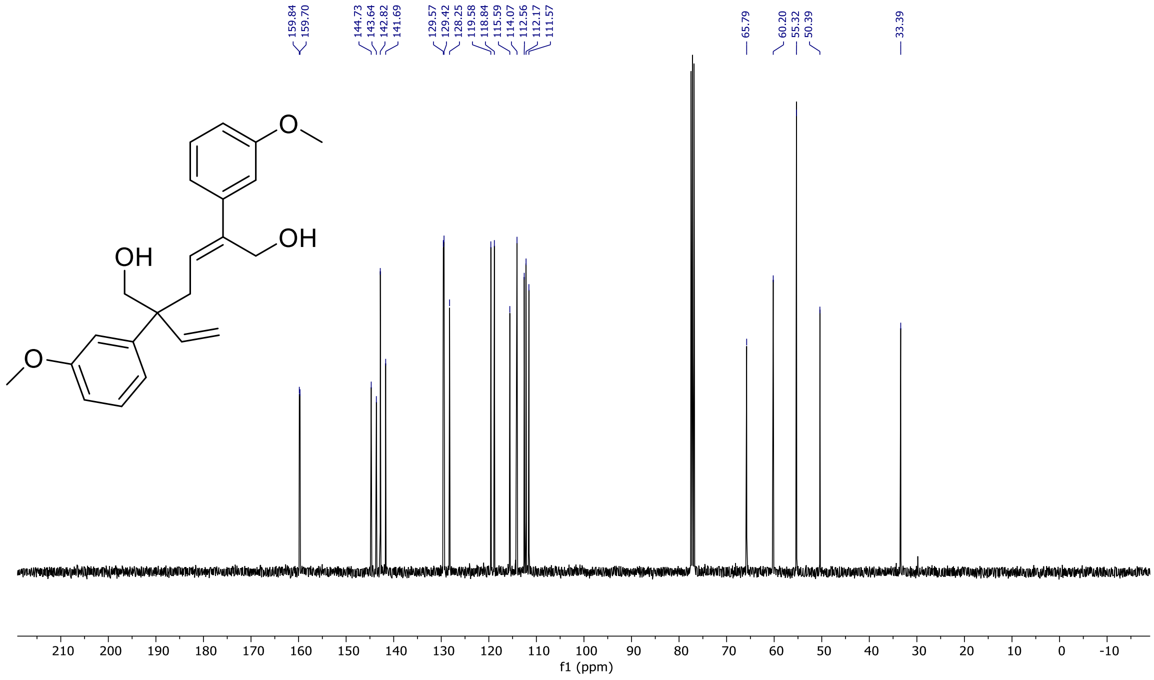

(*Z*)-2,5-bis(3-(benzyloxy)phenyl)-5-vinylhex-2-ene-1,6-diol (2m)

^{1}H NMR (400 MHz, $CDCl_3$)

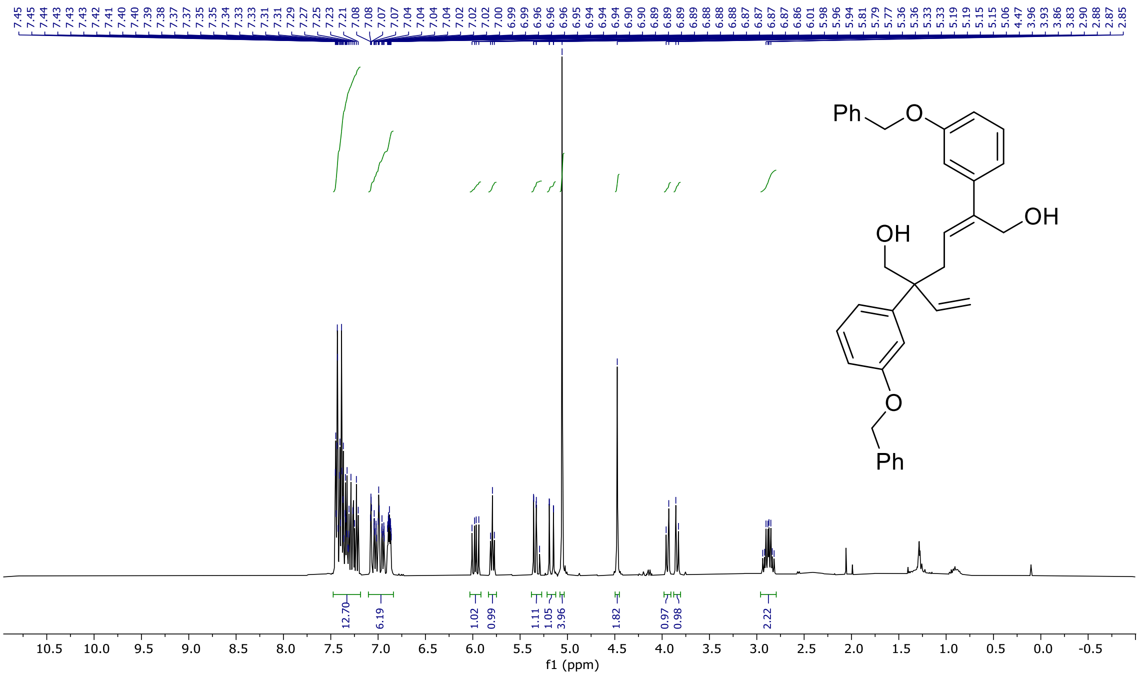

^{13}C NMR (101 MHz, $CDCl_3$)

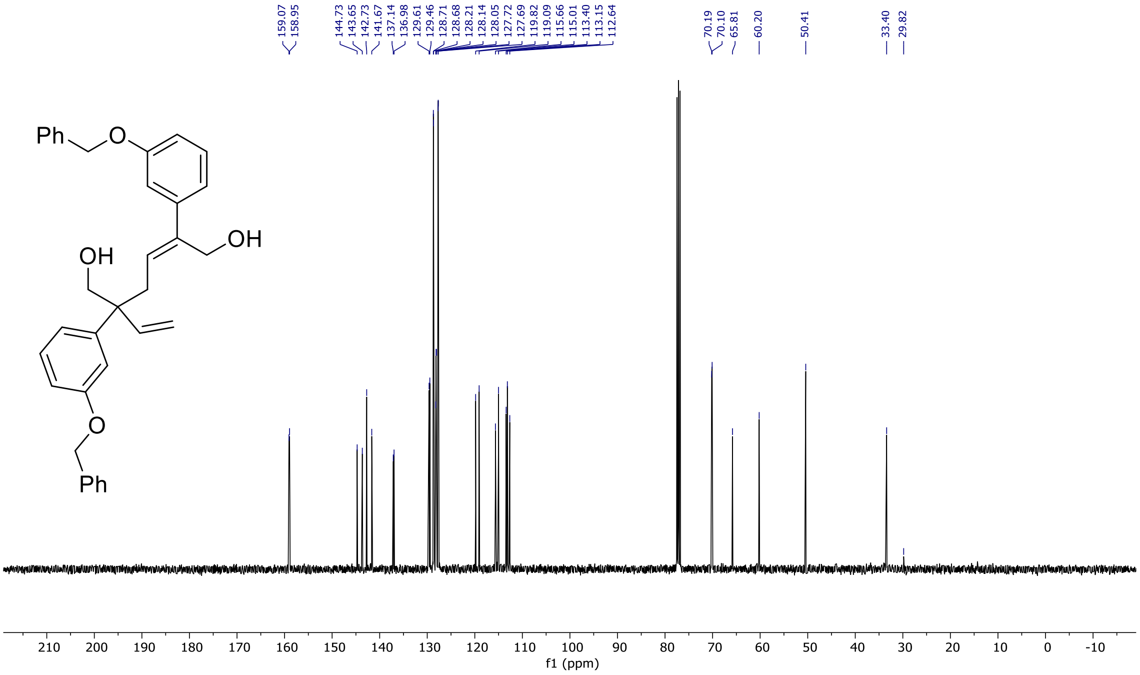

(*Z*)-2,5-bis(3-(trifluoromethyl)phenyl)-5-vinylhex-2-ene-1,6-diol (2n)

^{1}H NMR (400 MHz, $CDCl_3$)

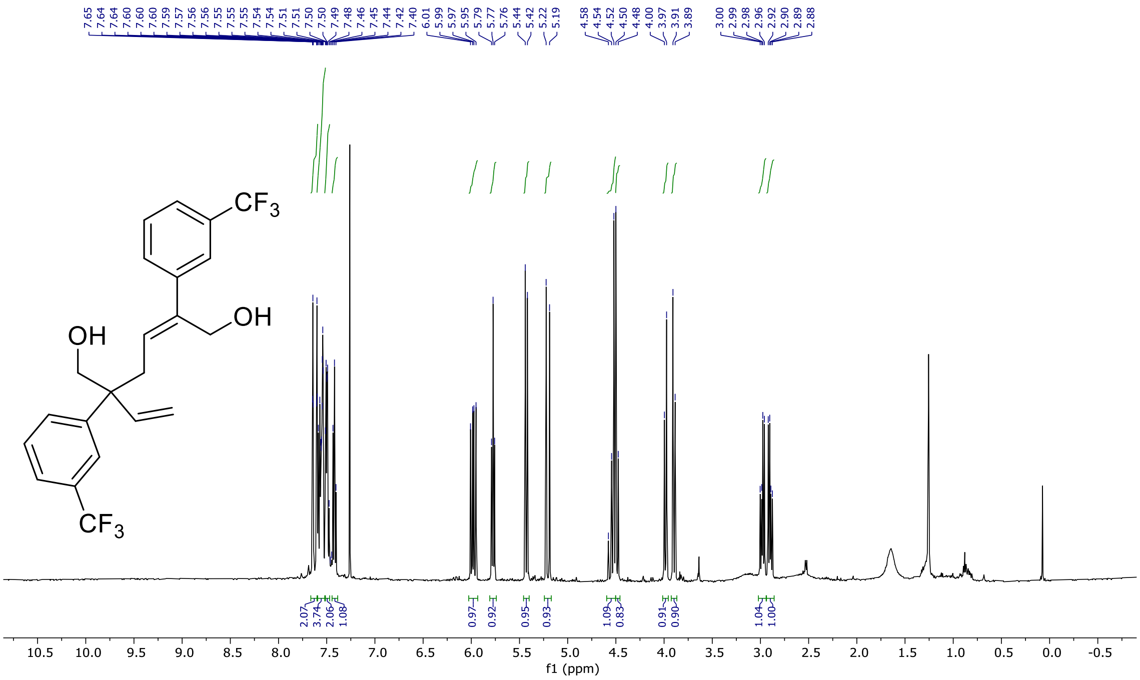

^{13}C NMR (101 MHz, $CDCl_3$)

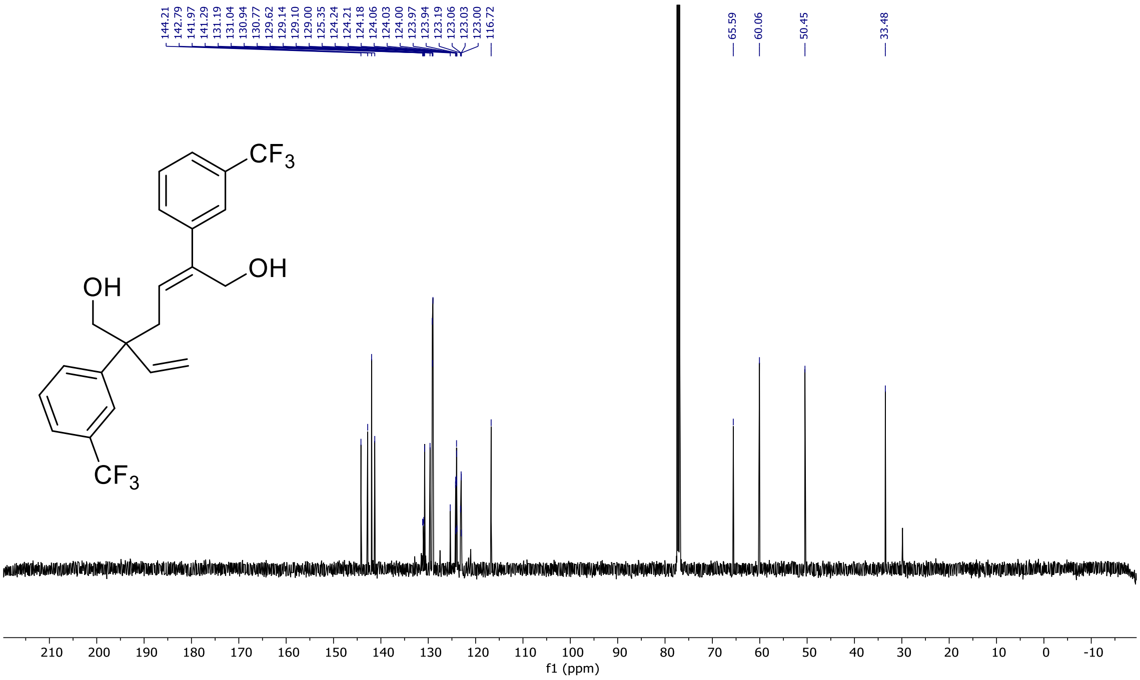

^{19}F NMR (376 MHz, $CDCl_3$)

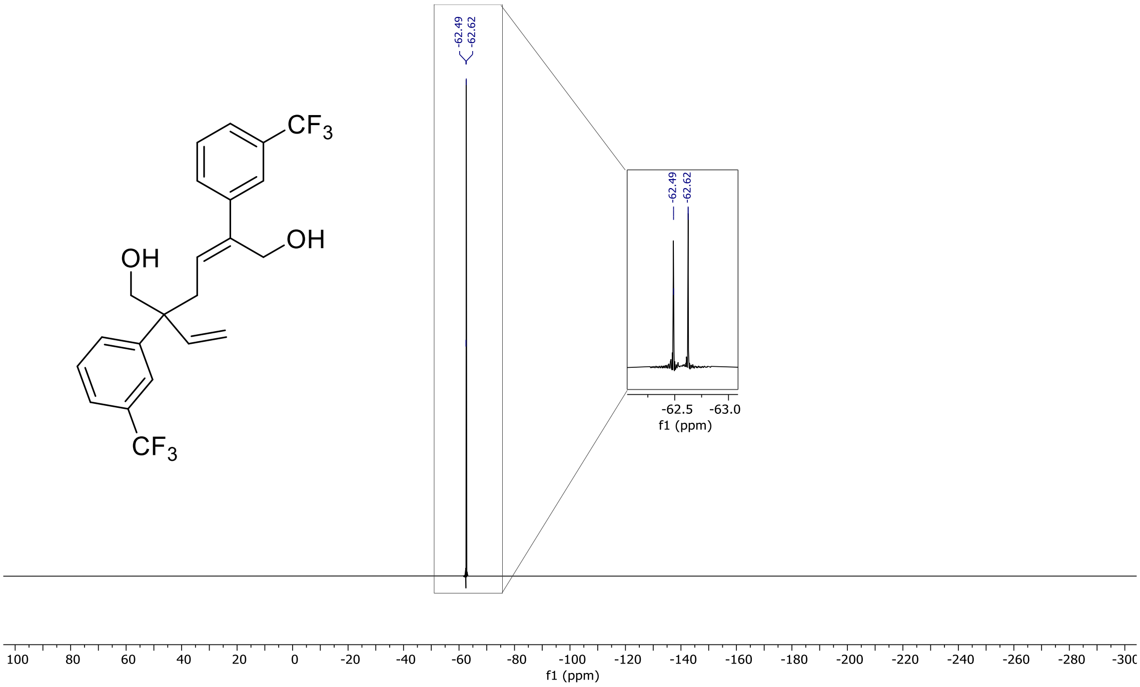

(*Z*)-2,5-di(thiophen-3-yl)-5-vinylhex-2-ene-1,6-diol (2o)

^{1}H NMR (400 MHz, $CDCl_3$)

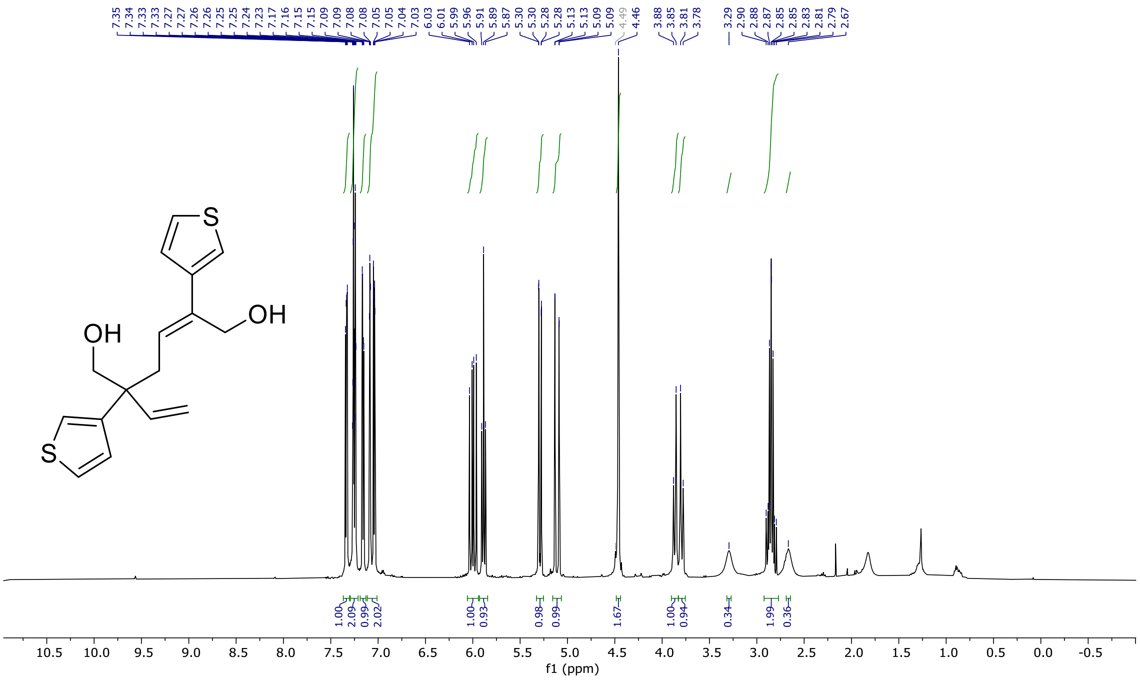

^{13}C NMR (101 MHz, $CDCl_3$)

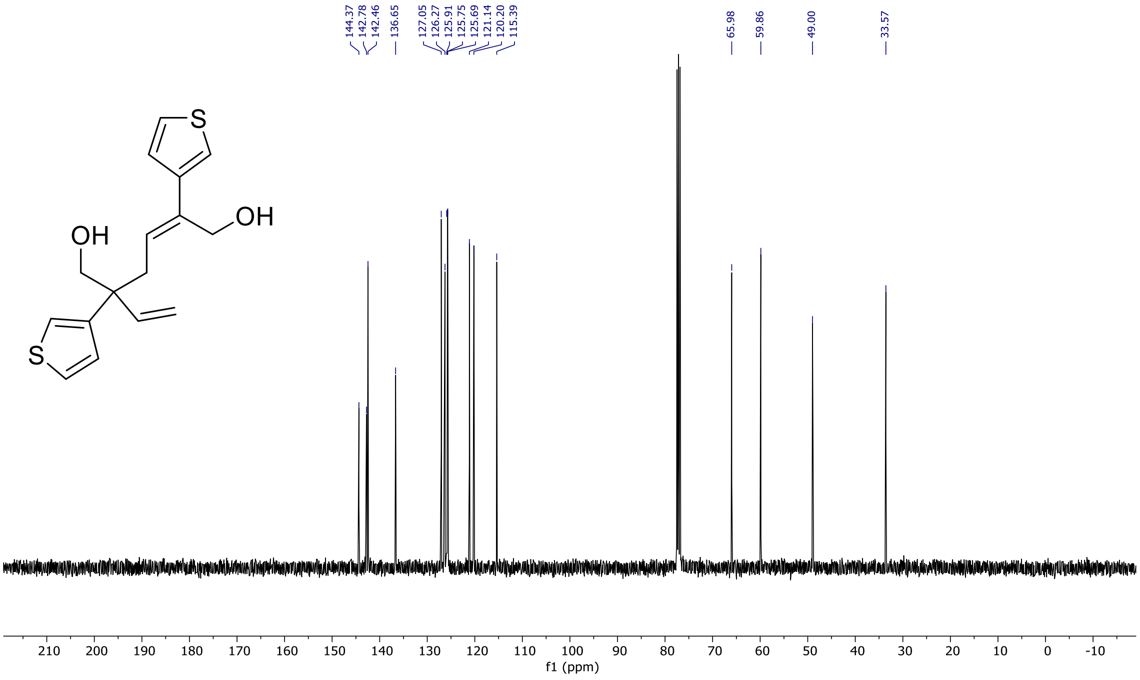

(*E*)-2,5-di(furan-2-yl)-5-vinylhex-2-ene-1,6-diol (2p)

^{1}H NMR (400 MHz, CD_3CN)

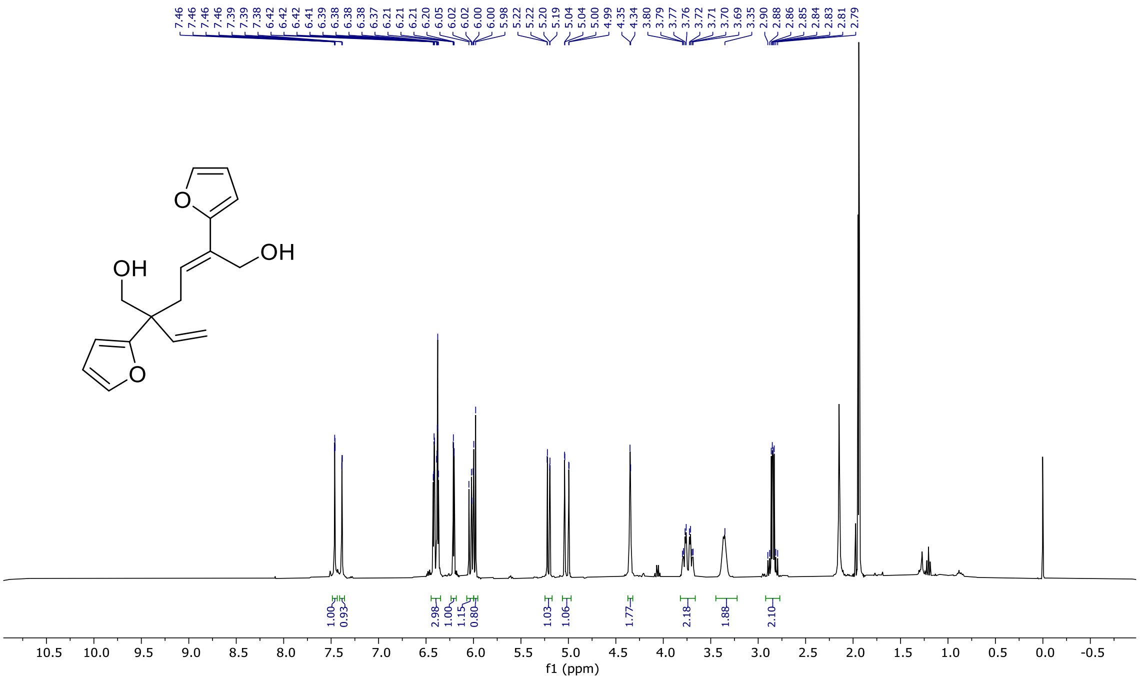

^{13}C NMR (101 MHz, CD_3CN)

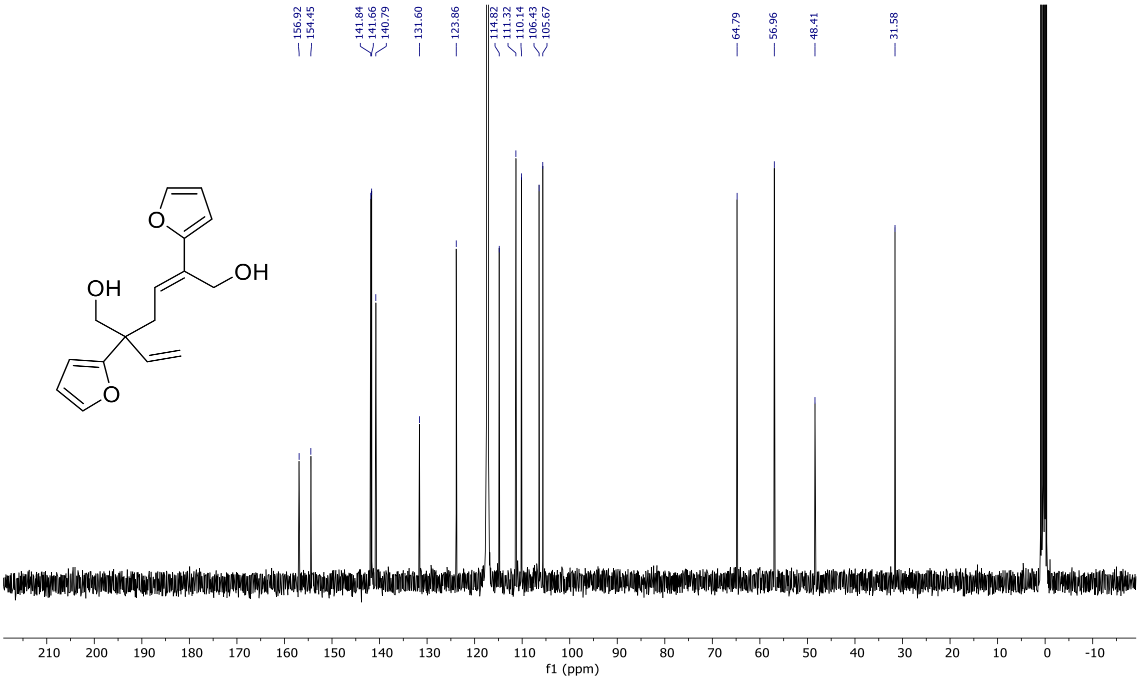

(*Z*)-2,5-di(naphthalen-2-yl)-5-vinylhex-2-ene-1,6-diol (2q)

^{1}H NMR (400 MHz, $CDCl_3$)

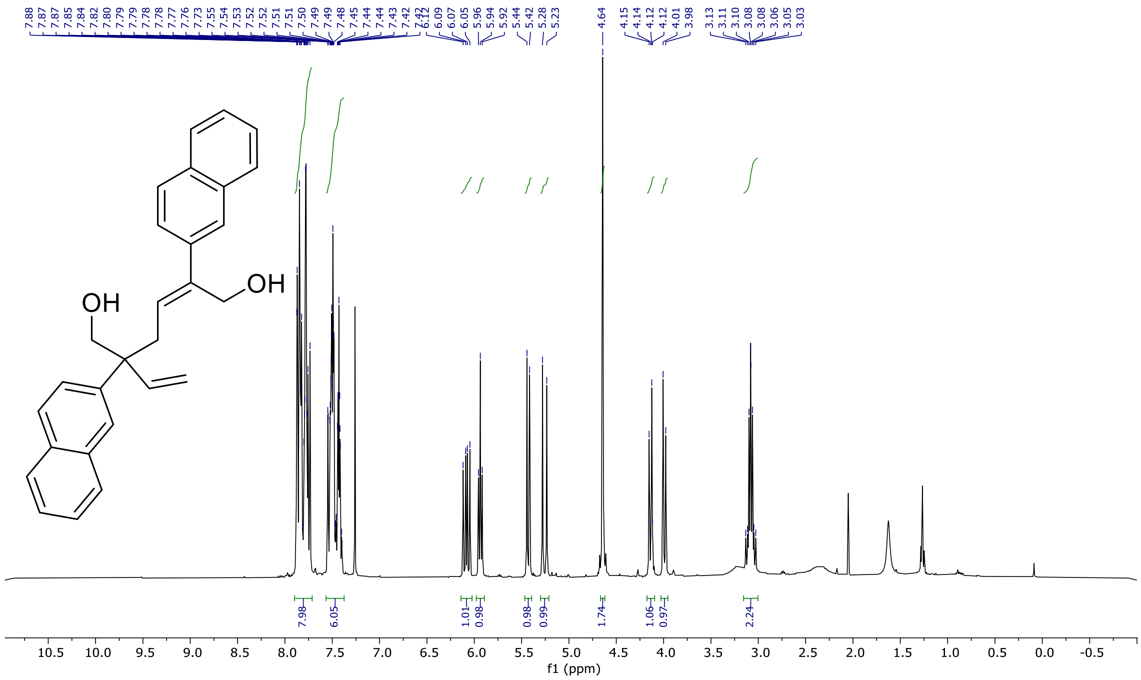

^{13}C NMR (101 MHz, $CDCl_3$)

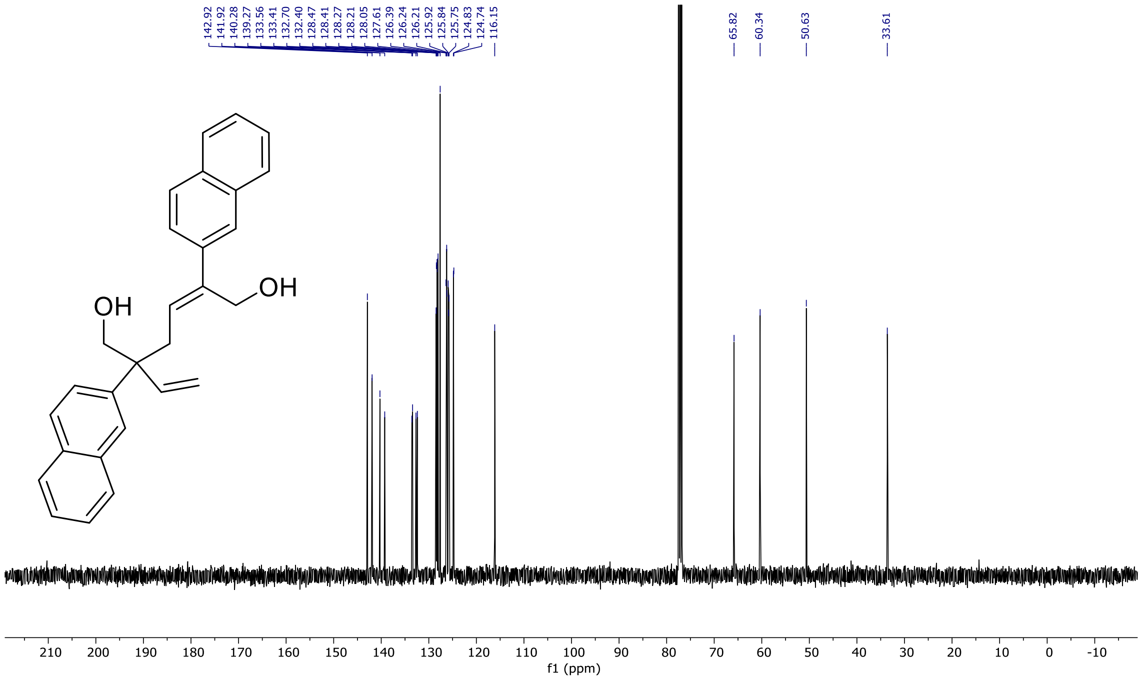

(*Z*)-2,5-bis(3,5-dimethylphenyl)-5-vinylhex-2-ene-1,6-diol (2r)

^{1}H NMR (400 MHz, $CDCl_3$)

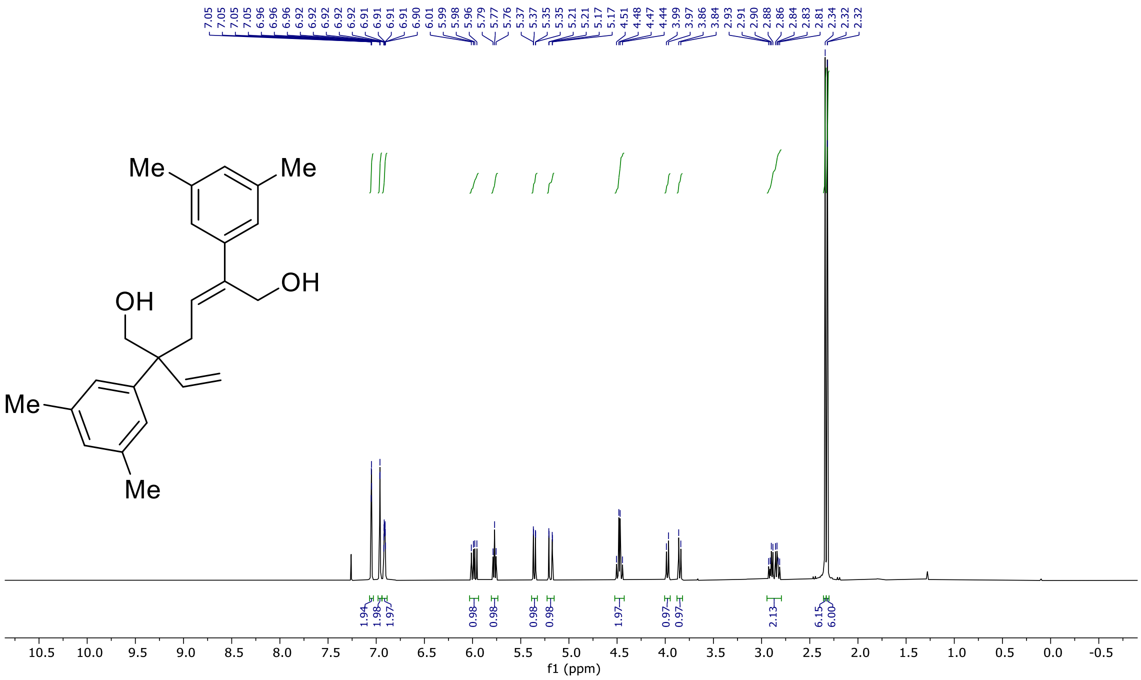

^{13}C NMR (101 MHz, $CDCl_3$)

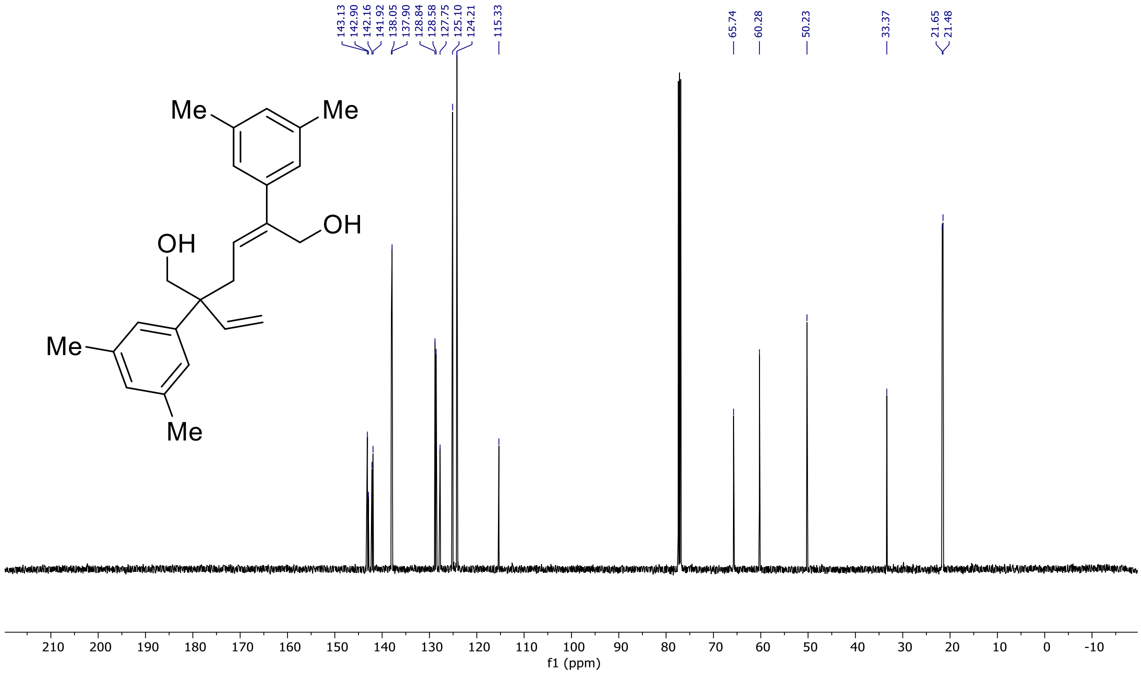

(*Z*)-2,5-bis(3,5-dimethoxyphenyl)-5-vinylhex-2-ene-1,6-diol (2s)

^{1}H NMR (400 MHz, $CDCl_3$)

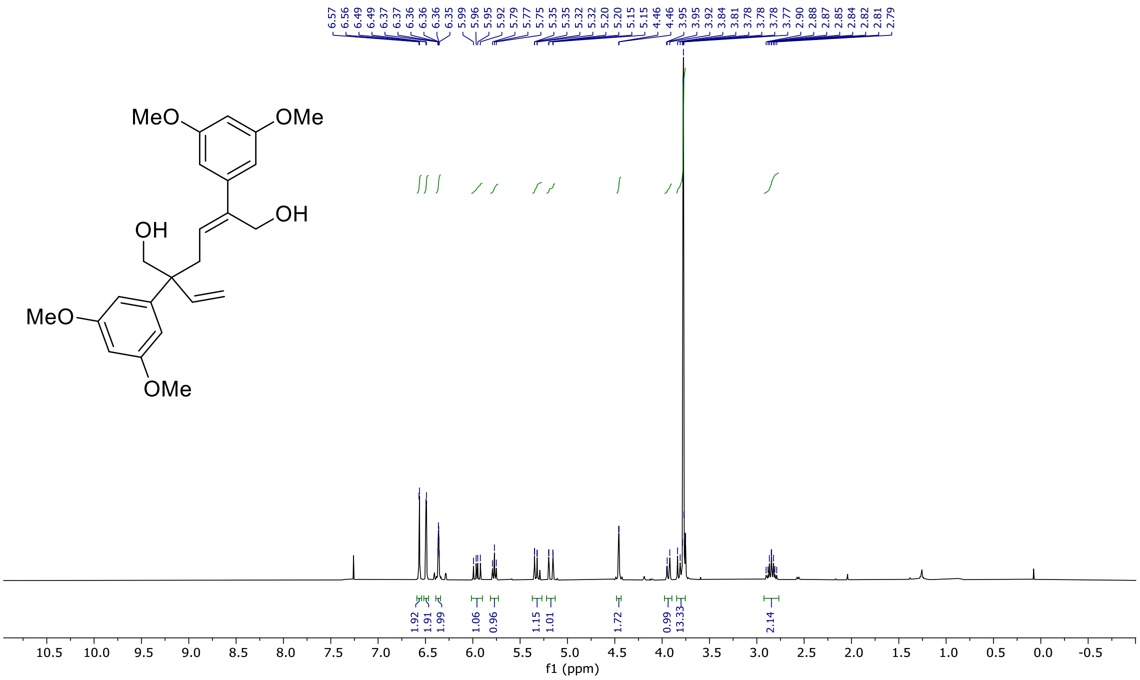

^{13}C NMR (101 MHz, $CDCl_3$)

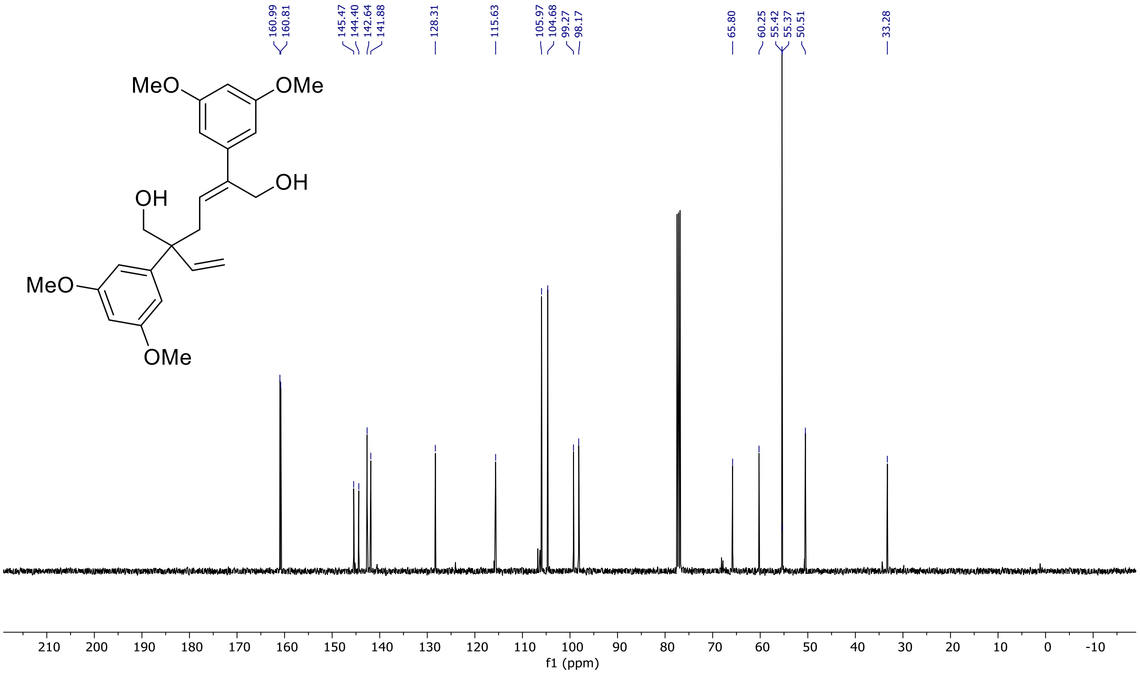

(*Z*)-2,5-bis(benzo[*d*][1,3]dioxol-5-yl)-5-vinylhex-2-ene-1,6-diol (2t)

^{1}H NMR (400 MHz, $CDCl_3$)

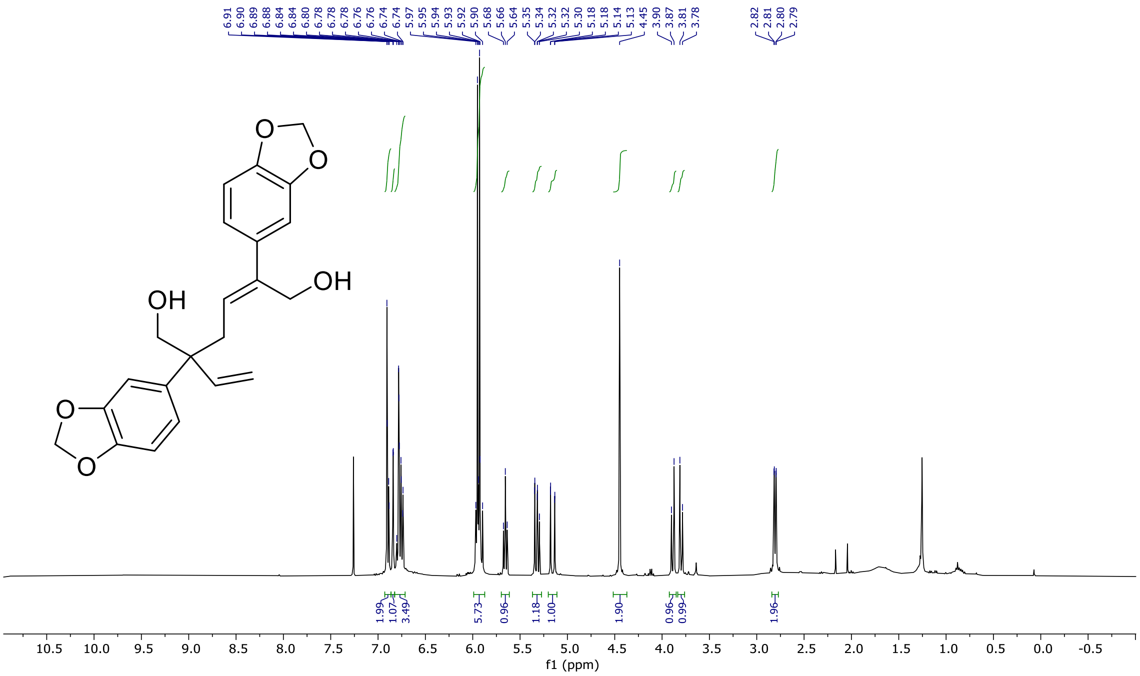

^{13}C NMR (101 MHz, $CDCl_3$)

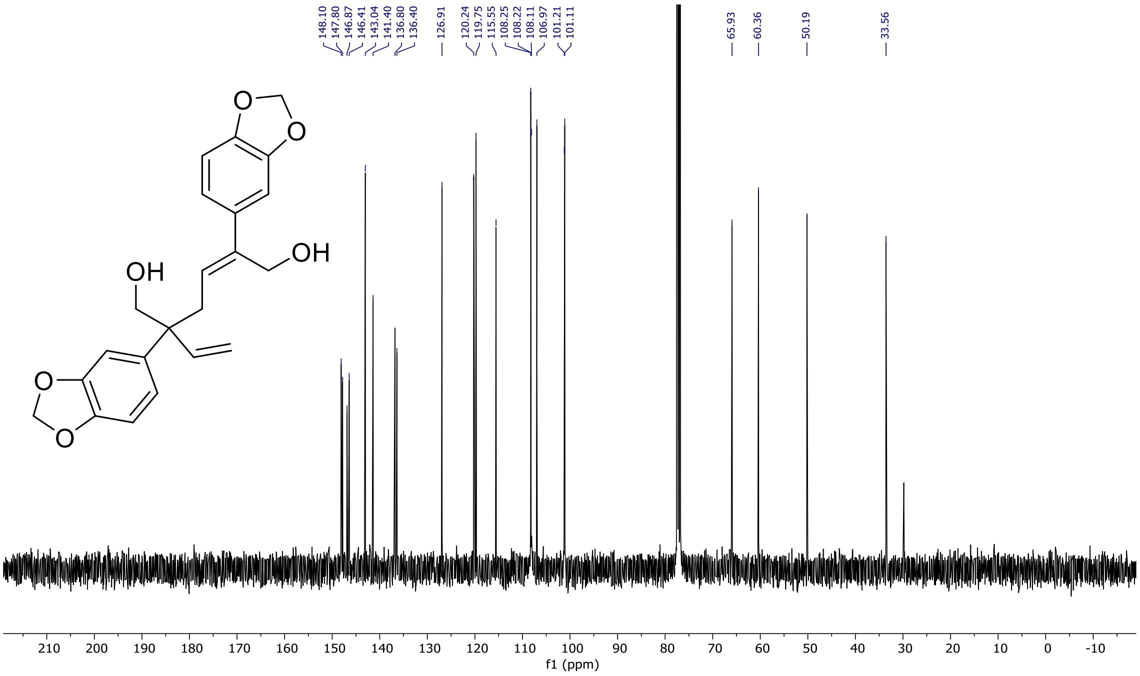

(Z)-2,5-diphenyl-5-vinylhex-2-ene-1,6-diyl diacetate (5)

^{1}H NMR (400 MHz, $CDCl_3$)

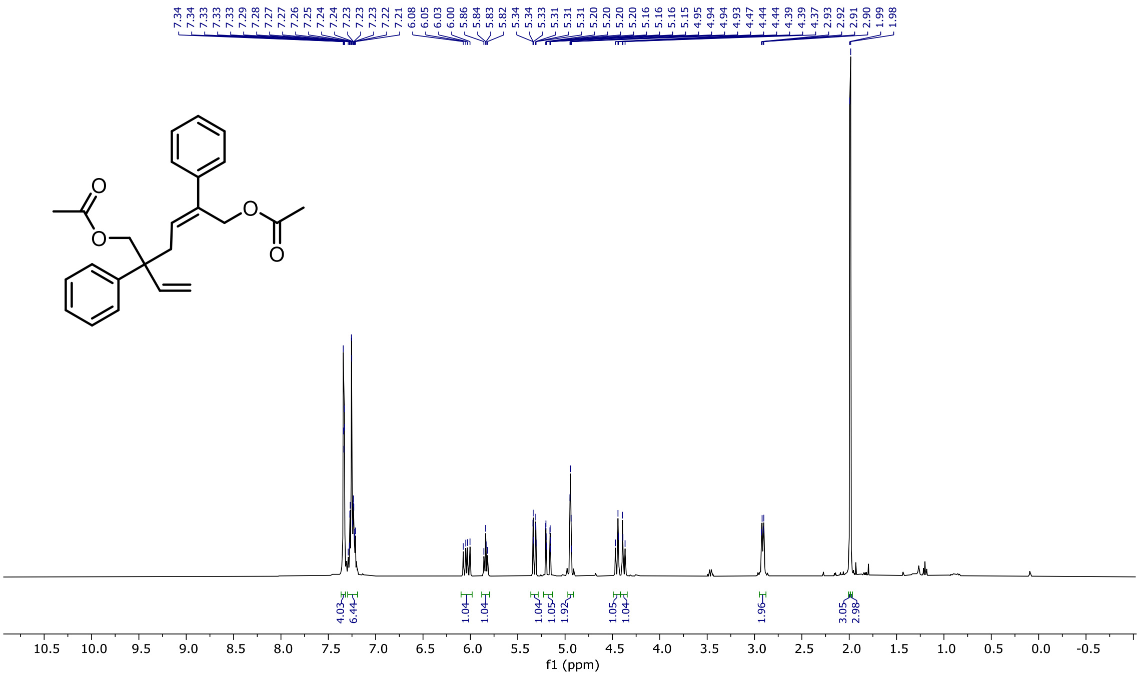

^{13}C NMR (101 MHz, $CDCl_3$)

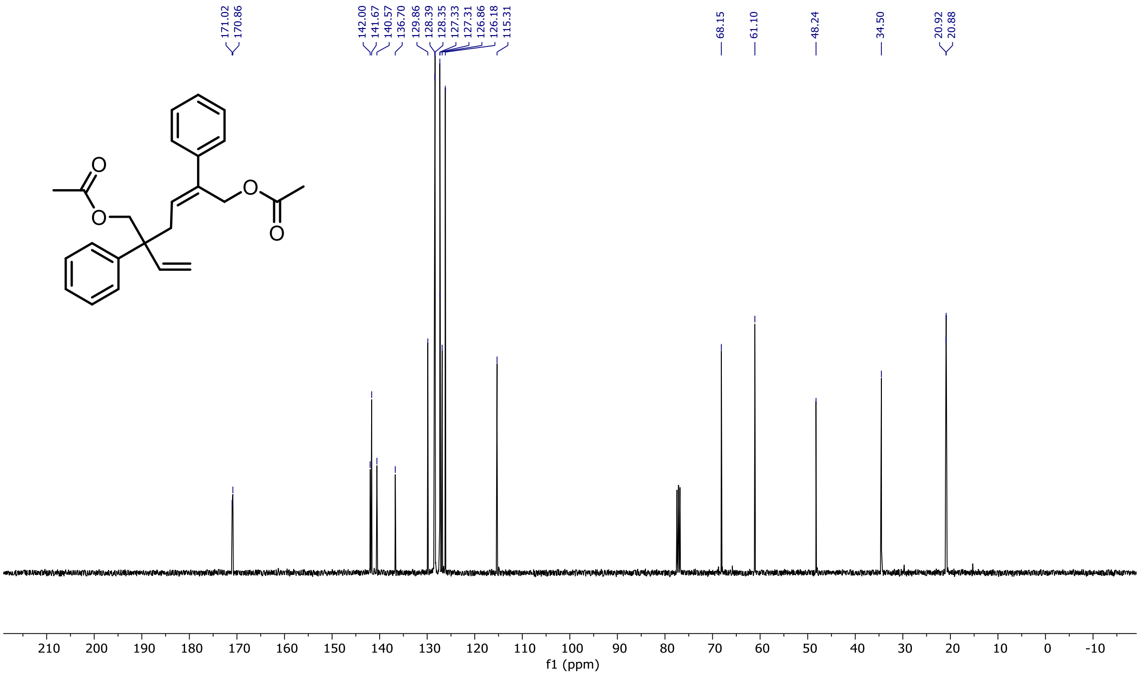

(3-(2-(acetoxymethyl)-2-phenylbut-3-en-1-yl)-2-phenyloxiran-2-yl)methyl acetate (6)

^{1}H NMR (400 MHz, $CDCl_3$)

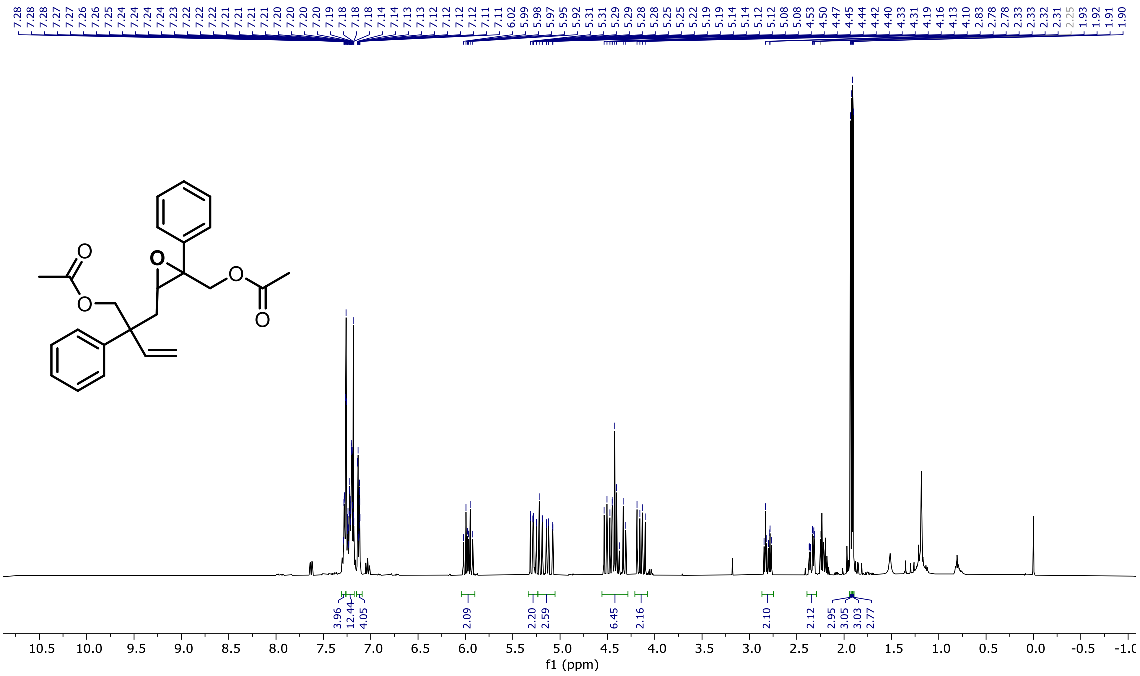

^{13}C NMR (101 MHz, $CDCl_3$)

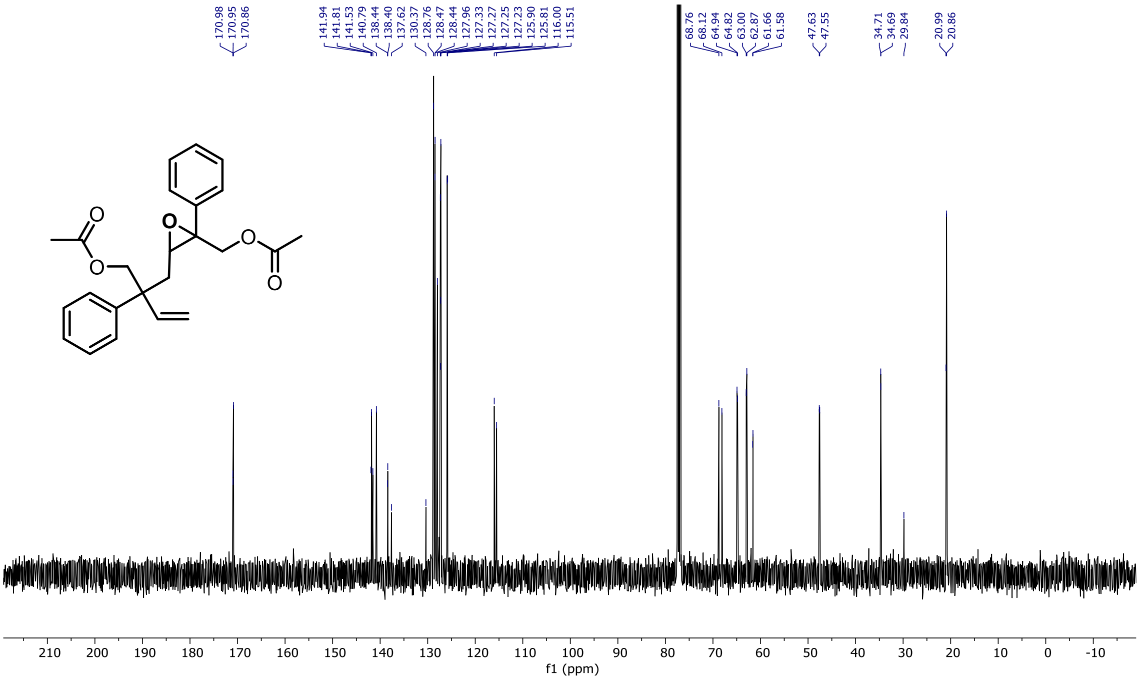

6-(1,3-dioxoisoindolin-2-yl)-2,5-diphenyl-2-vinylhex-4-en-1-yl acetate (7)

^{1}H NMR (400 MHz, $CDCl_3$)

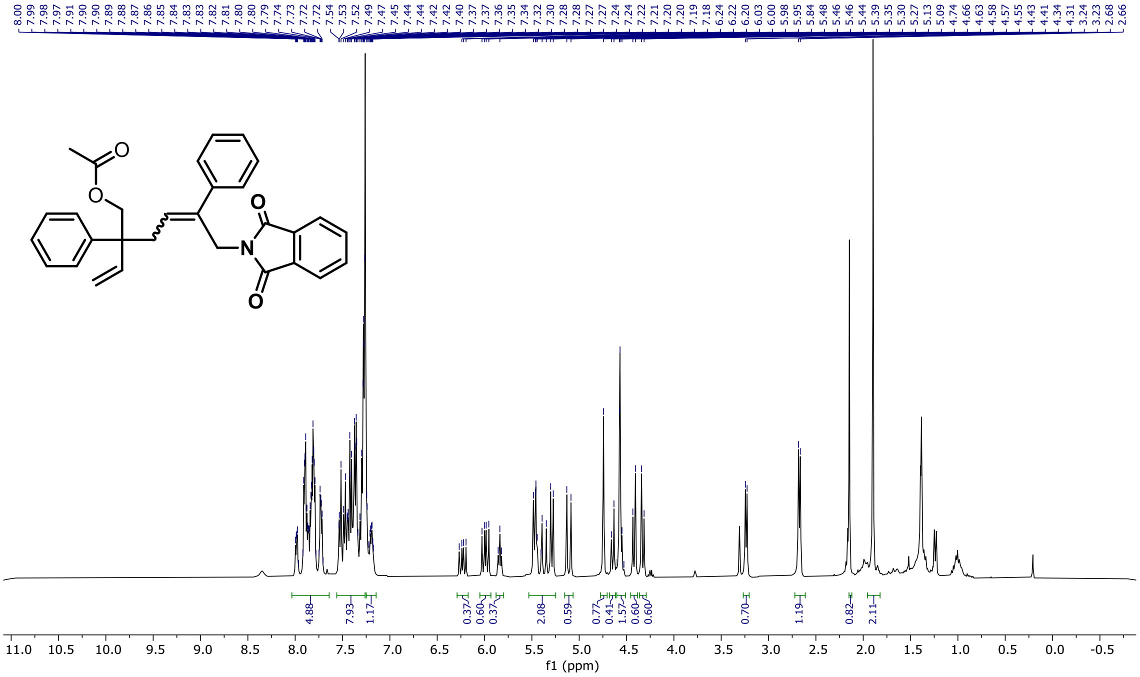

^{13}C NMR (101 MHz, $CDCl_3$)

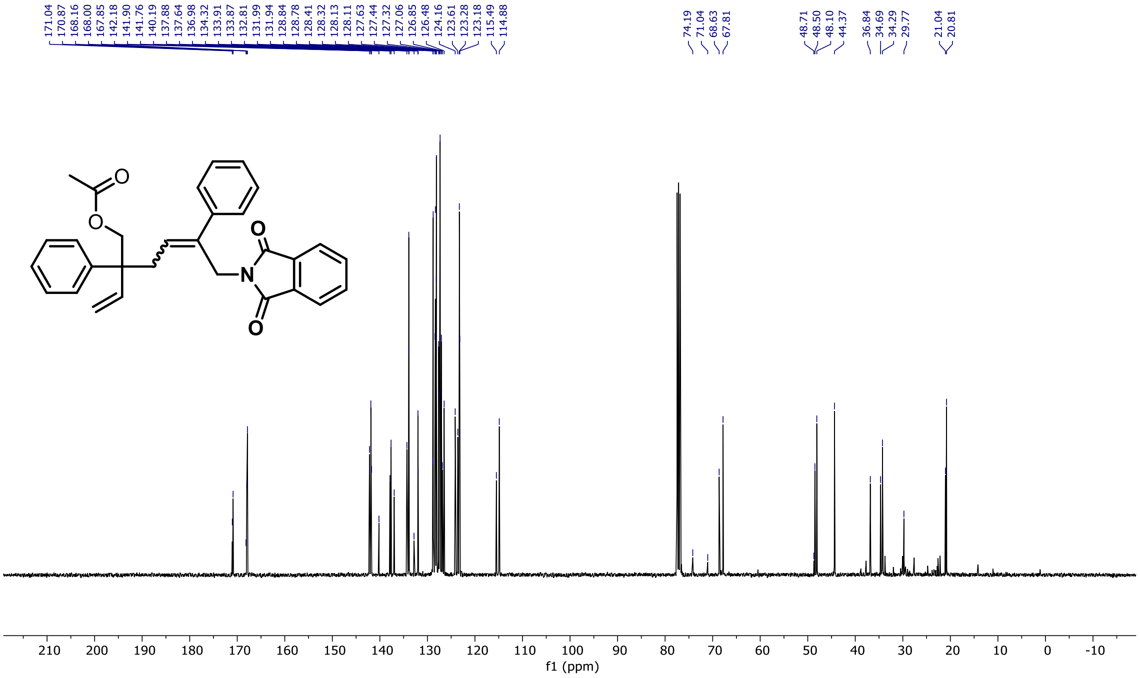

(*Z*)-2,5-diphenyl-5-vinylhex-2-enedial (*Z*-8)

^{1}H NMR (400 MHz, $CDCl_3$)

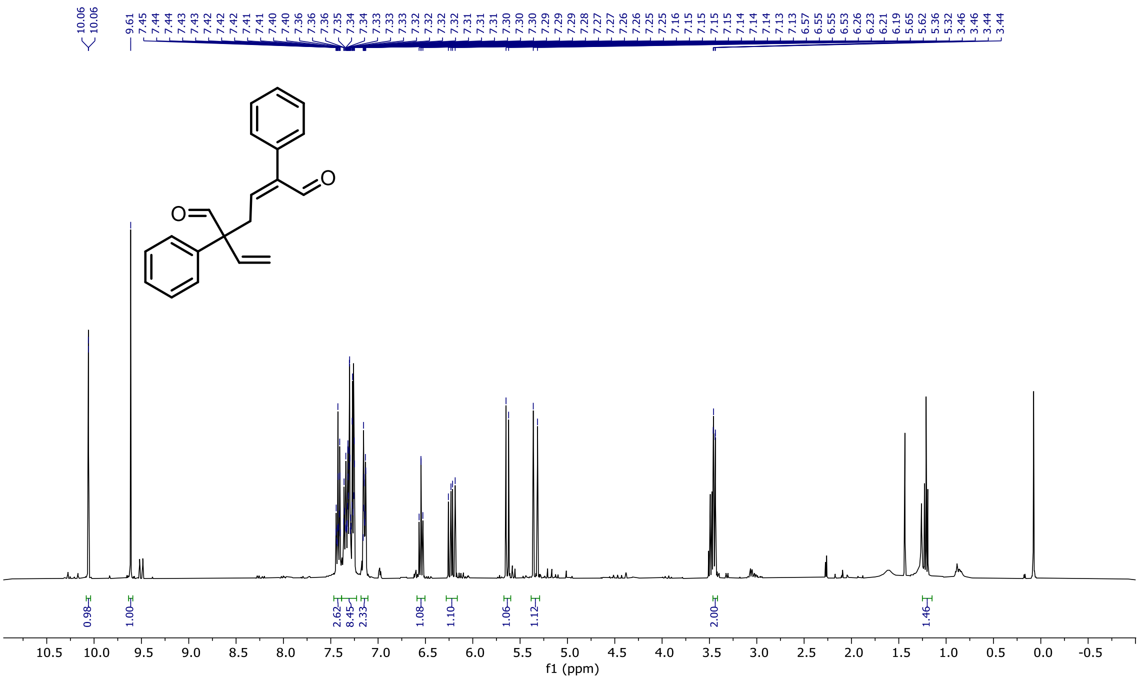

^{13}C NMR (101 MHz, $CDCl_3$)

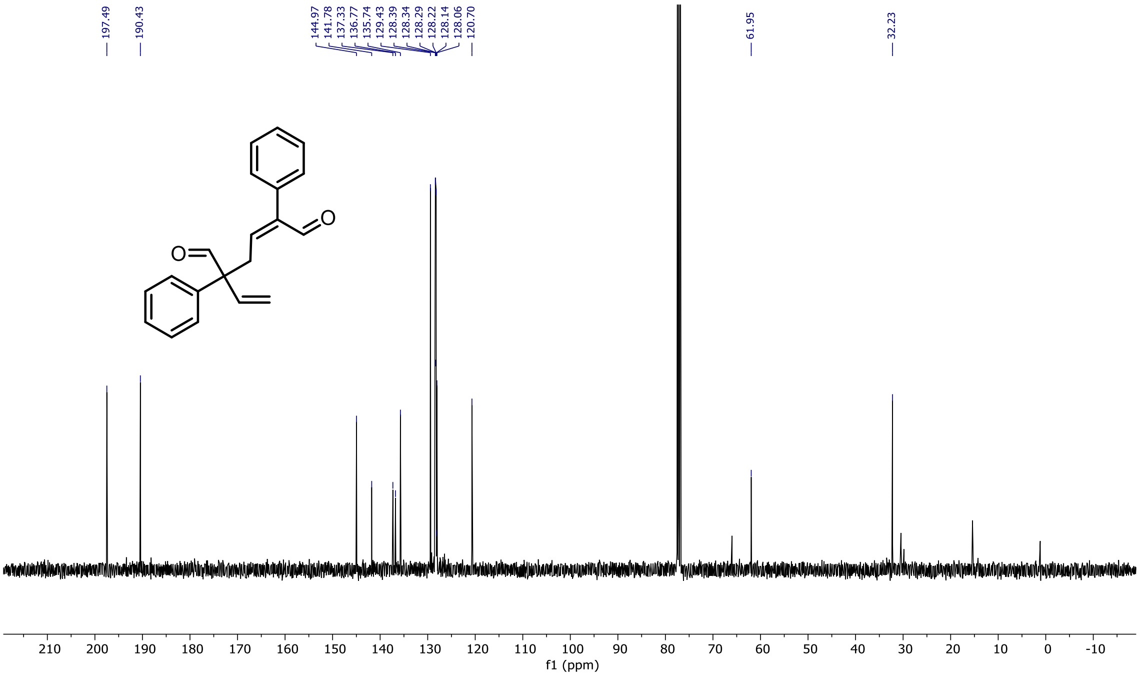

(*E*)-2,5-diphenyl-5-vinylhex-2-enedial (*E*-8)

^{1}H NMR (400 MHz, $CDCl_3$)

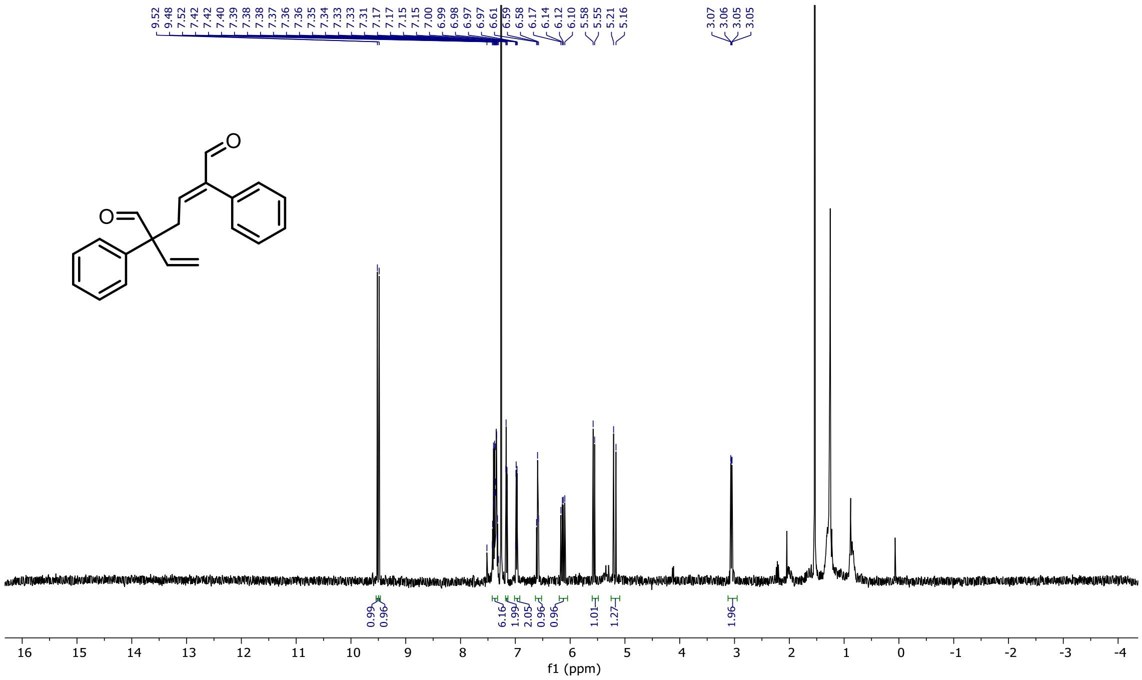

^{13}C NMR (101 MHz, $CDCl_3$)

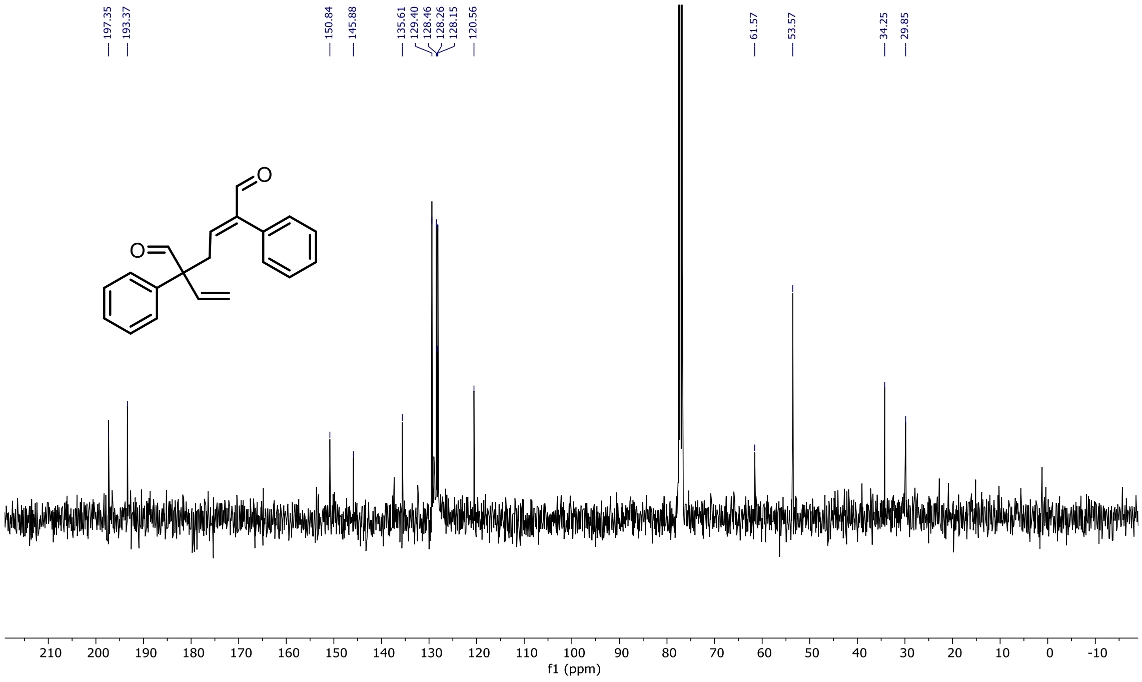

(*E*)-3,6-diphenyl-6-vinyloct-3-ene-1,8-diol (*bl*-10a)

^{1}H NMR (400 MHz, $CDCl_3$)

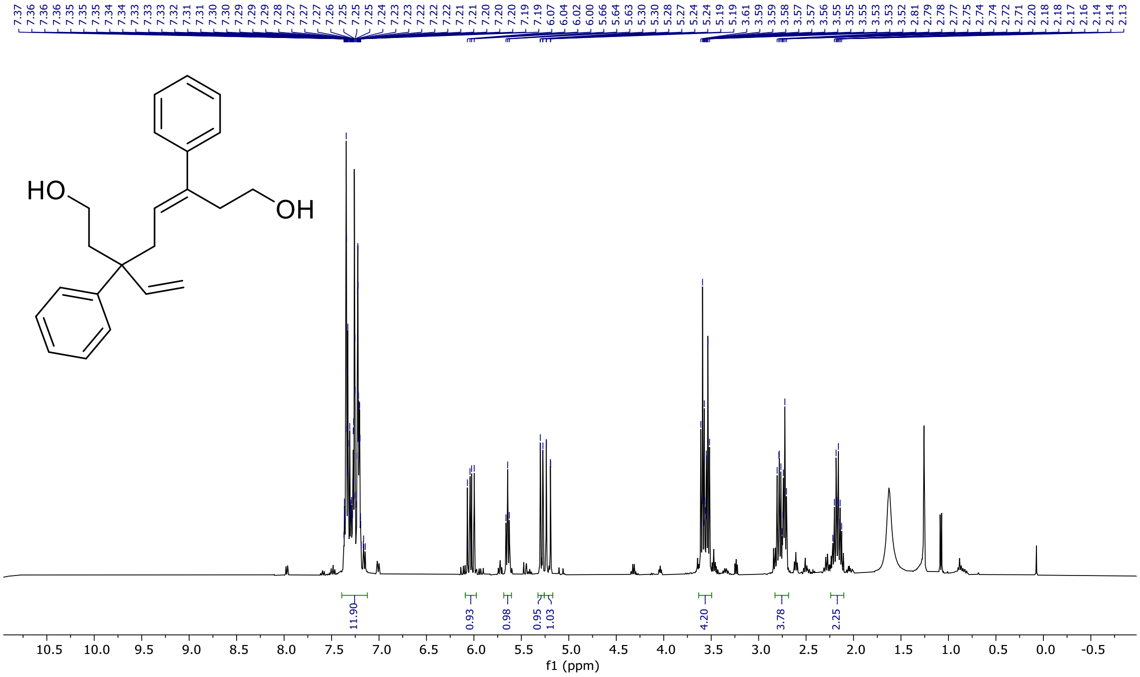

^{13}C NMR (101 MHz, $CDCl_3$)

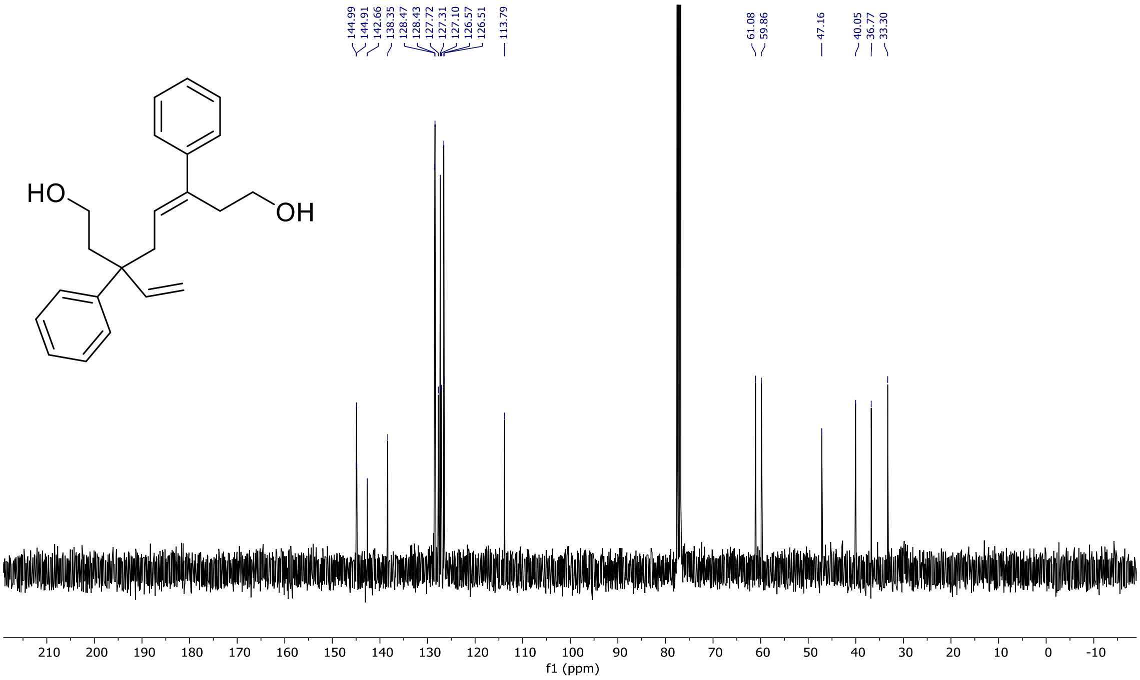

(3*E*,7*E*)-3,8-diphenyl-dec-3,7-diene-1,10-diol (*ll*-10a)

^{1}H NMR (400 MHz, $CDCl_3$)

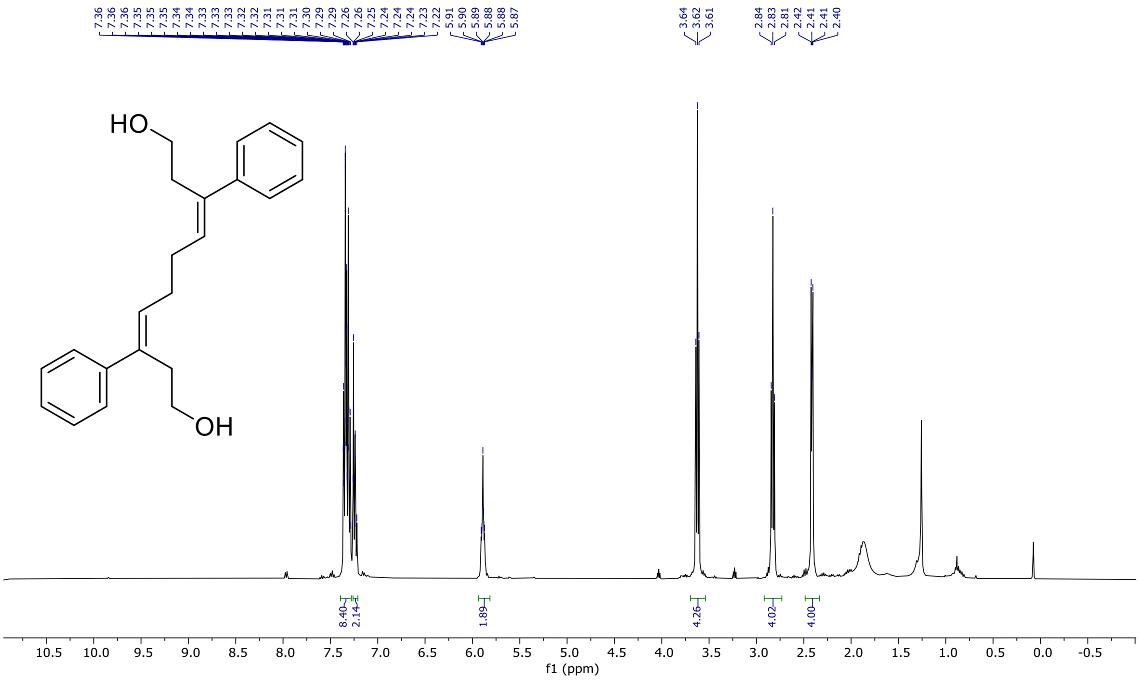

^{13}C NMR (101 MHz, $CDCl_3$)

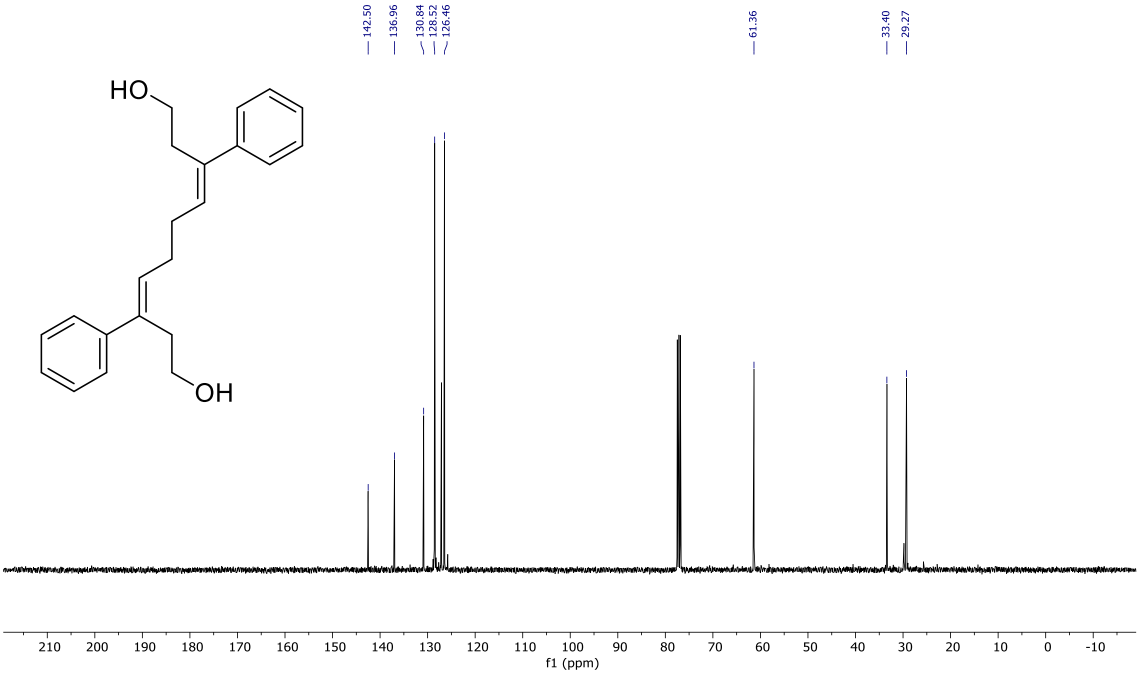

Supplement: Supplementary file 1 [file ol6c02074_si_001.pdf]
